# Transcriptomics supports local sensory regulation in the antenna of the kissing-bug *Rhodnius prolixus*

**DOI:** 10.1186/s12864-020-6514-3

**Published:** 2020-01-30

**Authors:** Jose Manuel Latorre-Estivalis, Marcos Sterkel, Sheila Ons, Marcelo Gustavo Lorenzo

**Affiliations:** 1 Vector Behaviour and Pathogen Interaction Group, Instituto René Rachou – FIOCRUZ, Belo Horizonte, Minas Gerais Brazil; 2 Laboratorio de Neurobiología de Insectos - Centro Regional de Estudios Genómicos – CREG, Facultad de Ciencias Exactas. Universidad Nacional de La Plata, La Plata, Buenos Aires Argentina

**Keywords:** Antennae, Transcriptomics, Kissing-bugs, Neuropeptides

## Abstract

**Background:**

*Rhodnius prolixus* has become a model for revealing the molecular bases of insect sensory biology due to the publication of its genome and its well-characterized behavioural repertoire. Gene expression modulation underlies behaviour-triggering processes at peripheral and central levels. Still, the regulation of sensory-related gene transcription in sensory organs is poorly understood. Here we study the genetic bases of plasticity in antennal sensory function, using *R. prolixus* as an insect model.

**Results:**

Antennal expression of neuromodulatory genes such as those coding for neuropeptides, neurohormones and their receptors was characterized in fifth instar larvae and female and male adults by means of RNA-Sequencing (RNA-Seq). New nuclear receptor and *takeout* gene sequences were identified for this species, as well as those of enzymes involved in the biosynthesis and processing of neuropeptides and biogenic amines.

**Conclusions:**

We report a broad repertoire of neuromodulatory and neuroendocrine-related genes expressed in the antennae of *R. prolixus* and suggest that they may serve as the local basis for modulation of sensory neuron physiology. Diverse neuropeptide precursor genes showed consistent expression in the antennae of all stages studied. Future studies should characterize the role of these modulatory components acting over antennal sensory processes to assess the relative contribution of peripheral and central regulatory systems on the plastic expression of insect behaviour.

## Background

*Rhodnius prolixus* has been an important insect model for neuroethological studies for many decades [1, 2]. Relevant aspects of its neuroethology and physiology, such as host odour-mediated behaviour [3, 4], circadian modulation [5], the action of biogenic amines and neuropeptides [6, 7] or the expression of behavioural plasticity [8, 9] have been thoroughly studied. Recently, molecular processes related to sensory function have been characterized for *R. prolixus*, such as the tissue-specific expression profiles of odorant receptor genes [10] and related changes associated with development and nutrition [11]. Additionally, neuropeptide precursor genes were described for *R. prolixus* [12] and the dynamics of neuropeptide expression or release at diverse physiological conditions were characterized for processes such as feeding or ecdysis [13, 14]. Based on the current knowledge on its behaviour and physiology, and the publication of its genome sequence [15], it is reasonable to suggest that *R. prolixus* has become an appropriate model for revealing the molecular bases of neuroethological processes in insects. Furthermore, neuroethological research in kissing-bugs is of medical interest given their role as vectors of *Trypanosoma cruzi*, the causative agent of Chagas’ disease, which is considered a neglected illness affecting over 8 million people worldwide (http://www.who.int/chagas/disease/en/).

Kissing-bug antennae are multimodal sensory organs dedicated to detect diverse stimuli associated with hosts [3], microenvironmental features and intraspecific communication [1]. The physiological bases of sensory processes sit on receptor neurons that express specific membrane proteins that confer them the ability to react to relevant environmental stimuli. These neurons are mostly found in tiny hair-like structures called sensilla, which can house from one to several dozen sensory cells [16]. A recent study has characterized the antennal expression of sensory-receptor coding genes (chemoreceptors - ORs-IRs, transient receptor potential channels - TRPs, and pickpocket receptors - PPKs) and others related to sensory function (odorant binding proteins - OBPs and chemosensory proteins - CSPs) in *R. prolixus* [17].

Insect behaviour in response to relevant external stimuli can be modulated at peripheral and central levels, depending on age, physiological status (i.e., phase of daily cycle, nutritional or reproductive status) and experience [18]. For instance, starved mature kissing-bugs seek host cues promptly, but bugs do not express proper host-seeking behaviour during the first week after ecdysis [19] or after engorgement [8]. Electroantennography and single sensillum recordings performed on different insect species have reported a high degree of physiological plasticity at sensory levels [20, 21], at least partially explaining behavioural changes triggered by feeding or development. Similar changes have been documented at the molecular level, with altered gene expression associated with feeding [22] or age [23]. In fact, variations in gene expression depending on nutritional status or development have been described for olfactory co-receptors in the antennae of *R. prolixus* [11]. Nevertheless, information about elements regulating sensory gene transcription and the abundance of the corresponding proteins in insect peripheral organs is very limited [24–27]. Physiological mechanisms modulating peripheral responses to sensory stimuli involve signalling controlled by biogenic amines, hormones, and neuropeptides, as well as their target G protein-coupled receptors (GPCRs) and nuclear receptors, overall controlling the functional status of sensory processes [18]. In this sense, the main objective of this study is to characterize modulatory components potentially involved in the local regulation of antennal sensory function using *R. prolixus* as a model insect and RNA-Seq. Furthermore, we looked at gene expression in the antennae of 5^th^ instar larvae and, male and female adults to characterize changes in this regulatory gene repertoire potentially associated with imaginal moult and sex.

## Results

Read sequences (available on PRJNA281760 project at NCBI) and de novo assemblies were obtained from Latorre-Estivalis et al. [17]. In that study, three antennal transcriptomes from 5^th^ instar larvae, and female and male adults from *R. prolixus* were sequenced using 100 base pairs (bp) paired-end reads on an Illumina HiSeq 2000 system at the W. M. Keck Centre for Comparative and Functional Genomics (University of Illinois at Urbana-Champaign, IL, USA). Sequencing outputs were 51.5 M; 62.5 M; and 62.8 M raw reads for larval, female and male libraries, respectively.

### Manual gene curation, re-annotation and identification of new genes

#### Neuropeptide and neurohormone precursor genes

A total of 15 neuropeptide precursor gene models that were absent from the RproC3.3 version of the *R. prolixus* genome were included in a new Generic Feature Format (GFF) file (Additional file 1: Table S1). The long neuropeptide F (*NPF*) and orcokinin (*OK*) predictions were corrected according to Sedra and Lange [28] and Sterkel et al. [29], respectively. The RYamide (*RYa*) gene model was fixed based on our antennal transcriptomes. Besides, IDLSRF-like peptide, glycoprotein hormones alpha-2 (*GPA2*) and beta-5 (*GPB5*), and bursicon-beta (also known as the partner of bursicon- *Burs*) genes were identified in the *R. prolixus* genome. A new isoform of the *R. prolixus* adipokinetic hormone (*AKH*) precursor gene, originated through alternative splicing, was identified in the antennal assemblies. Both AKH isoforms share the signal peptide and the active conserved peptide, but differ in the C-terminal region. Whereas the previously reported isoform encodes the core peptide and a single spacer peptide, the isoform presented here encodes the core peptide and two non-conserved spacer peptides. The gene models of eclosion hormone (*EH*); ion transport peptide (*ITP*) isoform A, *NVP-like*, *OK-B*, and *OK-C* remained incomplete because it was impossible to fix them due to problems in the genome assembly, i.e. some fragments were located in the opposite strand or were absent from the genome assembly.

#### GPCRs

Most of the biogenic amine-related GPCR gene models were edited (Additional file 2: Table S2). However, many of these genes models are still incomplete. In the case of Family A neuropeptide receptor genes, a total of 15 gene models based on Ons et al. [30] were included in the GFF file of the *R. prolixus* genome. Besides, 11 gene models of this receptor family were edited in the existing GFF file of the genome. Two isoforms (α and β) of the corazonin receptor (*CZ-R*) gene were described by Hamoudi et al. [31]. Nevertheless, our antennal transcriptome only presented the α-isoform (GenBank Acc. N° AND99324). A second kinin receptor (*K-R*) (previously described as an orphan receptor by Ons et al. [30]) and a tachykinin receptor 86C-like (*TK-R 86C-like*) were identified. Most of the Family B neuropeptide receptor gene models were fixed and included in the modified version of the *R. prolixus* GFF file (details in Additional file 2: Table S2).

The calcitonin-like (*CT*) and the corticotropin-releasing factor- like (*CRF*) diuretic hormone (*DH*) receptors present a high degree of sequence similarity, difficulting their identification. For this reason, we decided to build a phylogenetic tree to support their annotation (Additional file 3: Figure S1). Two *CT/DH* receptors were previously described in *R. prolixus* by Zandawala et al. [32]: receptor 1 and receptor 2, being the latter the orthologue of *Drosophila melanogaster hector* gene (FlyBase Acc. Number CG4395). Interestingly, the resulting phylogenetic tree suggested that a third *CT/DH-R* previously described by Ons et al. [30] seems to be exclusive of heteropteran insects (Additional file 3: Figure S1, clade highlighted in red). The *CRF/DH-R1* and *CRF/DH-R2* (including isoforms 2A and 2B) were grouped in a different clade as shown in Zandawala et al. [32].

#### Biogenic amine biosynthesis enzymes

All enzymes known to mediate biogenic amine biosynthesis in other insects were annotated in the last version of the *R. prolixus* genome [15]; however, minor changes would be needed to fix some of them (Additional file 4: Table S3). These models include: 1) Tyrosine 3-monooxygenase (*ple*), which synthesizes dopamine from L-tyrosine; 2) DOPA decarboxylase (*Ddc*), involved in the synthesis of dopamine from L-DOPA; 3) Tyrosine decarboxylase-2 (*Tdc2*), which participates on synthesis of tyramine from L-tyrosine and; 4) Tryptophan hydroxylase (*Trh*), which synthesizes serotonin from L-tryptophan.

#### Neuropeptide processing enzymes

The neuropeptide processing enzymes were not previously annotated in the *R. prolixus* genome [15]. Using sequences from *D. melanogaster* neuropeptide processing enzymes as queries, we were able to identify a total of 9 putative orthologues in *R. prolixus* (Additional file 5: Table S4). The processing of neuropeptides involves the following enzymes: 1) signal peptidase (*SP*), which cleaves the signal peptide from the N-terminal of the precursors; 2) three members of the furin subfamily (*dFUR1*, *dFUR2a* and *dFUR2b*), which are subtilisin-like endoproteases that cleave the propeptide at monobasic (Arg) and dibasic (Arg-Arg/Lys-Arg) sites; 3) prohormone convertase 2 (*amontillado* or *PC2*), which cleaves mono (Arg) and dibasic (Arg-Arg; Lys-Arg; Arg-Lys; Lys-Lys) sites; 4) the carboxypeptidase M - *CPM* (two new isoforms were identified in the antennal assemblies with differences in the 3′ region) and D (known as *silver*, which trims C-terminal Arg and Lys after Furins/PC2 cleavage reaction); 5) the *PHM* (Peptidylglycine alfa-hydroxylating mono-oxygenase) amidating enzyme, which is responsible for the alpha-amidation of the peptide C-terminal; 6) a prolyl endoprotease belonging to the peptidase 9 protein family, for which no functional information is available in insects (Additional file 5: Table S4); and 7) the amidating enzymes, the peptidyl alfa-hydroxyglycine alfa-amidating lyases (*PAL*) 1 and 2.

#### Nuclear receptors

To date, the ecdysone receptor (*EcR*) gene was the only nuclear receptor annotated in the *R. prolixus* genome [15]. Based on the characterization reported for *D. melanogaster* [33], two isoforms were expected for the ecdysone- induced protein 75B ( *Eip75B*) gene. The corresponding sequence of our assembly was identified as isoform B (with the first exon located in the second intron of isoform A), while the one deposited in VectorBase was annotated as isoform A (with two distinctive exons in the N-terminal region). Based on our results, and considering phylogenetic relations with the nuclear receptors from *Cimex lectularius*, *Pediculus humanus*, and *D. melanogaster* (Additional file 7: Figure S2), we have annotated 20 additional nuclear receptor genes in the *R. prolixus* genome (Additional file 6: Table S5). The orthologs of *knirps* (*kni*) and hormone receptor-like 83 (*HR83*) genes were not identified either in the *R. prolixus, C. lectularius* or *P. humanus* genomes, while the gene named *eagle* (*ea*) is absent in the genomes of *R. prolixus* and *C. lectularius.* Interestingly, two paralogs of the hepatocyte nuclear factor 4 ( *HNF4*) gene (A and B) were found in the *R. prolixus* genome.

#### *takeout* genes

Three *takeout* (*to*) genes had been previously identified in the *R. prolixus* genome: *Rproto1* (RPRC010098); *Rproto2* (RPRC002313); and *Rproto3* (RPRC01009) [15]. A total of 12 new *to* gene sequences were identified in our assemblies (Additional file 8: Table S6). These genes were annotated based on their phylogenetic relations (Fig. 4). Considering this analysis, RPRC002313 and RPRC010096 were annotated as *Rproto6* and *Rproto2*, respectively. *R. prolixus to* genes were separated into two different clades: *to1*-*to9* and *to10*-*to15*. All the structural characteristics of *to* genes were identified in *R. prolixus to* sequences: the presence of signal peptide; two conserved cysteine residues in the N-terminal region and two conserved characteristic motifs [34]. As expected, the length of all *to* sequences was close to 250 amino acids (Additional file 9: Figure S3). Finally, it was observed that 11 out of 15 *to* genes clustered in KQ034137 and KQ034102 supercontigs, with 8 and 3 genes, respectively (Additional file 10: Figure S4). The latter suggest that *to* genes could be products of gene duplication events.

### Antennal expression profiles

Diverse regulatory genes showed significantly different expression levels between larval vs. female or larval vs. male antennae (Additional file 12: Table S7). Nevertheless, no significant differences in expression levels were found for the regulatory genes studied here between male and female antennae. Data from our RNA-Seq experiment were used to previously report changes in the expression of genes with sensory function, e.g., ORs [17].

#### Neuropeptide and neurohormone precursor genes

**Fig. 1:**
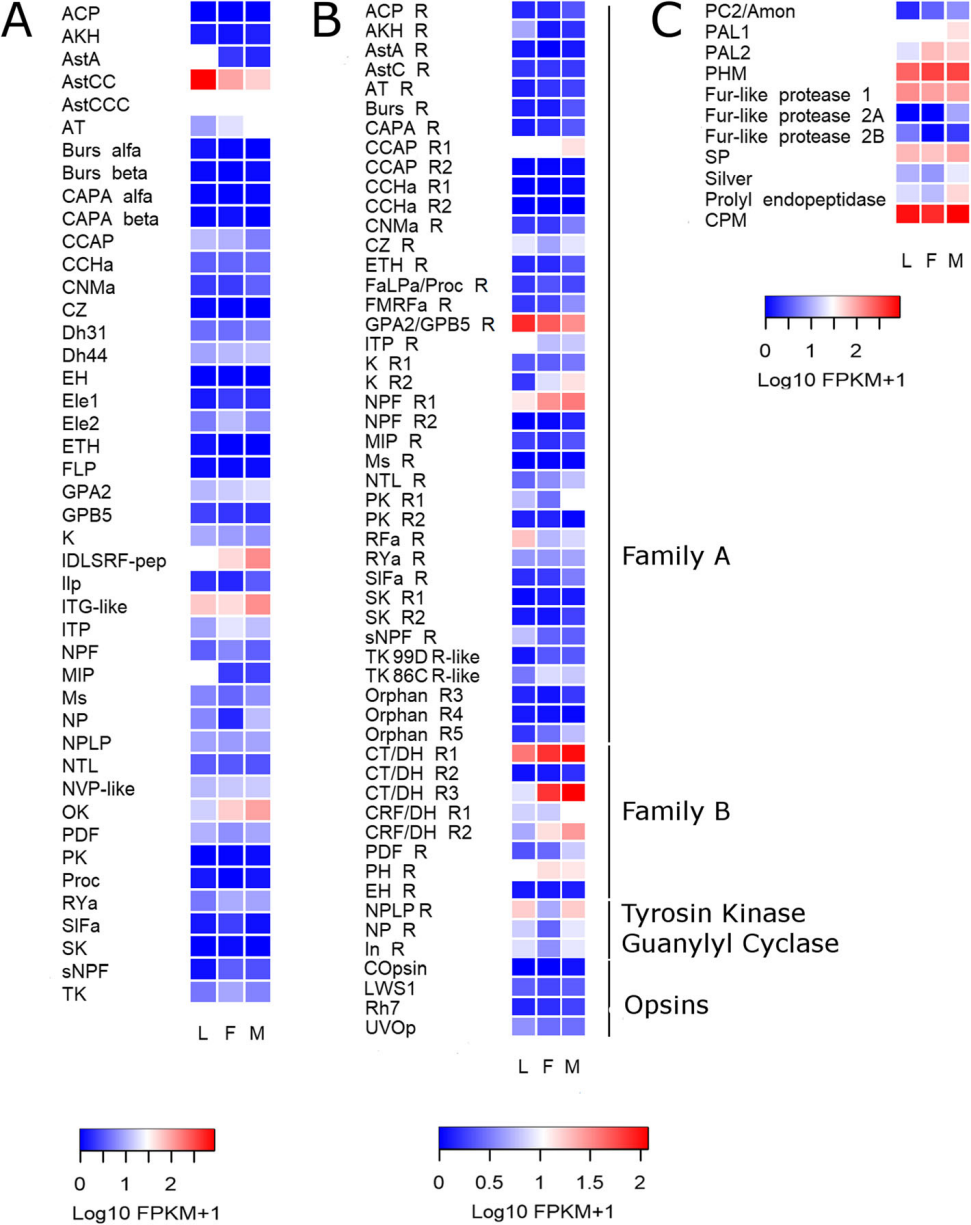
Heat map comparing the expression levels of (**a**) neuropeptide precursor genes, (**b**) G protein-coupled receptor genes, and (**c**) neuropeptide processing enzymes in the antennae of *R. prolixus* larvae (L), female (F) and male (M) adults. Expression levels (displayed as Log10 FPKM + 1) represented by means of a colour scale, in which blue/red represent lowest/highest expression. The complete names of neuropeptide precursor genes, their receptors and enzymes are detailed in Additional files 1, 2 and 5

Out of the 44 neuropeptide precursor genes annotated in the *R. prolixus* genome, 31 were found to be expressed in antennae if a criterion of > 1 Fragment *Per* Kilobase of exon model per Million reads mapped (FPKM) in at least one library was considered as an exclusion threshold (see Additional file 11: Data file S1). Fifteen out of 44 *R. prolixus* neuropeptide genes showed FPKM values higher than 10 in at least one library. Allatostatin-CC (*AstCC*), allatostatin-CCC (*AstCCC*), *ITG-like*, IDLSRF-like peptide and *OK* were the most highly expressed neuropeptide genes in the antennae of *R. prolixus* (Fig. 1a and Additional file 11: Data file S1). The gene encoding for *AstCC* was the one showing the highest expression in our database, especially in larval antennae (larval FPKM value = 888; female FPKM value = 98.5 and male FPKM value = 55). Indeed, the lower expression of this gene in male antennae was statistically significant (False Discovery Rate, FDR < 0.05) when compared to that observed in larval antennae (Additional file 12: Table S7). For *AstA* and myoinhibitory peptide (*MIP*), a significantly lower expression (FDR < 0.05) was also observed in the antennae of both adult stages when compared to larvae. The antennal expression of allatotropin (*AT*), *OK* and IDLSRF-like peptide seems to increase after imaginal moult (Fig. 1a). As we are interested on reporting gene expression levels rather than those of isoforms, the expression reported for *OK*, diuretic hormone 31 (*Dh31*), *CAPA*, *AKH* and *ITP* is the sum of those of their different isoforms or splicing variants.

#### GPCRs

The majority of Family A neuropeptide receptor genes (25 out of 38) were expressed in bug antennae (FPKM values > 1 in at least one library; Additional file 11: Data file S1). Crustacean cardioactive peptide receptor 1 (*CCAP-R1*), *NPF* receptor 1 (*NPF-R1*), *ITP* receptor (*ITP-R*), *GPA2/GPB5* receptor (*GPA2/GPB5-R)* and RFamide peptide receptor (*RFa-R*) were the most highly expressed Family A receptor-coding genes (Fig. 1b and Additional file 11: Data file S1). The expression of the *AKH-R* was significantly lower (FDR < 0.05) in females, as compared to larval, antennae (Additional file 12: Table S7). Interestingly, the expression of *K* receptor 2 (*K-R2*) increased significantly in the antennae of adults (FDR < 0.05 in both sexes; Fig. 1b and Additional file 12: Table S7). The antennal expression reported for *ACP/CZ* related peptide, Capability (*CAPA*) and *CZ* receptors, as well as for pyrokinin receptor 2 (*PK-R2*) was the sum of their different isoforms.

In the case of Family B neuropeptide receptor genes, only *CT/DH-R2* showed FPKM values lower than 1 (Additional file 11: Data file S1). Five out of seven receptor genes belonging to this family presented FPKM values higher than 10 in at least one library (Additional file 11: Data file S1). *CT/DH-R3*, which according to our phylogenetic analysis seems to be exclusive of heteropterans, showed the highest expression for this family. In fact, its expression showed a significant increase in the antennae of males (FDR < 0.05) when compared to those from larvae (Fig. 1b and Additional file 12: Table S7). The expression patterns observed for *CT/DH-R1* (isoforms B and C included) and *CRF/DH-R2* (isoforms A and B included) genes did not present an equivalent significant increase, although showing a similar tendencies (Fig. 1b). Regarding opsin expression, transcripts of UV opsin and long-wave sensitive opsin 1 (*LWS1*) were detected in the three libraries (Fig. 1b and Additional file 11: Data file S1).

#### Tyrosine kinase and guanylyl cyclase type receptors

The neuropeptide-like precursor 1 putative receptor or *NPLP1*-*R* (tyrosine kinase-type) and the potential neuroparsin receptor or *NP-R* (guanylyl cyclase receptor) were found to be expressed in the antennae of *R. prolixus* (Fig. 1b).

#### Neuropeptide processing enzymes

All enzymes involved in neuropeptide processing, except prohormone convertase 1, were expressed in the antennae of *R. prolixus*, presenting values higher than 10 FPKM in at least one library (Additional file 4: Table S3). The *PHM*, *SP*, *Fur-like protease* 1 and *CPM* genes showed the highest expression (Fig. 1c).

#### Biogenic amine related genes

**Fig. 2:**
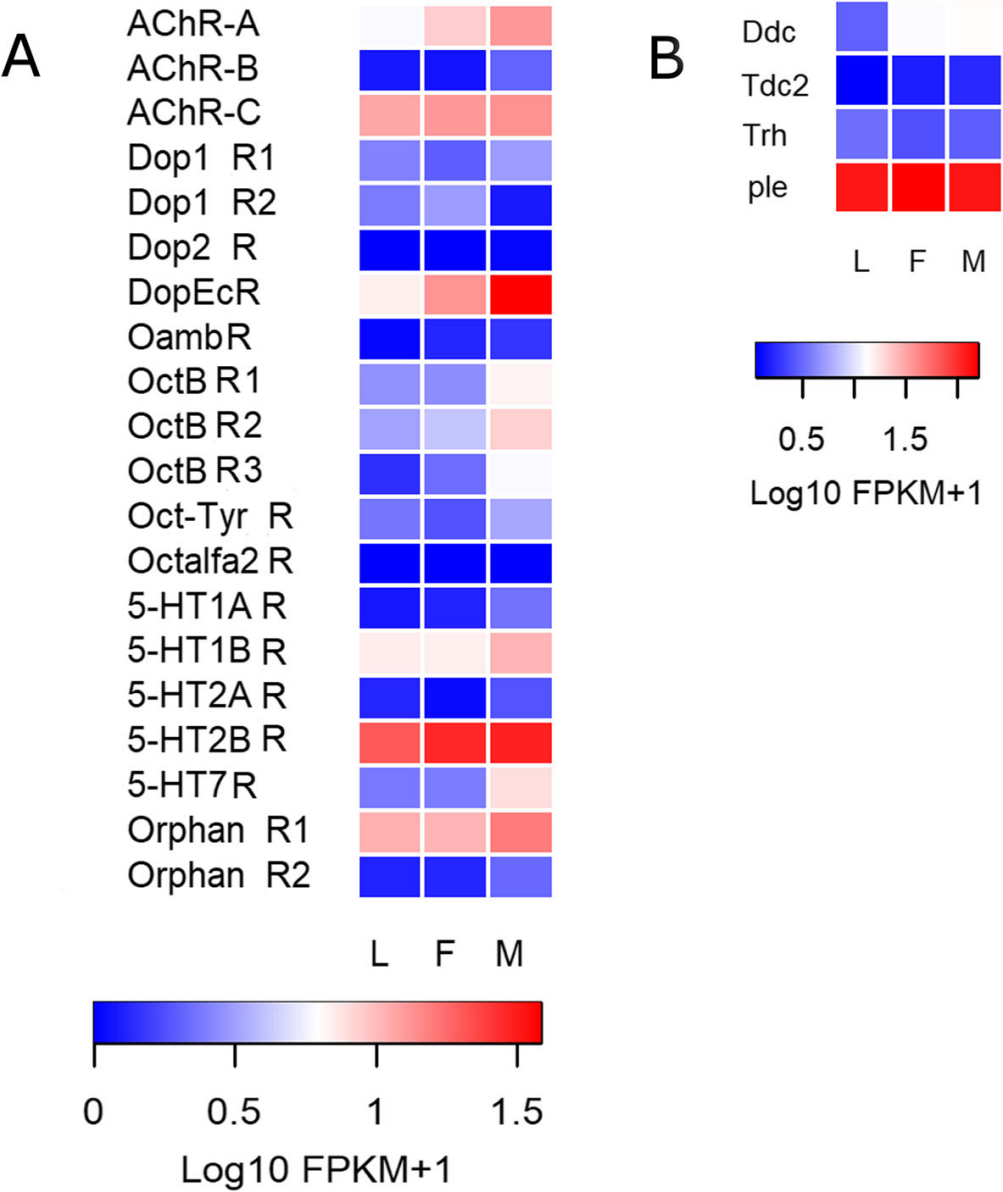
Heat map comparing antennal expression levels of *R. prolixus* genes coding for (**a**) BA-detecting GPCRs and for (**b**) enzymes involved in BA synthesis in the antennae of larvae (L), female (F) and male (M) adults. Expression levels (displayed as Log10 FPKM + 1) represented by means of a colour scale, in which blue/red represent lowest/highest expression. Complete names of biogenic amine receptors and enzymes are detailed in Additional files 2 and 4

Expression of at least 16 out of 20 biogenic amine receptor genes was detected in the antennae of *R. prolixus* (FPKM value > 1 in at least one library). Dopamine ecdysone receptor (*DopEc-R*), muscarinic acetylcholine receptor type C (*AchR-C*); orphan receptor 1; serotonin receptors 1b (*5-HT1b-R*) and 2b (*5-HT2b-R*) presented the highest antennal transcription within this group (Fig. 2a and Additional file 11: Data file S1). The expression of the octopamine beta receptor 3 (*Octβ-R3*) showed a significant increase (FDR < 0.05) in male antennae compared to larvae (Additional file 12: Table S7), while *Octβ-R1* and *Octβ-R2* presented no significant differences, even though showing a similar trend (Fig. 2a).

All genes encoding for enzymes involved in the biosynthetic pathway of biogenic amines were detected in the antennae of *R. prolixus* (Fig. 2b). The *ple* gene, which synthesizes DOPA from L-tyrosine, was the most highly expressed of this group (Fig. 2b).

#### Nuclear receptor genes

**Fig. 3:**
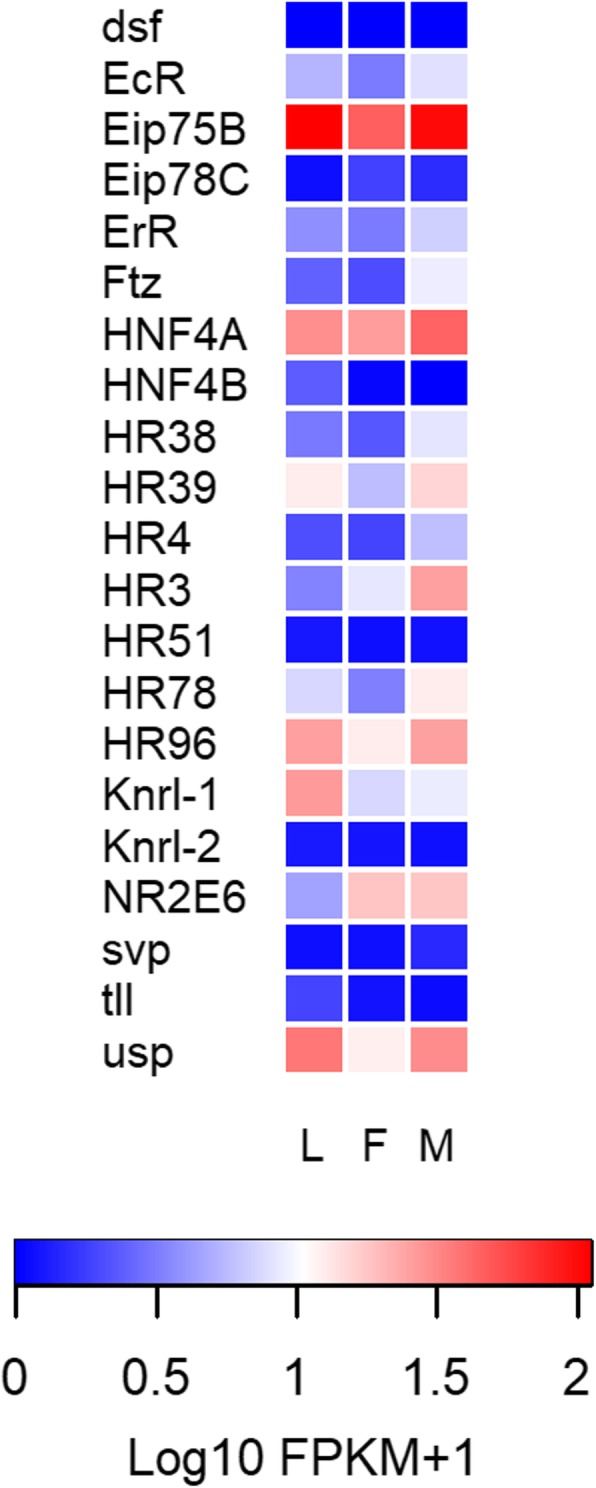
Heat map comparing the expression levels of *R. prolixus* nuclear receptor genes in the antennae of larvae (L), female (F) and male (M) adults. Expression levels (displayed as Log10 FPKM + 1) represented by means of a colour scale, in which blue/red represent lowest/highest expression. Complete names of these genes are detailed in Additional file 6

*Eip75B*, *HNF4A*, *HR96* and *ultraspiracle* (*usp*) were the genes with the highest expression, with FPKM values > 10 in the three libraries (Fig. 3; Additional file 11: Data file S1). The expression of *HR3* increased significantly after imaginal moult in male antennae (FDR < 0.05). Conversely, *HNF4B* expression was significantly lower in adult than in larval antennae (FDR < 0.05 in both sexes; Additional file 12: Table S7). Six nuclear receptor genes had no expression (FPKM value < 1 in the three libraries) in the *R. prolixus* antennal transcriptomes, these were: *dissatisfaction (dsf)*, ecdysone-induced protein 78C (*Eip78C*), *HR51*, *knirps-related 2 (knrl2)*, *tailless* (*tll*) and *seven-up* (*svp*) (Fig. 3; Additional file 11: Data file S1).

#### *takeout* genes

**Fig. 4:**
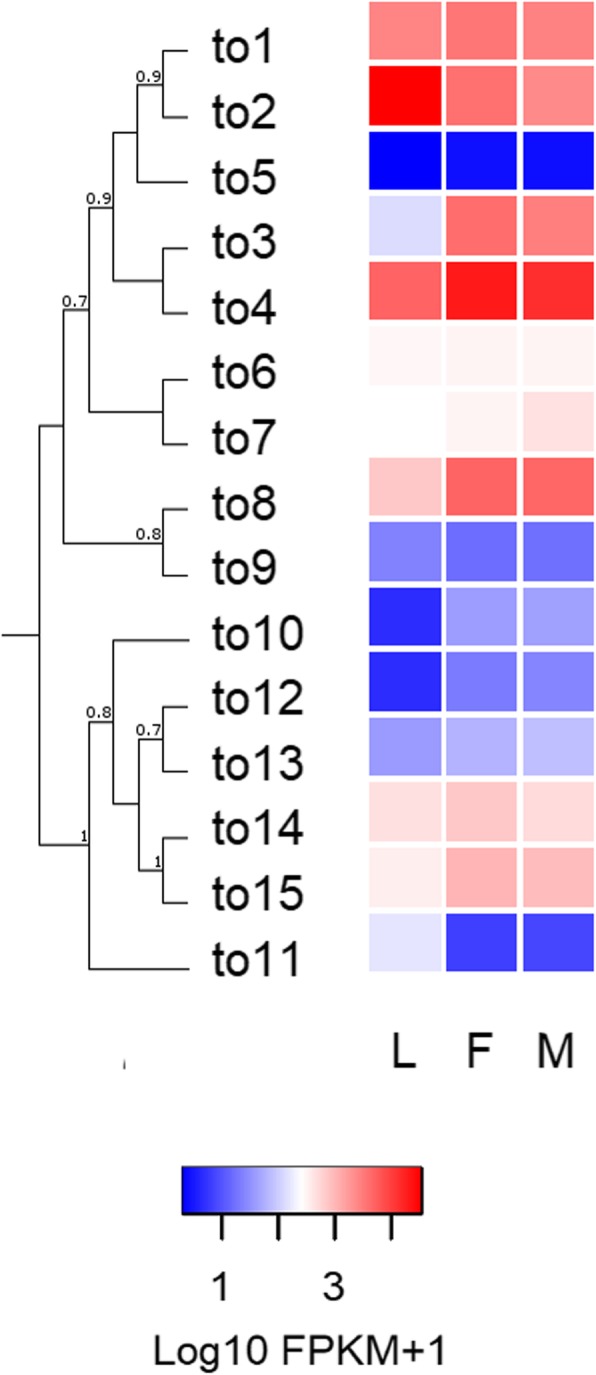
Heat map comparing the expression levels of *R. prolixus to* genes in the antennae of larvae (L), female (F) and male (M) adults. Expression levels (displayed as Log10 FPKM + 1) represented by means of a colour scale, in which blue/red represent lowest/highest expression. The evolutionary history of *R.* *prolixus takeout* genes was inferred by using the maximum likelihood method in PhyML v3.0. The support values on the bipartitions correspond to SH-like *P* values, which were calculated by means of the aLRT SH-like test. The LG substitution amino-acid model was used

These genes were highly expressed in *R. prolixus* antennae (Fig. 4); 6 out 15 presenting FPKM values higher than 1,000 in at least one library (Additional file 11: Data file S1). Two *to* genes showed significantly decreased expression in adult antennae. In the first place, *Rproto11* gene showed a significant decrease after imaginal moult (FDR < 0.05 in both sexes; Additional file 12: Table S7), while the expression of *Rproto2* decreased significantly only for male adults (FDR < 0.05). On the other hand, the expression of *Rproto3* showed a significant increase in both sexes after imaginal moult (FDR < 0.05).

## Discussion

The molecular bases of sensory plasticity at the local antennal level have been sparsely analysed (revised by [18]). Our study has characterized the expression profile of a diverse set of genes encoding different modulatory elements (neuropeptides, GPCRs, nuclear receptors and *to* genes) in the antenna of *R. prolixus*. The antennal transcription of a broad repertoire of these genes suggests that diverse local systems may be dedicated to the modulation of antennal function, such as the detection of host cues and communication signals [1].

Our results have proven that neuropeptide gene transcripts are produced in the antennae of kissing-bugs (a total of 31 neuropeptide genes were found to be expressed). The expression of neuropeptide gene transcripts has already been reported in the antennae of a few insect species [25, 35, 36]. The expression of neuropeptide processing enzyme genes was also detected in bug antennae. As far as we know, this is the first report on the expression of this type of enzyme-coding genes in insect antennae (Fig. 1c). The results presented herein add evidence supporting the antennal production of neuropeptides. However, immunohistochemistry experiments would be necessary to identify the types of cells producing neuropeptide transcripts in insect antennae. In this sense, the presence of neurosecretory cells in insect antennae has only been described for mosquitoes [37]. The authors showed that these cells form synaptoid sites on the dendrites of sensory neurons [37, 38].

In recent years, modulatory action by different neuropeptides has been shown for both antennal and labellar chemosensory neurons [24–27]. Nevertheless, the source of these neuropeptides, whether local or central, was not reported. The current study shows that *R. prolixus* antennae produce a diversity of neuropeptide-coding transcripts, among them high levels of AstCC and ITG-like peptide transcripts in the three libraries (Fig. 1a). Functional RNAi or CRISPR/CAS9 studies should be performed in order to elucidate their role. *OK* and IDLSRF-like peptide presented increased antennal expression after the imaginal moult, suggesting that these peptides may modulate adult-specific sensory processes underlying dispersion by flight and mating in kissing-bugs. On the other hand, the decreased antennal expression of *AstA* and *MIP* in adults, when compared to 5^th^ instar larvae, suggests an augmented role in immature instars. The significantly lower expression of *AstCC* in male antennae may suggest a sex-specific antennal role.

The expression of 33 out of 49 genes coding for neuropeptide and neurohormone receptors (FPKM value > 1, Additional file 11: Data file S1), the other fundamental component of the neuropeptidergic system, suggests that diverse local regulatory processes can react to a similarly complex set of modulatory signals. Indeed, 14 neuropeptides/neurohormones and their corresponding receptors presented expression higher than 1 FPKM in at least two conditions (Table 1), reinforcing that parallel local regulatory systems may modulate diverse components of antennal sensory function. The expression of neuropeptide receptor genes in antennae has been already described in other insects [35, 36]. The high expression shown in all conditions by *NPF-R1*, *GPA2/GPB5-R* (also known as leucine-rich repeat-containing GPCR1 - *LGR1*) and *CT/DH-R1* (Fig. 1b), suggests important regulatory roles on antennal function for them. Interestingly, a NPF-based system modulates responsiveness to food odours of a specific class of olfactory sensory neuron (OSN) in *D. melanogaster* [27]. The significantly augmented expression of *K-R2* observed in the antennae of adults (Fig. 1b and Additional file 12: Table S7) suggests a regulatory function of adult-specific sensory processes. A similar increased adult expression profile was previously observed in the antennae of *R. prolixus* for several chemoreceptors [17]. The significant increase detected for *CT/DH-R3* in male antennae when compared to those of larvae may suggest a sexually dimorphic role for this receptor. Therefore, it would be interesting to study whether these are functionally connected in the adult phase. The significant decrease observed in the expression of the *AKH-R* gene in female antennae may suggest a relation to the modulation of pheromone perception and production as it was observed for *D. melanogaster* in a sex-specific and starvation dependent manner [39]. It would be interesting to analyse its functional role in the antennae of kissing-bugs, for which the *AKH-R* gene had its function characterized in other tissues [40–42].

**Table 1:** Antennal expression of neuropeptides and their corresponding receptors with FPKM values higher than 1 in at least two of the analysed conditions. Complete names are detailed in Additional file 1: Table S1 and Additional file 2: Table S2

Neuropeptide	Larvae	Female	Male	Receptor	Larvae	Female	Male
*CCAP*	11.3	9.8	4.5	*CCAP-R*	12.1	9.8	14.1
*Dh31*	3.4	3.3	4.8	*CT/DH-R1*	41.8	81.1	116.7
				*CT/DH-R3*	7.5	79.3	131.6
*Dh44*	7.7	10.7	12.4	*CRF/DH-R1*	6.5	6.1	10.9
				*CRF/DH-R2*	4.1	14.8	29.2
*GPA2*	10.5	14	17.5	*GPA2/GPB5-R*	87.9	54.1	32.5
*GPB5*	1.37	1.06	1.02				
*LK*	8.7	7.2	6	*K-R1*	1.3	1.5	2.2
				*K-R2*	0.7	7.4	14.1
*ITP*	7.5	20.1	12	*ITP-R*	11.3	5.2	5.9
*NPF*	2.5	5.1	2.6	*NPF-R1*	13.5	31.8	39.8
*Ntl*	2.4	2.3	2.1	*Ntl-R*	1.7	2.9	5.4
*NP*	5.1	0.7	11.5	*NP-R*	6.2	1.6	8.2
*NPLP1*	7.7	7.1	7.9	*NPLP-R*	1.3	0.7	1.3
*PDF*	9.6	5.8	8.1	*PDF-R*	1.2	1.7	6.1
*RYa*	3.9	8.9	8	*RYa-R*	3.25	3.2	3.87
*sNPF*	0.2	2.5	1.9	*sNPF-R*	5.3	1.6	1.5
*TK*	3.8	8.1	4.8	*TK R 86C-like*	2.1	7.2	5.8
				*TK R 99D-like*	0.2	1.3	1.4

Peripheral effects of biogenic amines and their antennal production in insects have been reviewed by [43]. As observed for neuropeptides [25, 27], the modulation of chemosensation and other sensory modalities by biogenic amines [44, 45] depends on their levels [43], as well as the abundance of their receptors [46]. Actually, in situ hybridization allowed detecting octopamine and tyramine receptor gene transcripts in the vicinity of sensory receptor neurons of different insects [22, 47]. Furthermore, the presence of the *DopEc-R* has been shown for the labellar cells expressing Gr5 in *D. melanogaster* [45]. This supports the existence of direct modulatory effects of biogenic amines on peripheral sensory neurons. Biogenic amines such as octopamine have been proposed to directly affect signal transduction and spike generation on OSNs [48]. Consistent with these findings, a diverse set of transcripts of biogenic amine receptors was identified in the antennal transcriptome of *R. prolixus* (a total of 16 biogenic amine receptor genes seem to be expressed in them) and in those from other insects [24, 35, 36]. As observed for neuropeptides, most of the genes coding for enzymes involved in the biosynthesis of biogenic amines seem to be expressed in *R. prolixus* antennae (Fig. 2b). The high expression observed for 5-HT receptors in *R. prolixus* (Fig. 2a) seems to suggest the existence of antennal serotonergic nerve fibres, as described for mosquitoes [49]. The *DopEc-R*, which binds dopamine and ecdysone, showed a high expression on adult antennae, especially in those from males (Fig. 2a). Interestingly, this receptor modulates sex pheromone sensitivity in the antennal lobe of male moths [50]. Our results suggest a similar modulation could also occur at the peripheral level in *R. prolixus* male antennae. Octopamine receptors may also have a modulatory role on male sensory processes, as they showed increased expression, this being significant in the case of β-receptor 3, in the antennae of male adults (Fig. 2a; Additional file 12: Table S7). A role of octopamine receptors in the modulation of male sensory physiology was observed in male moths in which this molecule enhances OSN sensitivity to specific sexual pheromone components [48].

Hormonal regulation on insect sensory systems has been poorly studied at the peripheral organs [51]. Here we show that most insect nuclear receptors are expressed in the antennae of an insect (Fig. 3 and Additional file 11: Data file S1), suggesting that these organs have a broad capacity to respond to endocrine signals. It is worth mentioning that *Eip75B* and *HNF4A* are the most highly expressed nuclear receptor genes in *R. prolixus* antennae (Fig. 3). Considering ecdysteroid signalling, the detection of *Eip75B* transcripts indicates a potential capacity of kissing-bug antennae to respond to the *EcR-usp* complex (Ecdysone receptor + *ultraspiracle*), as observed for *Spodoptera litoralis* [51]. Besides, Eip75B and HR51 transcripts (also known as *unfulfilled*) have been identified in central clock cells of *D. melanogaster* and control the expression of clock genes, playing an important role in the maintenance of locomotor rhythms [52, 53]. Therefore, we suggest that these nuclear receptors may have a similar regulatory role at the periphery, considering that the presence of a peripheral circadian clock has been reported for insect antennae [54]. Lack of *HNF4A* expression induces decreased transcription of genes coding for enzymes driving lipid mobilization and β-oxidation in *D. melanogaster* [55]. Furthermore, *HNF4A* is required for fly starvation to induce increased transcription of these genes [55]. Interestingly, the high expression of *HNF4A* seen in *R. prolixus* antennae may relate to the relatively low nutritional status of the insects used in our studies. The increased male expression of *HR3*, which is the heterodimer partner of *Eip75B*, suggests a sex-specific role in antennae. Vidal et al. [56] reported 19 nuclear hormone receptor genes in the genome of *R. prolixus* that belong to the NR1-NR6 subfamilies. These genes were all annotated by the present study that also allowed identifying a duplication of the *HNF4A* gene. As in Naggan et al. [57], we have also found two *knrl* genes of the NR0 subfamily in the *R. prolixus* genome. Furthermore, we confirm here that *HR83* is not present in the genomes of *R. prolixus*, *C. lectularius* and *P. humanus*.

Fifteen *to* genes were identified in the *R. prolixus* genome, while Ribeiro et al. [58] identified 18 potential to transcripts in a midgut transcriptome of this species and Marchant et al. [59] identified 25 to transcripts in the transcriptome of the kissing-bug *Triatoma brasiliensis*. Consistently, these numbers match the scale of those found in *Anopheles gambiae* (10), *Acyrthosiphon pisum* (17), and *Bombyx mori* (14) genomes [60]. *R. prolixus to* genes present a cluster organization (Additional file 10: Figure S4), probably due to gene duplication events, as it was previously observed in other insects [60]. The antennal expression of *to* genes has already been reported in Dipterans [61, 62]. Furthermore, it has been shown that starvation induces the expression of these genes [62] that have also been related to foraging activity [63]. This putative function could explain the high expression observed in the three antennal libraries (Fig. 4), however, functional studies need to be performed to be able to confirm these roles in the antennae of kissing-bugs. Two *to* genes presented significant differences between larval and adult antennal transcriptomes (*Rproto11* and *Rproto3,* with an up and downregulation, respectively) and *Rproto2* is significantly down-regulated when male antennae are compared to those of larvae (Additional file 12: Table S7). Results suggest that these *to* genes may be related to sex, as observed in *D. melanogaster* [64].

Diverse antennal cells are bathed by haemolymph carrying physiological signals from central origin, but not the dendrites of sensory neurons (which are bathed by sensillar lymph).

Therefore, it is certain that central signals, i.e., circulating hormones, biogenic amines and neuropeptides, can modulate the function of most cells in insect antennae. 

However, the antennal detection of neuropeptide transcripts (and those of enzymes involved in their biosynthesis and that of biogenic amines) suggests the existence of local regulatory systems that could represent additional sources of modulation of the sensitivity of peripheral neurons.

## Conclusions

Bug antennae present consistent expression of a diverse array of components of modulatory processes, such as neuropeptides, GPCRs and nuclear receptors. Several of these components present changes in expression levels when antennae of larvae are compared to those of adults, suggesting a potential involvement in modulating physiological changes related to adult sensory needs. Future RNA-Seq, peptidomics, in situ hybridisation and functional genetics experiments should test whether these regulatory components are also present in the antennae of other insects and unveil the interaction between central and peripheral modulatory systems to understand their relative contribution to the regulation of antennal sensory physiology.

## Methods

### Transcriptomic data analysis

Read sequences and de novo assemblies were obtained from JM Latorre-Estivalis, HM Robertson, KK Walden, J Ruiz, LO Gonçalves, A Guarneri and MG Lorenzo [17]. The cited study reported three separate transcriptomes from antennae of 5^th^ instar larvae, female and male *R. prolixus* adults (colony originated from Honduras and held at the Instituto René Rachou - FIOCRUZ). Fifth instar larvae, female and male adults were 21 day-old and unfed at the time of antennal excision. Based on a broad array of reports on triatomine behaviour [4, 65, 66], antennae used belonged to sensory mature insects in all cases. Furthermore, by comparing the transcriptomes of the last sexually immature instar and those of adults, the dataset allowed detecting changes in gene expression profiles associated with the imaginal moult. A total of 60 antennae were collected *per* sample and used for RNA extraction for subsequent RNA-Seq library preparation and sequencing, as described above. FASTQC was used to assess read quality and detect Illumina adapters. Subsequently, FASTX Toolkit (http://hannonlab.cshl.edu/fastx_toolkit/index.html) was used to trim off biased reads at the 5′ end, and then using the quality score trimmer to remove low-quality reads at the 3′ end of the read (− t 20). After the trimming process, the final number of paired-end reads was 51.2 M; 62.2 M; and 62.4 M for larvae, female and male libraries, respectively. All trimmed reads were used to elaborate two de novo transcriptome assemblies by means of SOAPdenovo v.1.0242 and Trinity (release 2012-03-17) packages. Trimmed reads were mapped to the *R. prolixus* genome assembly (version RproC3.3) and an edited GFF file by means of STAR v.2.6.0 [67] with default parameters. The number of mapped reads was 48.3 M; 46.5 M; and 37.7 M for larval, female and male libraries, respectively. Read counts were obtained using the *--multicov* command from BEDtools v.2.29.1.

### Differential expression analysis

Comparisons among stages and between sexes were performed using the edgeR (v3.6.8) package. First, we eliminated genes with low expression (only those presenting > 1 count-per-million in at least one library were kept) and performed the normalization step using the *calcNormFactors* command. Afterwards, we followed the edgeR User’s Guide recommendations for experiments without replicates (see 2.11 section at page 23). We set the common dispersion at 0.2 (corresponding to a coefficient of biological variation of 0.45) and read counts were analysed using the likelihood ratio test within at GLM approach. Expression data were fit to the model (using *glmFit*) and differentially expressed genes were identified by means of the *glmLRT* and *topTags* functions. A FDR adjusted *p*-value < 0.05 was set as threshold to define the significance level. Heat maps showing gene expression (expressed as Log10 FPKM value + 1) of the different protein families in the conditions tested were prepared using the gplot package in R.

### Manual gene curation

Manual curation of genome project databases by means of the inclusion and correction of gene models, using transcriptomic data and published studies, is fundamental for increasing database quality. The use of reliable genome databases, which need to be as complete and validated as possible, is especially relevant for performing adequate quantitative transcriptomic and functional genetic studies. Most of the target sequences curated herein were obtained from previous reports [15, 30, 68–70] (details in Additional file 1: Table S1 and Additional file 2: Table S2). Therefore, all sequences were compared to the SOAPdenovo and Trinity generated antennal assemblies from JM Latorre-Estivalis et al. [17]. The discrepancies observed between target gene models from the *R. prolixus* genome (Gene set: RproC3.3, available on 24 Oct 2017) and the transcripts from the de novo antennal assemblies are reported in Additional files 1, 2, 4, 5, 6 and 8. In the case of neuropeptide precursor and GPCR genes that were manually corrected/extended, new GFF files were created and included in the RproC3.3 version of the *R. prolixus* genome GFF file. In the case of the other gene families, new gene models were created only for those genes that were absent from the VectorBase gene prediction database or those whose gene models were partially constructed. The modified GFF file of the genome was used for read mapping. The protein sequences of all genes analysed and the edited GFF files are included in the Additional file 13: Data file S2 and Additional file 14: Data file S3, respectively).

### Identification of new genes

Orthologous sequences from *D. melanogaster* [71, 72] were used in tBLASTn searches in the *R. prolixus* genomic database (www.vectorbase.org) to identify nuclear receptor genes and enzymes related to prepropeptide/preproprotein processing. Sequences of *to* genes previously annotated for *R. prolixus* [15] were used as queries to search for new sequences in the genome. Subsequently, all sequences were manually corrected/extended according to our de novo antennal transcriptomes and annotated based on their phylogenetic relations to other insect sequences. In addition, the structural characteristics of *to* genes, such as the presence of a signal peptide (detected by means of SignalP 4.0 [73]), of two conserved cysteine residues in the amino-terminal region implicated in disulfide bond formation and ligand binding [74], and of two conserved motifs [34] were confirmed in *R. prolixus to* sequences.

### Phylogenetic analysis

For building the phylogenetic trees, protein sequences of *R. prolixus* and other insect species were aligned using G-INS-I strategy in MAFFT v.7 (mafft.cbrc.jp/alignment/server), and manually edited in Jalview v2.6.1. Finally, maximum likelihood trees were built in PhyML v.3.0. Branch support was determined using the approximate Likelihood Ratio Test (aLRT). Non-parametric branch support was based on the Shimodaira-Hasegawa-like (SH) procedure. Fasta sequences from different insects used in the phylogenetic analyses of *CT/DH* – *CRF/DH* and nuclear receptor genes are included in Additional file 15: Data file S4.

## Supplementary information

**Additional file 1: Table S1.** Details of neuropeptide and neurohormone precursor genes. Columns are: Gene – the gene and protein name we are assigning; VectorBase code – the official gene number in the RproC3 genome assembly, prefix is RPRC; Scaffold – the RproC3.3 genome assembly supercontig ID; AAs – number of encoded amino acids in the protein; Comments – comments on the OGS gene model and repairs performed in the genome assembly based on BLAST against de novo antennal assemblies. NTE: Amino-terminal region; CTE: Carboxyl-terminal region; VB: VectorBase; GB: GenBank.

**Additional file 2: Table S2.** Details of G protein-coupled receptor genes. Columns are: Gene – the gene and protein name we are assigning; VectorBase code – the official gene number in the RproC3 genome assembly, prefix is RPRC; Scaffold – the RproC3 genome assembly supercontig ID; AAs – number of encoded amino acids in the protein; Comments – comments on the OGS gene model and repairs performed in the genome assembly (available on VectorBase) based on BLAST against de novo antennal assemblies. NTE: Amino-terminal region; CTE: Carboxyl-terminal region; VB: VectorBase; GB: GenBank.

**Additional file 3: Figure S1.** Molecular phylogenetic analyses of calcitonin diuretic (CT) and corticotropin-releasing factor-related (CRF) like diuretic hormone (DH) receptors of *R. prolixus* and other insects. The evolutionary history of *R. prolixus* CT/DH and CRF/DH receptors was inferred by using the maximum likelihood method in PhyML v3.0. The support values on the bipartitions correspond to SH-like *P* values, which were calculated by means of aLRT SH-like test. The CT/DH receptor 3 clade was highlighted in red. The CT/DH and CRF/DH *R. prolixus* receptors were displayed in blue. The LG substitution amino-acid model was used. Species abbreviations: Dmel, *Drosophila melanogaster*; Aaeg, *Aedes aegypti*; Agam, *Anopheles gambiae*; Clec, *Cimex lecturiaus*; Hhal, *Halomorpha halys;* Rpro, *Rhodnius prolixus*; Amel, *Apis mellifera*; Apis, *Acyrthosiphon pisum*; and Tcas, *Tribolium castaneum.* The glutamate receptor sequence from the * D. melanogaster* (FlyBase Acc. N° GC11144) was used as an out-group. The sequences used from other insects are reported in Additional file 15: Data file S4).

**Additional file 4: Table S3.** Details of enzymes involved in the biogenic amine synthesis. Columns are: Gene – the gene and protein name we are assigning; VectorBase code – the official gene number in the RproC3 genome assembly, prefix is RPRC; Scaffold – the RproC3 genome assembly supercontig ID; AAs – number of encoded amino acids in the protein; Comments – comments on the OGS gene model and repairs to be performed on the genome assembly (available on VectorBase) based on BLAST searches against de novo antennal transcriptome assemblies. NTE: Amino-terminal region.

**Additional file 5: Table S4.** Details of neuropeptide processing enzymes. Columns are: Gene – the gene and protein name we are assigning; VectorBase code – the official gene number in the RproC3 genome assembly, prefix is RPRC; Scaffold – the RproC3 genome assembly supercontig ID; AAs – number of encoded amino acids in the protein; Comments – comments on the OGS gene model and repairs to be performed on the genome assembly (available on VectorBase) based on BLAST searches against de novo antennal transcriptome assemblies.

**Additional file 6: Table S5.** Details of nuclear receptor genes. Columns are: Gene – the gene and protein name we are assigning; VectorBase code – the official gene number in the RproC3 genome assembly, prefix is RPRC; Scaffold – the RproC3 genome assembly supercontig ID; AAs – number of encoded amino acids in the protein; Comments – comments on the OGS gene model and repairs to be performed on the genome assembly (available on VectorBase) based on BLAST searches against de novo antennal assemblies. NTE: Amino-terminal region; CTE: Carboxyl-terminal region; VB: VectorBase.

**Additional file 7: Figure S2.** Molecular phylogenetic analysis of nuclear receptor genes of *R. prolixus* and other insects. The evolutionary history of *R. prolixus* nuclear receptors was inferred by using the maximum likelihood method in PhyML v3.0. The support values on the bipartitions correspond to SH-like *P* values, which were calculated by means of aLRT SH-like test. The *R. prolixus* nuclear receptors were displayed in blue. LG substitution amino-acid model was used. Species abbreviations: Dmel, *Drosophila melanogaster*; Phum, *Pediculus humanus*; Clec, *Cimex lectularius*. The *RproEip75B* sequence used was from isoform B (from our antennal transcriptome) because the sequence of isoform A available in VectorBase was considered incomplete. The sequences used from other insects are reported in Additional file 15: Data file S4).

**Additional file 8: Table S6.** Details of *takeout* genes. Columns are: Gene – the gene and protein name we are assigning; VectorBase code – the official gene number in the RproC3 genome assembly, prefix is RPRC; Scaffold – the RproC3 genome assembly supercontig ID; AAs – number of encoded amino acids in the protein; Comments – comments on the OGS gene model and repairs to be performed on the genome assembly (available on VectorBase) based on BLAST searches against de novo antennal transcriptome assemblies. NTE: Amino-terminal region.

**Additional file 9: Figure S3.** Alignment of *R. prolixus* takeout protein sequences. Sequences were aligned with CLUSTAL X v2.0. Asterisks indicate identical amino-acids, double points show conserved exchanges and single points show homologous amino acids. The *D. melanogaster* takeout protein sequence was obtained from Justice et al. [75]. The two conserved cysteine residues defining the takeout family [74] in many insects are marked with white boxes. The position of the conserved motifs 1 and 2 described by So et al. [34] is indicated with grey boxes. Predicted signal peptides are underlined. Species abbreviations: Rpro, *Rhodnius prolixus*; and Dmel, *Drosophila melanogaster*.

**Additional file 10: Figure S4.** Structure and organization of *takeout* gene clusters. Scaffold IDs are presented on the left. White arrows represent each *takeout* gene and its position on the scaffold.

**Additional file 11: Data file S1.** FPKM values of target genes in the three libraries.

**Additional file 12: Table S7.** Differentially expressed modulatory genes among stages studied. Comparison of normalized CPM among stages was conducted using edgeR package. CPM: count *per* million, L: larval; F: female, M: male; LogFC: Log fold change; FDR adjusted *p*-value: False Discovery Rate; n.s.: not significant.

**Additional file 13: Data file S2.** Protein sequences of all target genes in fasta format.

**Additional file 14: Data file S3.** Edited Generic Feature Format (GFF) file of the *R. prolixus* genome used for read mapping and gene expression analysis.

**Additional file 15: Data file S4.** Fasta sequences from different insects used in the phylogenetic analyses of *CT/DH* – *CRF/DH* and nuclear receptor genes.

## Data Availability

All data generated or analysed during this study are included in this published article and its supplementary information files.

## Abbreviations

aLRT: Approximate Likelihood Ratio Test
CRISPR: Clustered Regularly Interspaced Short Palindromic Repeats
CSPs: Chemosensory proteins
C-terminal: Carboxyl-terminal
FPKM: Fragment *Per* Kilobase of exon model *per* Million mapped reads
GFF: Generic Feature Format
GPCRs: G protein-coupled receptors
Gr5: Gustatory receptor 5
NR: Nuclear receptor
N-terminal: Amino-terminal
OBPs: Odorant binding proteins
OSN: Olfactory sensory neuron
PPKs: Pickpocket receptors
RNAi: RNA interference
RNA-Seq: RNA sequencing
RT-qPCR: Quantitative reverse transcription polymerase chain reaction
SH: Shimodaira-Hasegawa-like
to: Takeout
TRPs: Transient receptor potential
