# Supplementary material for: Transcriptomics supports local sensory regulation in the antenna of the kissing-bug Rhodnius prolixus
Source: BMC Genomics. 2020 Jan 30;21:101. doi: 10.1186/s12864-020-6514-3 (PMC6993403; doi:10.1186/s12864-020-6514-3)
Supplement: Supplementary file 1 — Additional file 1: Table S1. Details of neuropeptide and neurohormone precursor genes. Columns are: Gene – the gene and protein name we are assigning; VectorBase code – the official gene number in the RproC3 genome assembly, prefix is RPRC; Scaffold – the RproC3.3 genome assembly supercontig ID; AAs – number of encoded amino acids in the protein; Comments – comments on the OGS gene model and repairs performed in the genome assembly based on BLAST against de novo antennal assemblies. NTE: Amino-terminal region; CTE: Carboxyl-terminal region; VB: VectorBase; GB: GenBank. [file 12864_2020_6514_MOESM1_ESM.pdf]

**Table S1. Details of neuropeptide and neurohormone precursor genes**. Columns are: Gene – the gene and protein name we are assigning; VectorBase code – the official gene number in the RproC3 genome assembly, prefix is RPRC; Scaffold – the RproC3.3 genome assembly supercontig ID; AAs – number of encoded amino acids in the protein; Comments – comments on the OGS gene model and repairs performed in the genome assembly based on Blast searches against *de novo* antennal transcriptome assemblies. NTE: Amino terminal region; CTE: Carboxyl terminal region.

| Gene | Gene symbol | VectoBase code | Scaffold | Isoforms | AAs. | Hit against the antennal *de novo* assemblies | Comments |
|---|---|---|---|---|---|---|---|
| **Adipokinetic hormone/corazonin-related peptide** | *ACP* | - | KQ035347 | - | 126 | Yes | New gene model created based on GenBank sequence KM975505 [1] and included in the edited genome GFF |
| **Adipokinetic hormone** | *AKH* | RPRC000416 | KQ034546 | A | 71 | No | VectorBase prediction identical to GenBank sequence Acc. N° KM283242 [2] |
| | | | | B | 70 | Yes | New isoform identified based on antennal assemblies and included in the edited genome GFF |
| **AstA** | *AstA* | - | KQ034293 | - | 203 | Yes | New gene model created based on GenBank sequences Acc. N° GQ856315 and JN559385 [3] and included in the edited GFF |
| **Allatostatin B** | *MIP* | - | KQ034158 | - | 254 | Yes | New gene model created based on Ons *et al.* 2011 and included in the edited genome GFF |
| **Allatostatin CC** | *AstCC* | RPRC000300 | KQ034374 | - | 117 | Yes | No changes in VectorBase prediction |
| **Allatostatin CCC** | *AstCCC* | - | KQ034609 | - | 100 | No | New gene model was created based on Ons *et. Al.* 2011 and included in the edited GFF. Previously, it was annotated as *AstC* |
| **Allatotropin** | *AT* | - | KQ034313 | - | 119 | Yes | New gene model created was created based on GenBank sequence Acc. N° GQ162783 [3] and included in the edited genome GFF |
| **Bursicon alpha** | *Burs-alfa* | RPRC000797 | KQ034200 | - | 169 | Partial | No changes in VectorBase prediction |
| **Bursicon beta** | *Burs-beta* | - | KQ034059 | - | 107 | No | Identified in this work using *T. castaneum* GenBank sequence Acc. N° NM_001114308.1 as query. Gene model included |
| **Diuretic hormone 31** | *Dh31* | RPRC000977 | KQ034472 (5´UTR); KQ034594 (rest of the gene model) and KQ037272 (last exon) | A | 146 | Yes | Identical to GenBank sequences Acc. N° GQ856316 and AEA51300 [3]. Last 42 amino acids are located in the KQ037272 supercontig |
| | | | | B | 109 | No | Identical to GenBank sequences Acc. N° GQ856317 and AEA51301 [3] |
| | | | | C | 206 | Yes | Identical to GenBank sequence Acc. N° HM030714.1 [4] |
| **Cardioacceleratory peptide (CAPA/CAP2b)** | *CAPA* | RPRC000639 | KQ034830 | A | 158 | No | VectorBase prediction identical to GenBank sequence Acc. N° ABS17680 [5]. In VectorBase classified as non-translating CDS |
| | | RPRC000563 | KQ034830 | B | 158 | No | VectorBase prediction was identical to GenBank sequence Acc. N° ACH70295. In VectorBase classified as non-translating CDS |

| **Gene** | **Gene symbol** | **VectoBase code** | **Scaffold** | **Isoforms** | **Aas.** | **Hit against the antennal *de novo* assemblies** | **Comments** |
|---|---|---|---|---|---|---|---|
| **Crustacean cardiactive peptide** | *CCAP* | RPRC000466 | KQ034330 | - | 129 | Yes | VectorBase prediction was identical to GenBank sequence Acc. N° GQ888668 [3] |
| **CCHamide peptide** | *CCHa* | - | KQ034137 | - | 104 | Partial | New gene model created based Ons *et al*. 2011 and included in the edited genome GFF |
| **CNMamide peptide** | *CNMa* | RPRC010893 | KQ034609 | - | 150 | No | No changes in VectorBase prediction |
| **Corazonin** | *CZ* | - | KQ034239 | - | | No | New gene model created based on Ons *et al*. 2011 and included in the edited genome GFF |
| **Diuretic hormone 44** | *Dh44* | RPRC000596 | KQ034102 | - | 151 | Yes | No changes in VectorBase prediction, which is identical to GenBank sequence Acc. N° HM153808 [6], annotated as corticotropin releasing factor-like protein |
| **Eclosion hormone** | *EH* | RPRC014242 | KQ034677 | - | 241 | No | Partial sequence. Initial methionine is still missed |
| **Elevenin-1** | *Elevin-1* | RPRC003083 | KQ034317 | - | 66 | No | No changes in VectorBase prediction |
| **Elevenin-2** | *Elevin-2* | RPRC003084 | KQ034317 | - | 87 | Yes | No changes in VectorBase prediction |
| **Ecdysis triggering hormone** | *ETH* | RPRC014486 | KQ034462 | - | 146 | Yes | No changes in VectorBase prediction |
| **FLP** | *FMRFamida* | RPRC014988 | KQ035274 | - | 273 | Partial | No changes in VectorBase prediction |
| **Glycoprotein hormone alpha 2** | *GPA2* | RPRC007092 | KQ034094 | - | 122 | Partial | No changes in VectorBase prediction |
| **Glycoprotein hormone beta 5** | *GPB5* | - | KQ034094 | - | 156 | No | New gene model was created based on *C. lectularius* sequence XP_014244389.1. Methionine is missed. |
| **Kinin** | | RPRC000022 | KQ034106 | - | 398 | Yes | VectorBase prediction was identical to GenBank sequence Acc. N° BK007870 [7] |
| **IDLSRF-like peptide** | - | RPRC000351 | KQ034112 | - | 168 | Yes | No changes in VectorBase prediction |
| **Insulin-like peptide** | *Ilp* | RPRC007020 | KQ034142 | - | 126 | Yes | VectorBase prediction was identical to GenBank sequence Acc. N° AMS34841.1 [8] |
| **ITG-like** | - | - | KQ034255 | - | 214 | Yes | New gene model created based on Ons *et. al* 2011 and included in the edited genome GFF |
| **Ion transport peptide** | *ITP* | RPRC000519 | KQ034208 | A | 111 | No | Annotated as sulfakinin (GenBank sequence Acc. N° GQ2539210). Last 33 amino acids are not in the genome |
| | | | | B | 117 | Yes | VectorBase prediction was identical to GenBank sequence Acc. N° GU207866 [3] |

| Gene | Gene symbol | VectoBase code | Scaffold | Isoforms | Aas. | Hit against the antennal *de novo* assemblies | Comments |
|---|---|---|---|---|---|---|---|
| **Long Neuropeptide F** | *NPF* | RPRC008107 | KQ034255 | No | 105 | Yes | VectorBase prediction extended in NTE region and initial methionine fixed according to GenBank sequence Acc. N° KT898124.1 [9] |
| **Myosuppressin** | *Ms* | RPRC000203 | KQ034384 | No | 88 | Yes | VectorBase prediction was identical to GenBank sequence Acc. N° GQ344501 [3] |
| **Natalisin** | *NTL* | RPRC003680 | KQ034106 | - | 196 | Partial | No changes in VectorBase prediction |
| **Neuroparsin** | *NP* | RPRC002095 | Q034340 | | 113 | Yes | VectorBase prediction was identical to GenBank sequence Acc. N° GU207864 [3] |
| **Neuropeptide like precursor 1** | *NPLP1* | RPRC011668 | KQ034238 | - | 454 | Yes | VectorBase prediction was identical to GenBank sequence Acc. N° GU207865 [3] |
| **NVP-like** | *PH2* | RPRC003052 | ACPB03040762 | - | 299 | Yes | VectorBase prediction was shorter in NTE and CTE regions. Impossible to fix the genome model due to problem in the genome assembly. |
| **Orcokinin** | *OK* | RPRC014678 | KQ034149 | A | 165 | Yes | One exon added in NTE region of VectorBase prediction based on GenBank sequence Acc. N° FJ167860 |
| | | | | B | 392 | Yes | Only first 52 amino acids present in the genome. Sequence identical to GenBank sequence Acc. N° FJ761320 [10] |
| | | | | C | 422 | No | Same problem as mentioned for isoform B. Sequence identical to GenBank sequence Acc. N° KF179047 |
| **Pigment dispersing factor** | *PDF* | - | KQ034061 | - | 48 | Yes | New gene model created based on Ons *et al.* 2011 and included in the edited genome GFF |
| **Proctolin** | *Proc* | RPRC000390 | KQ034188 | - | 97 | No | VectorBase prediction was identical to GenBank sequence Acc. N° JN543225 [11] |
| **Pyrokinin** | *PK-PBAN* | - | KQ034521 | - | 122 | Yes | New gene model created based on GenBank sequence GU230851 and included in the edited genome GFF |
| **RYamide** | RYa | RPRC000461 | KQ035177 | - | 107 | Yes | Initial methionine of VectorBase model was fixed according to antennal assemblies |
| **Short Neuropeptide F** | *sNPF* | - | KQ034092 | - | 92 | Yes | New gene model created based on GenBank sequence Acc. N° GQ452380 [3] and included in the edited genome GFF |
| **SIFamide** | *SIFa* | - | KQ035590 | - | 74 | Yes | New gene model created based on GenBank sequence Acc. N° GQ253922 [3] and included in the edited genome GFF |
| **Sulphakinin** | *SK* | - | KQ034228 | - | 92 | Yes | New gene model created based on GenBank sequence Acc. N° GQ162784 [3] and included in the edited genome GFF |
| **Tachykinin** | *TK* | RPRC000843 | ACPB03026326 | - | 215 | Yes | VectorBase prediction was identical to GenBank sequence Acc. N° GQ162785 [3] |

1. Zandawala M, Haddad AS, Hamoudi Z, Orchard I: **Identification and characterization of the adipokinetic hormone/corazonin-related peptide signaling system in *Rhodnius prolixus***. *The FEBS Journal* 2015, **282**(18):3603-3617.
2. Zandawala M, Hamoudi Z, Lange AB, Orchard I: **Adipokinetic hormone signalling system in the C hagas disease vector, *Rhodnius prolixus***. *Insect Mol Biol* 2015, **24**(2):264-276.
3. Ons S, Sterkel M, Diambra L, Urlaub H, Rivera-Pomar R: **Neuropeptide precursor gene discovery in the Chagas disease vector *Rhodnius prolixus***. *Insect Mol Biol* 2011, **20**(1):29-44.
4. Zandawala M, Paluzzi J-P, Orchard I: **Isolation and characterization of the cDNA encoding DH31 in the kissing bug, *Rhodnius prolixus***. *Mol Cell Endocrinol* 2011, **331**(1):79-88.
5. Paluzzi J-P, Russell WK, Nachman RJ, Orchard I: **Isolation, cloning, and expression mapping of a gene encoding an antidiuretic hormone and other CAPA-related peptides in the disease vector, *Rhodnius prolixus***. *Endocrinology* 2008, **149**(9):4638-4646.
6. Te Brugge V, Paluzzi J-P, Schooley DA, Orchard I: **Identification of the elusive peptidergic diuretic hormone in the blood-feeding bug *Rhodnius prolixus*: a CRF-related peptide**. *J Exp Biol* 2011, **214**(3):371-381.
7. Te Brugge V, Paluzzi J-P, Neupert S, Nachman RJ, Orchard I: **Identification of kinin-related peptides in the disease vector, *Rhodnius prolixus***. *Peptides* 2011, **32**(3):469-474.
8. Defferrari MS, Orchard I, Lange AB: **Identification of the first insulin-like peptide in the disease vector *Rhodnius prolixus*: involvement in metabolic homeostasis of lipids and carbohydrates**. *Insect Biochem Mol Biol* 2016, **70**:148-159.
9. Sedra L, Lange AB: **Cloning and expression of long neuropeptide F and the role of FMRFamide-like peptides in regulating egg production in the Chagas vector, *Rhodnius prolixus***. *Peptides* 2016, **82**:1-11.
10. Sterkel M, Oliveira PL, Urlaub H, Hernandez-Martinez S, Rivera-Pomar R, Ons S: **OKB, a novel family of brain-gut neuropeptides from insects**. *Insect Biochem Mol Biol* 2012, **42**(7):466-473.
11. Orchard I, Lee DH, Da Silva R, Lange AB: **The proctolin gene and biological effects of proctolin in the blood-feeding bug, *Rhodnius prolixus***. *Front Endocrinol* 2011, **2**:59.
