# Supplementary material for: Transcriptomics supports local sensory regulation in the antenna of the kissing-bug Rhodnius prolixus
Source: BMC Genomics. 2020 Jan 30;21:101. doi: 10.1186/s12864-020-6514-3 (PMC6993403; doi:10.1186/s12864-020-6514-3)
Supplement: Supplementary file 2 — Additional file 2: Table S2. Details of G protein-coupled receptor genes. Columns are: Gene – the gene and protein name we are assigning; VectorBase code – the official gene number in the RproC3 genome assembly, prefix is RPRC; Scaffold – the RproC3 genome assembly supercontig ID; AAs – number of encoded amino acids in the protein; Comments – comments on the OGS gene model and repairs performed in the genome assembly (available on VectorBase) based on BLAST against de novo antennal assemblies. NTE: Amino-terminal region; CTE: Carboxyl-terminal region; VB: VectorBase; GB: GenBank. [file 12864_2020_6514_MOESM2_ESM.pdf]

Table S2. Details of G protein coupled receptor genes. Columns are: Gene – the gene and protein name we are assigning; VectorBase code – the official gene number in the RproC3 genome assembly, prefix is RPRC; Scaffold – the RproC3 genome assembly supercontig ID; AAs – number of encoded amino acids in the protein; Comments – comments on the OGS gene model and repairs performed in the genome assembly (available on VectorBase) based on Blast searches against *de novo* antennal transcriptome assemblies. NTE: Amino terminal region; CTE: Carboxyl terminal region.

Gene	Ligand	VectorBase code	Scaffold	AAs.	Hit against the antennal *de novo* assemblies	Comments
**BIOGENIC AMINE G PROTEIN RECEPTORS**						
**Muscarinic Acetylcholine receptor A**	Acetylcholine	-	KQ034078	555	No	VectorBase predictions (RPRC001750, 001751 and 007566) fused. The last prediction located in the opposite strand
**Muscarinic Acetylcholine receptor B**		RPRC010907	KQ034218	879	Yes	The first exon extended. Initial methionine is still missed
**Muscarinic Acetylcholine receptor C**		RPRC010656	KQ034118	315	Yes	The NTE extended. Initial methionine fixed
**Dopamine 1-like receptor 1**	Dopamine	RPRC014093	KQ034515	395	Partial	New exon added in NTE region. Initial methionine is still missed
**Dopamine 1-like receptor 2**		RPRC013708	KQ034114	426	Partial	NTE region extended. Initial methionine is still missed
**Dopamine 2-like receptor**		RPRC011175	KQ034171	99	No	Partial sequence. The last two exons eliminated.
**Dopamine Ecdysone receptor**		RPRC014528	KQ034056	354	Partial	The initial methionine is missed.<br>No changes in VectorBase prediction
**Serotonin (5-HT) receptor 1a**	Serotonin	RPRC010931	KQ034080	441	Partial	The NTE extended. The initial methionine is still missed
**5-HT receptor 1b**		RPRC008923	KQ036077 and KQ034329	578	Yes	The first 176 amino acids are located in KQ036077.<br>VectorBase prediction RPRC008923 edited
**5-HT receptor 2a**		-	KQ034057	373	Partial	VectorBase predictions RPRC005858 and RPRC001892 fused.<br>Initial methionine is still missed
**5-HT receptor 2b**		RPRC000473	KQ034057	703	Yes	Identical to GenBank sequence Acc. N° AKQ13312 [1]
**5-HT receptor 7**		-	KQ034099	423	Partial	VectorBase predictions RPRC007788 and RPRC001792 fused.<br>Initial methionine is missed
**Octopamine (Oct) beta receptor 1**	Octopamine	-	KQ034268	426	Partial	VectorBase predictions RPRC001507 and RPRC005349 fused.<br>Initial methionine is still missed
**Oct beta receptor 2**		RPRC011545	KQ034319	447	Yes	Initial methionine fixed
**Oct beta receptor 3**		-	KQ034653	375	Partial	VectorBase predictions RPRC014610 and RPRC001054 fused and edited. Initial methionine is still missed
**α2-adrenergic-like octopamine receptor**		RPRC015456	KQ034169	330	Partial	Initial methionine is missed.<br>No changes on VectorBase prediction
**Oct receptor in mushroom bodies (Oamb) -like**		RPRC001341	KQ034231	417	Partial	No changes in VectorBase prediction. Partial sequence
**Oct/Tyramine**	Octopamine/Tyramine	RPRC008712	KQ034100	455	Yes	The two exons of the VectorBase prediction fused
**Orphan receptor 1**	Unknown	RPRC004409	KQ034373	235	Partial	No changes in VectorBase prediction
**Orphan receptor 2**	Unknown	RPRC002007-8	KQ034058	201	No	Two VectorBase predictions fused

Gene	Ligand	VectorBase code	Scaffold	Aas.	Hit against the antennal *de novo* assemblies	Comments
**NEUROPEPTIDE RECEPTORS FAMILY A**						
**ACP receptor isoform A**	AKH Corazonin related peptide	-	KQ034104, KQ035406 and KQ034241	295	No	New gene model created based GenBank sequence Acc. N° KM975506 [2]
**ACP receptor isoform B**		-		451	No	New gene model created based on GenBank sequence Acc. N° KM975507 [2]
**ACP receptor isoform C**		-		430	No	New gene model created based on GenBank sequence Acc. N° KM975508 [2]
**AKH receptor**	Adipokinetic hormone	-	KQ034132	353	Yes	New gene model created based on GenBank sequence Acc. N° AIJ49751 [3]
**Allatostatin A receptor**	Allatostatin A	RPRC004708	KQ034532	404	No	VectorBase prediction updated according to GenBank sequence Acc. N° KM283241 [4]
**Allatostatin C receptor**	Allatostatin C	RPRC013486	KQ034333	419	Yes	The initial methionine fixed according antennal transcriptome
**Allatotropin receptor**	Allatotropin	-	KQ034097	306	Partial	New gene model created, which is partially in the GenBank sequence Acc. N° KF740716 [5]
**Bursicon receptor**	Bursicon	RPRC001663	KQ034113	688	Yes	No changes in VectorBase prediction
**CAPA receptor isoform A**	CAPA peptide	RPRC000516	KQ034065	385	No	Identical to GenBank sequence Acc. N° ADG27752 [6]
**CAPA receptor isoform B**	CAPA peptide	-	KQ034065	354	No	New gene model created based on GenBank sequence Acc. N° ADG27753 [6]
**CCH amide receptor 1**	CCHamide peptide	RPRC007766	KQ034099	331	No	No changes in VectorBase prediction
**CCH amide receptor 2**		RPRC000608	KQ034099	373	No	No changes in VectorBase prediction
**CNM amide receptor**	CNMamide peptide	RPRC001428	KQ034058	140	Partial	Partial sequence. No changes in VectorBase prediction
**Crustacean cardioactive peptide receptor 1**	CCAP	RPRC001248	KQ034056	374	Yes	Identical to GenBank sequence Acc. N° KC004225 [7]
**Crustacean cardioactive peptide receptor 2**		-	KQ034561 and KQ034059	188	No	VectorBase predictions RPRC000969 and RPRC012063 fused. Partial sequence
**Corazonin receptor**	Corazonin	RPRC000523	KQ034084	383	No	No changes in VectorBase prediction
**Corazonin receptor alfa isoform**		-	KQ034084	441	Yes	New gene model created based on alfa isoform GenBank sequence Acc. N° AND99324 [8]
**Corazonin receptor beta isoform**		-	KQ034084	419	No	New gene model created based on beta isoform GenBank sequence Acc. N° AND99325 [8]

Gene	Ligand	VectorBase code	Scaffold	Aas.	Hit against the antennal *de novo* assemblies	Comments
**Ecdysis triggering hormone receptor**	Ecdysis triggering hormone	RPRC000848, RPRC008652	KQ034066, KQ034378 and KQ034714	424	Yes	Two VectorBase predictions were fused and edited.
**FMRF receptor**	FaLP	RPRC001551	KQ034140	410	Yes	No changes in VectorBase prediction
**FaLPamide/Proctolin receptor**	FaLP/Proctolin	RPRC015267	KQ034074	345	Partial	No changes in VectorBase prediction. Partial sequence, initial methionine is still missed
**GPA2/GPB2 receptor**	GPA2/GPB5	RPRC007243	KQ034109	591	Partial	CTE was extended until STOP codon. Partial sequence, initial methionine is still missed
**Ion Transport 1 receptor**	-	RPRC004793	KQ034083	611	Yes	No changes in VectorBase prediction
**Kinin receptor 1**	Kinin	RPRC000494	KQ034056	414	Yes	Two first exons and last exon eliminated from VectorBase prediction according to our antennal transcriptome
**Kinin receptor 2**	-	-	KQ034861 and KQ034100	366	Yes	Two predictions RPRC008570 and RPRC008649 fused and [illegible]dited. Described as orphan by S Ons, A Lavore, M Sterkel, JP Wulff, I Sierra, JM Barnetche, MH Rodriguez and R Rivera-Pomar [9] as RPRC008570
**Long neuropeptide F receptor 1**	Neuropeptide F	-	KQ034119	390	Partial	New gene model created based on [10]
**Long neuropeptide F receptor 2**		RPRC008894	KQ034459	180	Partial	No changes in VectorBase prediction. Partial sequence, initial methionine is still missed
**Myoinhibitory peptide receptor isoform A**	Allatostatin B	RPRC000605	KQ034129	420	Partial	VectorBase prediction was identical to GenBank sequence Acc. N° KF958188 [11]
**Myoinhibitory peptide receptor isoform B**		-	KQ034129	324	No	No VectorBase prediction
**Myosuppressin receptor**	Myosuppressin	-	KQ034057	368	No	New gene model created based on GenBank sequence Acc. N° AGT02812 [12]
**Natalisin receptor**	Natalisin	RPRC001687	KQ034139	351	Yes	VectorBase prediction extended in NTE region according to antennal transcriptome
**Pyrokinin 1 receptor**	PBAN	RPRC008528	KQ034938	208	No	No changes in VectorBase prediction. Partial sequence.
**Pyrokinin 2 receptor isoform A**		-	KQ034161	345	Partial	New gene model created based on GenBank sequence Acc. N° AFO73269 [13]. Sequence partially represented at RPRC005110
**Pyrokinin 2 receptor isoform B**		-	KQ034161	444	Partial	New gene model created based on GenBank sequence Acc. N° AFO73270 [13]. Sequence partially represented at RPRC005110
**Pyrokinin 2 receptor isoform C**		-	KQ034161	414	Partial	New gene model created based on GenBank sequence Acc. N° AFO73271 [13]. Partially represented RPRC005110

Gene	Ligand	VectorBase code	Scaffold	Aas.	Hit against the antennal *de novo* assemblies	Comments
**Pyroglutamylate RFamide peptide receptor**	Rfamide peptides	-	KQ034100	395	Yes	New gene model created. First 100 amino acids in RPRC014460
**RYamide receptor**	Ryamide	-	KQ034249 and KQ034213	360	Partial	New gene model created based on Ons [13]. First 133 amino acids in KQ034249. Problem between 3rd and 4th exons
**Short neuropeptide F receptor**	Short neuropeptide F	-	KQ034095 and KQ035872	448	Yes	Three VectorBase predictions (RPRC002266, 002268 and 002269) fused
**Sulfakinin receptor 1**	Sulfakinin	-	KQ035199, KQ035392 and KQ034565	320	No	RPRC012816 (KQ034095) and RPRC003273 (KQ035392) represented different parts of gene model
**Sulfakinin receptor 2**		RPRC012816	KQ034565	138	No	No changes in VectorBase prediction. Partial sequence.
**SIFamide receptor**	SIFamide	RPRC000835	ACPB03024746	451	Yes	The first part of VectorBase prediction eliminated. Only the last 264 amino acids were present in the genome
**Tachykinin 86C receptor-like**	Tachykinin	RPRC008022	kQ035269	309	Yes	No changes in VectorBase prediction. Identified as ITP receptor by S Ons [14]
**Tachykinin 99D receptor-like**		-	KQ034874 and KQ034432	378	Yes	VectorBase predictions RPRC003160 and RPRC000651 were fused
**Orphan receptor 3**	-	RPRC014721	KQ034261	962	Partial	No changes in VectorBase prediction. Initial methionine is missed
**Orphan receptor 4**	-	RPRC004128	KQ035493	381	No	No changes in VectorBase prediction. Initial methionine is missed
**Orphan receptor 5**	-	RPRC008364	KQ034143	117	No	No changes in VectorBase prediction. Partial sequence
NEUROPEPTIDE RECEPTORS FAMILY B						
**Calcitonin-like diuretic hormone receptor 1 isoform B**	Dh31	RPRC009814	KQ035556 and KQ034793	411	Yes	First 77 amino acids located in KQ035556. Identical to GenBank sequence Acc. N° AHB86317 [15]
**Calcitonin-like diuretic hormone receptor 1 isoform C**		-	KQ035556 and KQ034793	409	Partial	First 77 amino acids located in KQ035556. New gene model included. Identical to GenBank sequence Acc. N° AHB86318 [15]
**Calcitonin-like diuretic hormone receptor 2**		RPRC004753	KQ034099	410	No	VectorBase prediction edited according to GenBank sequence Acc. N° AHB86571 [15]
**Calcitonin-like diuretic hormone receptor 3**	-	RPRC004735	KQ034099	420	Yes	Initial methionine fixed and an internal region was added to VectorBase prediction
**Corticotropin-releasing factor-related like diuretic hormone receptor 1**	Dh44	-	KQ034141 and KQ035235	465	Yes	New gene model created.

Gene	Ligand	VectorBase code	Scaffold	Aas.	Hit against the antennal *de novo* assemblies	Comments
**Corticotropin-releasing factor-related like diuretic hormone receptor 2 isoform A**	Dh44	-	KQ034325	385	No	VectorBase prediction modified according to GenBank sequence Acc. N° KU942308 [16]. NTE is missed. CTE region was extended.
**Corticotropin-releasing factor-related like diuretic hormone receptor 2 isoform B**	Dh44	RPRC000578	KQ034325	485	Yes	VectorBase prediction modified according to GenBank sequence Acc. N° KJ407397 [16]
**PDF receptor**	PDF	RPRC009680	KQ034059	318	Partial	The NTE region extended. Initial methionine is still missed
**Parathyroid hormone like receptor**	-	-	KQ034058	498	Yes	Predictions RPRC011083 and RPRC011086 fused and edited
**OTHER G-PROTEIN COUPLE RECEPTORS**						
**Long wave sensitive opsin 1 (LWS)**	-	RPRC010623	KQ034901	377	No	No changes in VectorBase prediction
**UV opsin**	-	RPRC002621	KQ034248	387	Yes	No changes in VectorBase prediction. Initial methionine is missed
**COpsin / Pteropsin**	-	RPRC017360	KQ034389	301	No	No changes in VectorBase prediction
**Rh7**	-	RPRC015283	KQ034074	361	Partial	No changes in VectorBase prediction. Initial methionine is missed
**Tyrosine kinase and guanylyl cyclase receptors**						
**Eclosion hormone receptor**	Eclosion hormone	RPRC013306	KQ034473	1160	No	No changes in VectorBase prediction. Initial methionine is missed
**NPLP receptor**	NPLP	RPRC013388	KQ034473	1159	Yes	NTE and CTE must be extended. Initial methionine is still missed
**Potential neuroparsin receptor***	Ovary ecdysteroidogenic hormone	RPRC006045	KQ034063	1290	Yes	The first exon was extended. Initial methionine was fixed. In VectorBase, initial methionine is missed due to an assembly problem
**Insulin receptor***	Insulin	RPRC006251	KQ034536	1198	Yes	Initial methionine must be fixed and some internal changes are necessary

* The sequences included in the Additional file 13: Data file S2 for these genes are those obtained after the comparison to our antennal transcriptome assemblies and the adequate correction. The gene models included for these genes in our GFF file were those available in VectorBase.

CT/DH receptor 1 (variant A) and CT/DH receptor 2 (variant A) reported by M Zandawala, S Li, F Hauser, CJ Grimmelikhuijzen and I Orchard [15] are a partial sequences (143 and 122 amino acids length, respectively), and they do not have VectorBase prediction. A partial CRF/DH receptor 1 variant A sequence was reported by H-R Lee, M Zandawala, AB Lange and I Orchard [16], however, it is not available in GenBank.

1. Paluzzi J-PV, Bhatt G, Wang C-HJ, Zandawala M, Lange AB, Orchard I: **Identification, functional characterization, and pharmacological profile of a serotonin type-2b receptor in the medically important insect, *Rhodnius prolixus***. *Front Neurosci* 2015, **9**:175.
2. Zandawala M, Haddad AS, Hamoudi Z, Orchard I: **Identification and characterization of the adipokinetic hormone/corazonin-related peptide signaling system in *Rhodnius prolixus***. *The FEBS Journal* 2015, **282**(18):3603-3617.
3. Zandawala M, Hamoudi Z, Lange AB, Orchard I: **Adipokinetic hormone signalling system in the C hagas disease vector, *Rhodnius prolixus***. *Insect Mol Biol* 2015, **24**(2):264-276.
4. Zandawala M, Orchard I: **Identification and functional characterization of FGLamide-related allatostatin receptor in *Rhodnius prolixus***. *Insect Biochem Mol Biol* 2015, **57**:1-10.
5. Alzugaray ME, Adami ML, Diambra LA, Hernandez-Martinez S, Damborenea C, Noriega FG, Ronderos JR: **Allatotropin: an ancestral myotropic neuropeptide involved in feeding**. *PLoS One* 2013, **8**(10):e77520.
6. Paluzzi JP, Park Y, Nachman RJ, Orchard I: **Isolation, expression analysis, and functional characterization of the first antidiuretic hormone receptor in insects**. *Proc Natl Acad Sci U S A* 2010, **107**(22):10290-10295.
7. Lee D, Broeck JV, Lange AB: **Identification and expression of the CCAP receptor in the Chagas' disease vector, *Rhodnius prolixus*, and its involvement in cardiac control**. *PLoS One* 2013, **8**(7):e68897.
8. Hamoudi Z, Lange AB, Orchard I: **Identification and characterization of the corazonin receptor and possible physiological roles of the corazonin-signaling pathway in *Rhodnius prolixus***. *Front Neurosci* 2016, **10**:357.
9. Ons S, Lavore A, Sterkel M, Wulff JP, Sierra I, Barnetche JM, Rodriguez MH, Rivera-Pomar R: **Identification of G-protein coupled receptors for opsines and neurohormones in *Rhodnius prolixus*. Genomic and transcriptomic analysis**. *Insect Biochem Mol Biol* 2015.
10. Sedra L, Paluzzi J-P, Lange AB: **Characterization and expression of a long neuropeptide F (NPF) receptor in the Chagas disease vector *Rhodnius prolixus***. *PLoS One* 2018, **13**(8):e0202425.
11. Paluzzi J-PV, Haddad AS, Sedra L, Orchard I, Lange AB: **Functional characterization and expression analysis of the myoinhibiting peptide receptor in the Chagas disease vector, *Rhodnius prolixus***. *Mol Cell Endocrinol* 2015, **399**:143-153.
12. Lee D, James T, Lange A: **Identification, characterization and expression of a receptor for the unusual myosuppressin in the blood feeding bug, *Rhodnius prolixus***. *Insect Mol Biol* 2015, **24**(1):129-137.
13. Paluzzi J-P, O'Donnell MJ: **Identification, spatial expression analysis and functional characterization of a pyrokinin-1 receptor in the Chagas' disease vector, *Rhodnius prolixus***. *Mol Cell Endocrinol* 2012, **363**(1-2):36-45.
14. Ons S: **Neuropeptides in the regulation of *Rhodnius prolixus* physiology**. *J Insect Physiol Paris* 2017, **97**:77-92.
15. Zandawala M, Li S, Hauser F, Grimmelikhuijzen CJ, Orchard I: **Isolation and functional characterization of calcitonin-like diuretic hormone receptors in *Rhodnius prolixus***. *PLoS One* 2013, **8**(11):e82466.
16. Lee H-R, Zandawala M, Lange AB, Orchard I: **Isolation and characterization of the corticotropin-releasing factor-related diuretic hormone receptor in *Rhodnius prolixus***. *Cellular Signalling* 2016, **28**(9):1152-1162.
