# Supplementary figures and images for: Transcriptomics supports local sensory regulation in the antenna of the kissing-bug Rhodnius prolixus
Source: BMC Genomics. 2020 Jan 30;21:101. doi: 10.1186/s12864-020-6514-3 (PMC6993403; doi:10.1186/s12864-020-6514-3)

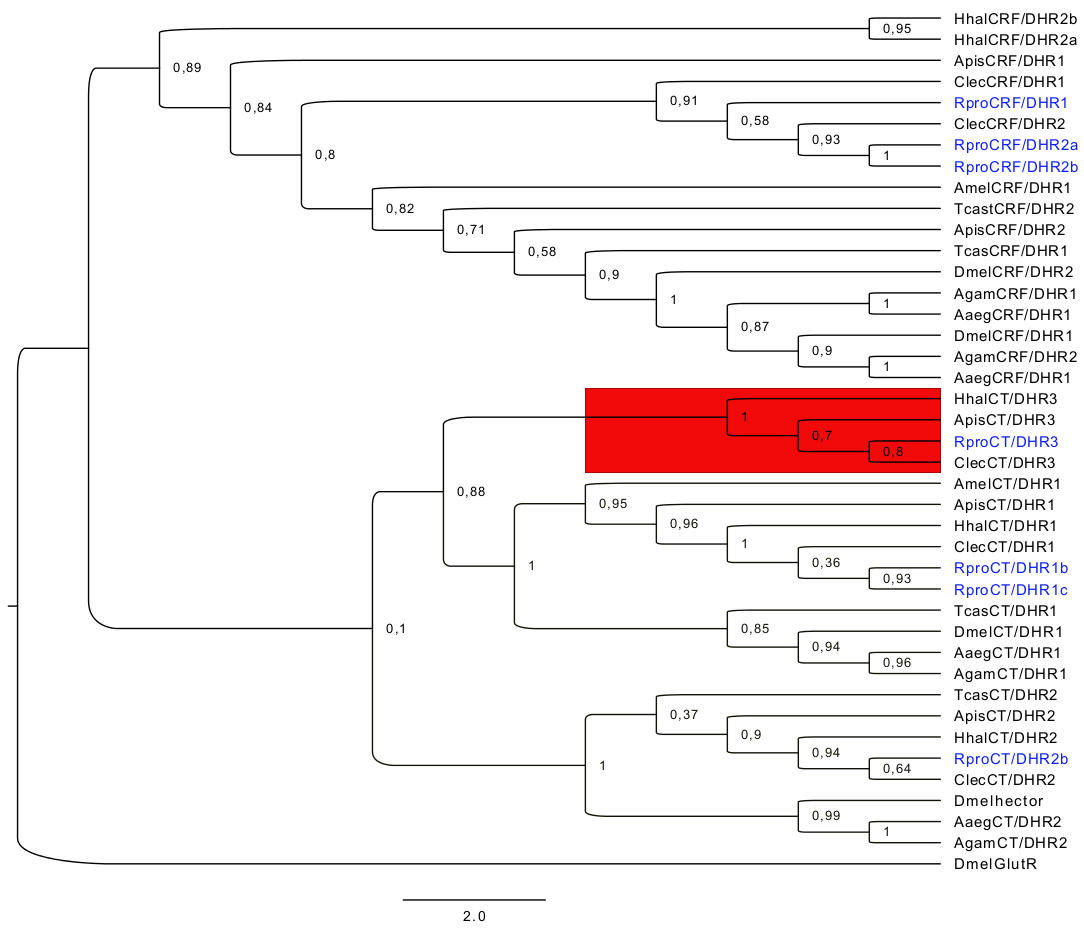

Supplement: Supplementary file 3 — Additional file 3: Figure S1. Molecular phylogenetic analyses of calcitonin diuretic (CT) and corticotropin-releasing factor-related (CRF) like diuretic hormone (DH) receptors of R. prolixus and other insects. The evolutionary history of R. prolixus CT/DH and CRF/DH receptors was inferred by using the maximum likelihood method in PhyML v3.0. The support values on the bipartitions correspond to SH-like P values, which were calculated by means of aLRT SH-like test. The CT/DH receptor 3 clade was highlighted in red. The CT/DH and CRF/DH R. prolixus receptors were displayed in blue. The LG substitution amino-acid model was used. Species abbreviations: Dmel, Drosophila melanogaster; Aaeg, Aedes aegypti; Agam, Anopheles gambiae; Clec, Cimex lecturiaus; Hhal, Halomorpha halys; Rpro, Rhodnius prolixus; Amel, Apis mellifera; Apis, Acyrthosiphon pisum; and Tcas, Tribolium castaneum. The glutamate receptor sequence from the D. melanogaster (FlyBase Acc. N° GC11144) was used as an out-group. The sequences used from other insects are reported in Additional file 15: Data file S4). [file 12864_2020_6514_MOESM3_ESM.tif]

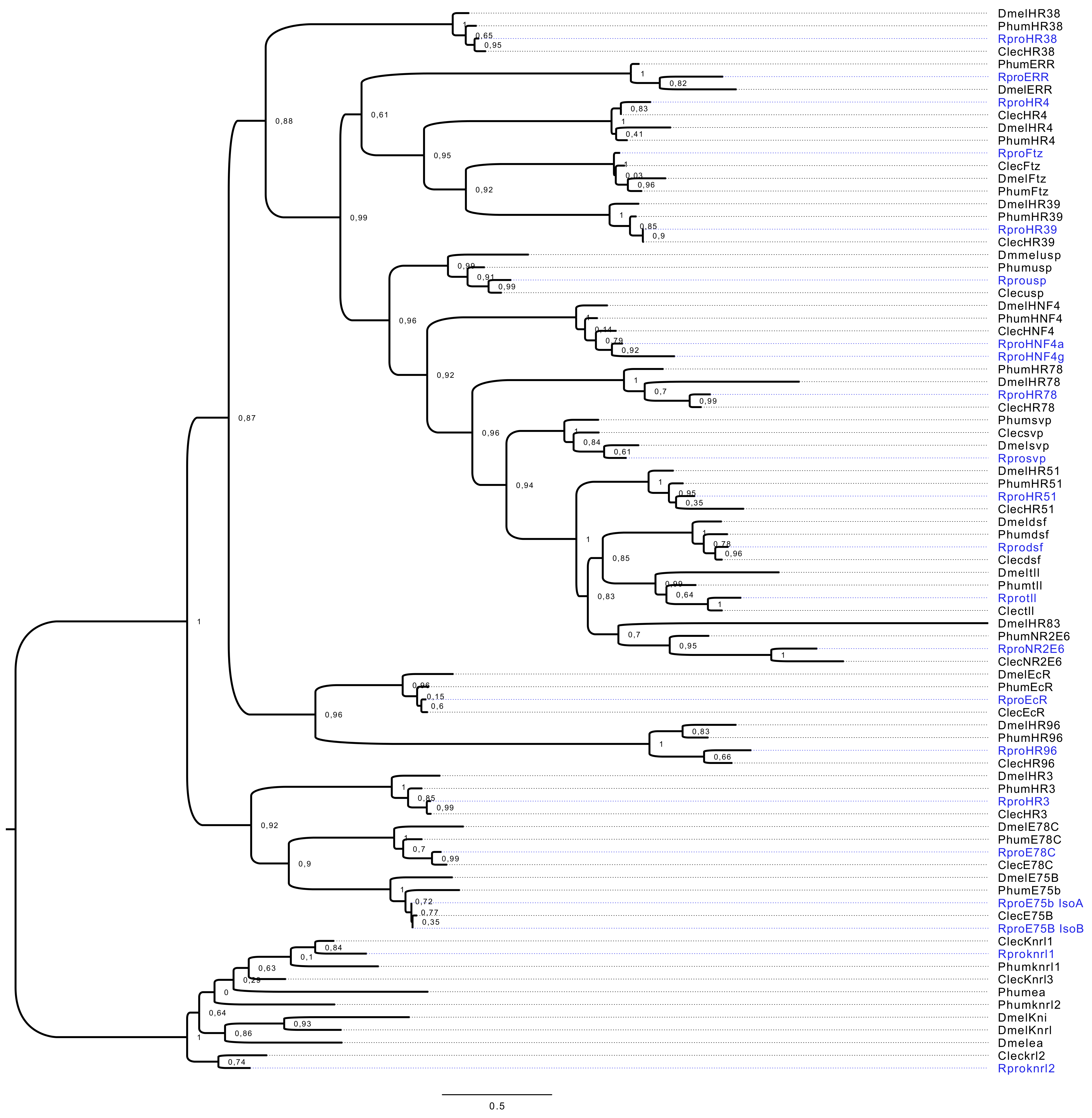

Supplement: Supplementary file 7 — Additional file 7: Figure S2. Molecular phylogenetic analysis of nuclear receptor genes of R. prolixus and other insects. The evolutionary history of R. prolixus nuclear receptors was inferred by using the maximum likelihood method in PhyML v3.0. The support values on the bipartitions correspond to SH-like P values, which were calculated by means of aLRT SH-like test. The R. prolixus nuclear receptors were displayed in blue. LG substitution amino-acid model was used. Species abbreviations: Dmel, Drosophila melanogaster; Phum, Pediculus humanus; Clec, Cimex lectularius. The RproEip75B sequence used was from isoform B (from our antennal transcriptome) because the sequence of isoform A available in VectorBase was considered incomplete. The sequences used from other insects are reported in Additional file 15: Data file S4). [file 12864_2020_6514_MOESM7_ESM.tif]

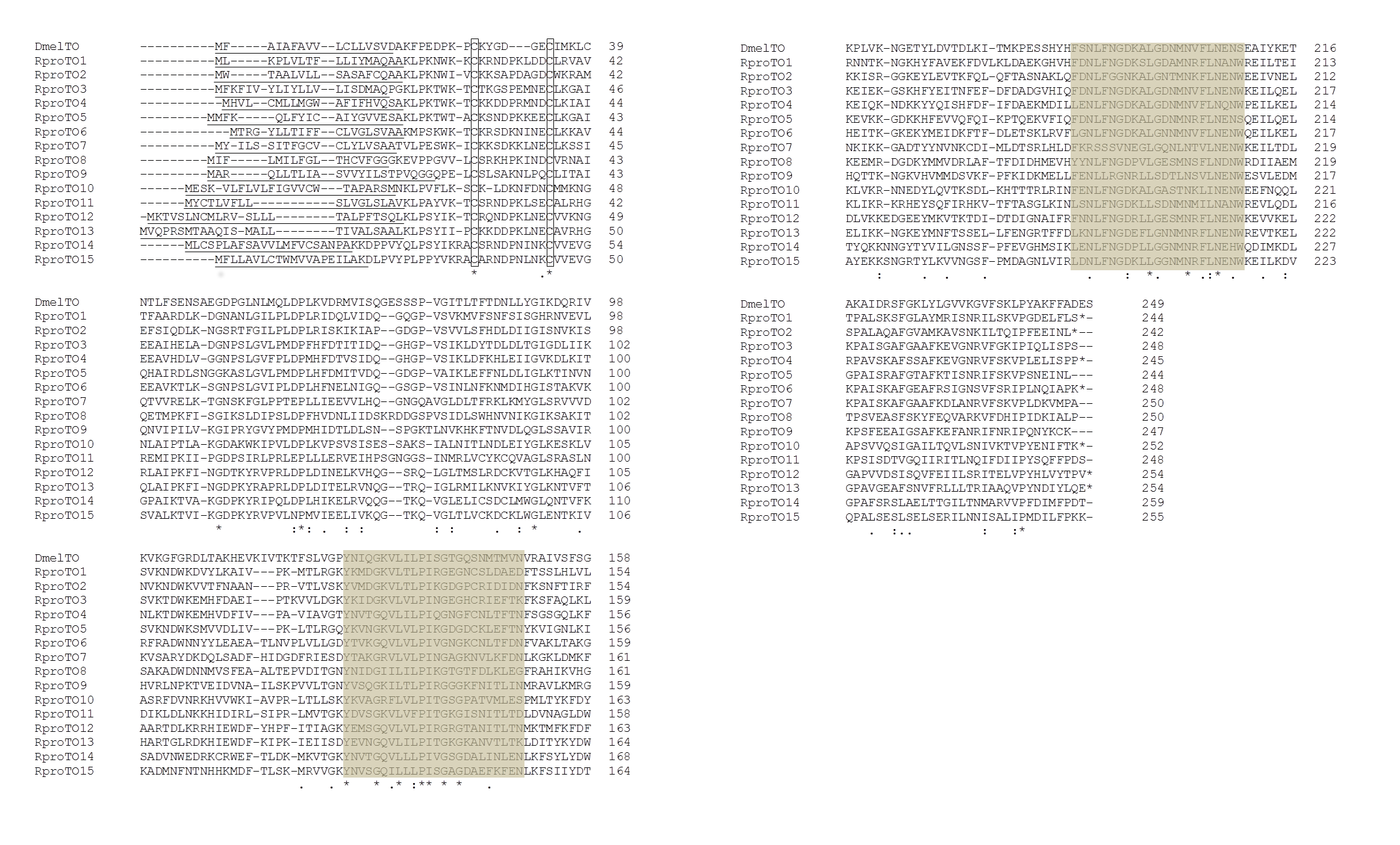

Supplement: Supplementary file 9 — Additional file 9: Figure S3. Alignment of R. prolixus takeout protein sequences. Sequences were aligned with CLUSTAL X v2.0. Asterisks indicate identical amino-acids, double points show conserved exchanges and single points show homologous amino acids. The D. melanogaster takeout protein sequence was obtained from Justice et al. [75]. The two conserved cysteine residues defining the takeout family [74] in many insects are marked with white boxes. The position of the conserved motifs 1 and 2 described by So et al. [34] is indicated with grey boxes. Predicted signal peptides are underlined. Species abbreviations: Rpro, Rhodnius prolixus; and Dmel, Drosophila melanogaster. [file 12864_2020_6514_MOESM9_ESM.tif]

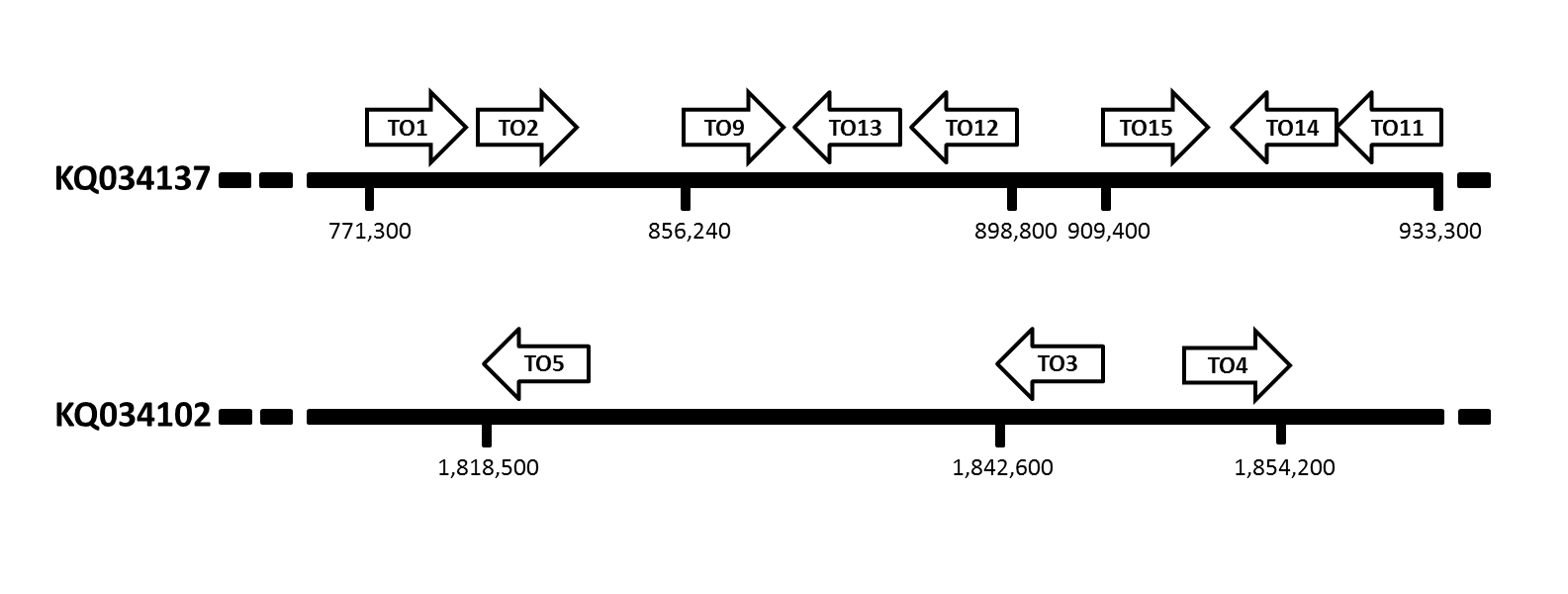

Supplement: Supplementary file 10 — Additional file 10: Figure S4. Structure and organization of takeout gene clusters. Scaffold IDs are presented on the left. White arrows represent each takeout gene and its position on the scaffold. [file 12864_2020_6514_MOESM10_ESM.tif]
