# Supplementary material for: Transcriptomics supports local sensory regulation in the antenna of the kissing-bug Rhodnius prolixus
Source: BMC Genomics. 2020 Jan 30;21:101. doi: 10.1186/s12864-020-6514-3 (PMC6993403; doi:10.1186/s12864-020-6514-3)
Supplement: Supplementary file 4 — Additional file 4: Table S3. Details of enzymes involved in the biogenic amine synthesis. Columns are: Gene – the gene and protein name we are assigning; VectorBase code – the official gene number in the RproC3 genome assembly, prefix is RPRC; Scaffold – the RproC3 genome assembly supercontig ID; AAs – number of encoded amino acids in the protein; Comments – comments on the OGS gene model and repairs to be performed on the genome assembly (available on VectorBase) based on BLAST searches against de novo antennal transcriptome assemblies. NTE: Amino-terminal region. [file 12864_2020_6514_MOESM4_ESM.pdf]

Table S3. Details of enzymes involved in the biogenic amine synthesis. Columns are: Gene – the gene and protein name we are assigning; VectorBase code – the official gene number in the RproC3 genome assembly, prefix is RPRC; Scaffold – the RproC3 genome assembly supercontig ID; AAs – number of encoded amino acids in the protein; Comments – comments on the OGS gene model and repairs to be performed on the genome assembly (available on VectorBase) based on Blast searches against *de novo* antennal transcriptome assemblies. NTE: Amino terminal region.

Gene	VectorBase code	Scaffold	AAs.	Hit against the antennal *de novo* assemblies	Comments
**Tyrosine 3-monooxygenase (*ple*)**	RPRC007034	KQ034272	569	Yes	Some internal problems were detected*
**DOPA decarboxylase (*Ddc*)**	RPRC005884	KQ034063	629	Yes	NTE region must be extended until initial methionine*
**Tyrosine decarboxylase-2 (*Tdc2*)**	RPRC011470	KQ034319	476	No	Fine as is
**Tryptophan hydroxylase (*Trh*)**	RPRC012297	KQ034056	490	No	Stop codon is missed

(*) The sequences included in the Additional file 13: Data file S2 for these genes are those obtained after the comparison to our antennal transcriptome assemblies and the appropriate correction.
