# Supplementary material for: Transcriptomics supports local sensory regulation in the antenna of the kissing-bug Rhodnius prolixus
Source: BMC Genomics. 2020 Jan 30;21:101. doi: 10.1186/s12864-020-6514-3 (PMC6993403; doi:10.1186/s12864-020-6514-3)
Supplement: Supplementary file 5 — Additional file 5: Table S4. Details of neuropeptide processing enzymes. Columns are: Gene – the gene and protein name we are assigning; VectorBase code – the official gene number in the RproC3 genome assembly, prefix is RPRC; Scaffold – the RproC3 genome assembly supercontig ID; AAs – number of encoded amino acids in the protein; Comments – comments on the OGS gene model and repairs to be performed on the genome assembly (available on VectorBase) based on BLAST searches against de novo antennal transcriptome assemblies. [file 12864_2020_6514_MOESM5_ESM.pdf]

Table S4. Details of neuropeptide processing enzymes. Columns are: Gene – the gene and protein name we are assigning; VectorBase code – the official gene number in the RproC3 genome assembly, prefix is RPRC; Scaffold – the RproC3 genome assembly supercontig ID; AAs – number of encoded amino acids in the protein; Comments – comments on the OGS gene model and repairs to be performed on the genome assembly (available on VectorBase) based on Blast searches against *de novo* antennal transcriptome assemblies.

Gene	VectorBase code	Scaffold	AAs.	Hit against the antennal *de novo* assemblies	Isoforms	Comments
**Signal Peptidase (*SP*)**	RPRC009668	KQ034208	375	Yes	No	CTE is missed
***Amontillado* (Prohormone convertase 2- *PC2*)**	RPRC009349	KQ034234	640	Yes	No	Initial methionine is missed
***Silver* (Carboxypeptidase D)**	-	KQ034072	1129	Yes	No	New gene model was created. RPRC011379, RPRC011383 and RPRC0011427 were fused. Partial sequence, methionine and stop codon are still missed*
**Prolyl endopeptidase**	RPRC006929	KQ034086	690	Yes	No	Methionine and stop codon missed. Identified using *D. melanogaster* sequence CG5355
**Carboxypeptidase M (*CPM*)**	RPRC015124	KQ034103	454	No	A	Methionine is missed. Identified using *D. melanogaster* sequence CG4678
			471	Yes	B	New isoform identified in the antennal transcriptome
			525	Yes	C	New isoform identified in the antennal transcriptome
**Peptidylglycine alfa-hydroxylating mono-oxygenase (*PHM*)**	-	KQ034112	329	Yes	No	VectorBase predictions RPRC001216 and RPRC001217 must be fused and edited
**Furin (*Fur*) like protease-1**	RPRC006957	KQ034094	810	Yes	No	A total of 126 amino acids are missed in NTE region. Some internal problems were detected
***Fur*-like protease 2A**	RPRC002472	KQ034090	1162	Yes	No	NTE and CTE are missed
***Fur*-like protease 2B**	RPRC013490	KQ034542	688	Yes	No	NTE and CTE are missed
**Peptidyl-alpha-hydroxyglycine alpha-amidating lyase 1 (*PAL1*)**	-	KQ034270	438	Yes	No	An internal region is missed due to problems in the genome assembly. CTE region located in the opposite strand*
**Peptidyl-alpha-hydroxyglycine alpha-amidating lyase 2 (*PAL2*)**	-	KQ034195	370	Yes	No	The first two exons located in the opposite strand*

* These models were fixed and included in the modified GFF file that was used for mapping of our RNASeq reads.

The sequences included in the Additional file 13: Data file S2 for all genes are those obtained after the comparison to our antennal transcriptome assemblies and the adequate correction.
