# Supplementary material for: Transcriptomics supports local sensory regulation in the antenna of the kissing-bug Rhodnius prolixus
Source: BMC Genomics. 2020 Jan 30;21:101. doi: 10.1186/s12864-020-6514-3 (PMC6993403; doi:10.1186/s12864-020-6514-3)
Supplement: Supplementary file 6 — Additional file 6: Table S5. Details of nuclear receptor genes. Columns are: Gene – the gene and protein name we are assigning; VectorBase code – the official gene number in the RproC3 genome assembly, prefix is RPRC; Scaffold – the RproC3 genome assembly supercontig ID; AAs – number of encoded amino acids in the protein; Comments – comments on the OGS gene model and repairs to be performed on the genome assembly (available on VectorBase) based on BLAST searches against de novo antennal assemblies. NTE: Amino-terminal region; CTE: Carboxyl-terminal region; VB: VectorBase. [file 12864_2020_6514_MOESM6_ESM.pdf]

Table S5. Details of nuclear receptor genes. Columns are: Gene – the gene and protein name we are assigning; VectorBase code – the official gene number in the RproC3 genome assembly, prefix is RPRC; Scaffold – the RproC3 genome assembly supercontig ID; AAs – number of encoded amino acids in the protein; Comments – comments on the OGS gene model and repairs to be performed on the genome assembly (available on VectorBase) based on Blast searches against *de novo* antennal transcriptome assemblies. NTE: Amino terminal region; CTE: Carboxyl terminal region.

Gene	VectorBase code	Scaffold	AAs.	Hit against antennal *de novo* assemblies	Comments
Knirps-related 1 (knrl1)	RPRC003216	KQ035852	325	No	Fine as is
Knirps-related 2 (knrl2)	-	ACPB3007969	302	Si	New gene model was created*
**Ecdysone-induced protein 75B *(Eip75B)* isoform A**	RPRC000853	KQ034727	717	No	Methionine is missed. Exon 4th was incorrect and 5th exon is located in the opposite strand.
**Ecdysone-induced protein 75B isoform B**	RPRC000853	KQ034727	653	Si	Only exons 2nd and 3rd were predicted in VectorBase. Exon 4th was incorrect and 5th exon is located in the opposite strand *
**Ecdysone-induced protein 78C *(Eip78C)***	RPRC009045	KQ034642	344	No	Initial methionine is missed
**Hormone receptor-like in 3 (*HR3*)**	RPRC003681 and RPRC0000824	KQ034284 and KQ036430	579	Si	Two VectorBase predictions must be fused and edited. NTE region must be extended until initial methionine*
**Ecdysone receptor (*EcR*)**	RPRC014174	KQ034515	475	Si	NTE region must be extended until initial methionine*
**Hormone receptor-like in 96 (*HR96*)**	RPRC001794	KQ034099	377	No	Fine as is
**Hepatocyte nuclear factor 4A (*HNF4*)**	RPRC008212	KQ034483	396	Si	CTE terminal region is incomplete. Initial methionine is missed
**Hepatocyte nuclear factor 4B (*HNF4*)**	RPRC001064	KQ034403	325	Partial	Fine as is
Ultraspiracle (usp)	RPRC013330	KQ034117	430	Si	NTE and CTE regions are incomplete*
**Hormone receptor-like in 78 (*HR78*)**	RPRC006737	KQ034201	495	Si	NTE and CTE regions are incomplete*
Tailless(tll)	RPRC007025	KQ034142	370	No	Initial methionine is missed
**Hormone receptor-like in 51 (*HR51* or *unfulfilled*)**	RPRC002557	KQ034474	478	No	Initial methionine is missed
Dissatisfaction (dsf)	RPRC010625	KQ034604	195	No	Initial methionine is missed
NR2E6 (PNR- like)	RPRC009755	KQ034409	394	Si	Multiple changes in VectorBase prediction are necessary*
Seven-up (svp)	RPRC000767	KQ034946	227	No	Fine as is
**Estrogen-related receptor (*ERR*)**	-	ACPB03009538	426	Si	New gene model was created*
**Hormone receptor-like in 38 (*HR38*)**	RPRC001680	KQ034154	287	Si	NTE is missed*
***Fushi tarazu transcription factor 1* (*Ftz-TF1*)**	RPRC001915	KQ034834 and KQ034274	618	Si	VectorBase predictions RPRC014120 and RPRC001915 must be fused and edited*
**Hormone receptor-like in 39 *(HR39)***	RPRC002968	KQ034115	695	Si	NTE region must be extended until initial methionine*
**Hormone receptor-like in 4 *(HR4)***	RPRC012796	KQ034081	542	Si	NTE region must be extended until initial methionine and CTE is missed

kni-like and, *PNR-like*, HR39**,** Estrogen-related receptor gene models were included in the modified GFF file that was used for mapping of our RNASeq reads.

(*) The sequences included in the Additional file 13: Data file S2 for these genes are those obtained after the comparison to our antennal transcriptome assemblies and the appropriate correction.
