# Supplementary material for: Transcriptomics supports local sensory regulation in the antenna of the kissing-bug Rhodnius prolixus
Source: BMC Genomics. 2020 Jan 30;21:101. doi: 10.1186/s12864-020-6514-3 (PMC6993403; doi:10.1186/s12864-020-6514-3)
Supplement: Supplementary file 8 — Additional file 8: Table S6. Details of takeout genes. Columns are: Gene – the gene and protein name we are assigning; VectorBase code – the official gene number in the RproC3 genome assembly, prefix is RPRC; Scaffold – the RproC3 genome assembly supercontig ID; AAs – number of encoded amino acids in the protein; Comments – comments on the OGS gene model and repairs to be performed on the genome assembly (available on VectorBase) based on BLAST searches against de novo antennal transcriptome assemblies. NTE: Amino-terminal region. [file 12864_2020_6514_MOESM8_ESM.pdf]

Table S6. Details of *to* genes. Columns are: Gene – the gene and protein name we are assigning; VectorBase code – the official gene number in the RproC3 genome assembly, prefix is RPRC; Scaffold – the RproC3 genome assembly supercontig ID; AAs – number of encoded amino acids in the protein; Comments – comments on the OGS gene model and repairs to be performed on the genome assembly (available on VectorBase) based on Blast searches against *de novo* antennal transcriptome assemblies. NTE: Amino terminal region.

**Gene**	**VectorBase code**	**Scaffold**	**AAs.**	**Hit against the antennal *de novo* assemblies**	**Comments**
to1	RPRC010098	KQ034137	244	Yes	Fine as is
to2	RPRC010096	KQ034137	242	Yes	Annotated as *to3*
to3	RPRC008440	KQ034102	248	Yes	The initial methionine must be fixed
to4	RPRC008432	KQ034102	191	Yes	Some internal problems detected
to5	RPRC008451	KQ034102	222	Yes	The initial methionine must be fixed
to6	RPRC002313	KQ034398	147	Yes	N-terminal region must be extended until initial methionine. Annotated as *to2*
to7	RPRC008276	KQ034251	250	Yes	Fine as is
to8	RPRC009613	KQ034205	250	Yes	Fine as is
to9	RPRC010085	KQ034137	247	Yes	Internal problems were fixed*
to10	RPRC011983 and RPRC011984	KQ034059	144	Yes	Two VectorBase predictions must be fused
to11	RPRC005773	KQ034137	248	Yes	Fine as is
to12	RPRC010201	KQ034137	226	Yes	Fine as is
to13	RPRC010202	KQ034137	227	Yes	Fine as is
to14	RPRC005774	KQ034137	259	Yes	Fine as is
to15	RPRC005775	KQ034137	255	Yes	Fine as is

* This model was fixed and included in the modified GFF file that was used for mapping of our RNASeq reads.

The sequences included in the Additional file 13: Data file S2 for *to3*, *to4*, *To5*, *to6*, *to9* and *to10* genes are those obtained after the comparison to our antennal transcriptome assemblies and the adequate correction.
