# Supplementary material for: Transcriptomics supports local sensory regulation in the antenna of the kissing-bug Rhodnius prolixus
Source: BMC Genomics. 2020 Jan 30;21:101. doi: 10.1186/s12864-020-6514-3 (PMC6993403; doi:10.1186/s12864-020-6514-3)
Supplement: Supplementary file 11 — Additional file 11: Data file S1. FPKM values of target genes in the three libraries. [file 12864_2020_6514_MOESM11_ESM.pdf]

	ANTENNAL FPKM VALUES		
**ANNOTATION**	**LARVAE**	**FEMALE**	**MALE**
ENZYMES INVOLVED IN BIOGENIC AMINE SYNTHESIS			
DOPA decarboxylase (*Ddc*)	3.4	14.1	18.7
Tyrosine decarboxylase-2 (*Tdc2*)	0.9	1.5	1.7
Tryptophan hydroxylase (*Trh*)	4.0	2.9	3.3
Tyrosine 3-monooxygenase (*ple*)	125.5	156.4	129.8
BIOGENIC AMINE RECEPTORS			
**Muscarinic Acetylcholine receptor type A**	5.8	9.3	14.4
Muscarinic Acetylcholine receptor type B	0.2	0.2	1.2
Muscarinic Acetylcholine receptor type C	12.8	14.6	15.4
Dopamine 1-like receptor 1	1.7	1.1	2.4
Dopamine 1-like receptor 2	1.6	2.4	0.2
Dopamine 2-like receptor	0.0	0.0	0.1
Octopamine receptor in mushroom bodies	0.1	0.3	0.5
Dopamine ecdysone receptor	6.6	14.9	45.0
**Octopamine beta receptor 1**	2.1	2.0	6.8
Octopamine beta receptor 2	2.5	3.5	9.0
Octopamine beta receptor 3	0.5	1.3	5.8
Octopamine-Tyramine receptor	1.5	0.9	2.6
α2-adrenergic-like octopamine receptor	0.0	0.0	0.0
**Serotonin receptor 1A**	0.2	0.3	1.4
**Serotonin receptor 1B**	7.2	7.0	11.4
**Serotonin receptor 2A**	0.3	0.1	0.9
Serotonin receptor 2B	23.7	35.8	37.6
**Serotonin like receptor 7**	1.5	1.6	8.1
Orphan receptor 1	12.0	11.5	18.2
**Orphan receptor 2**	0.3	0.3	1.2
OPSINS			
COpsin/Pteropsin	0.0	0.0	0.2
Long wave sensitive opsin 1	1.5	1.0	1.4
Rh7	0.4	0.6	0.9
UV opsin	3.0	1.9	1.9
NEUROPEPTIDE PRECURSOR GENES			
Adipokinetic hormone/corazonin-related peptide	0.0	0.0	0.0
Adipokinetic hormone (3 variants included)	0.4	0.3	0.5
Allatotropin	7.3	19.2	22.9
Allatostatin CCC	36.5	33.7	22.0
Allatostatin CC	888.4	98.5	54.9
Allatostatin A	22.1	1.1	0.7
Bursicon alfa	0.3	0.1	0.0
Bursicon beta	0.1	0.0	0.2
**Diuretic hormone 31 (3 isoforms included)**	3.4	3.3	4.8
Cardioacceleratory peptide alfa	0.1	0.0	0.1
Cardioacceleratory peptide beta	0.03	0.1	0

	ANTENNAL FPKM VALUES		
**ANNOTATION**	**LARVAE**	**FEMALE**	**MALE**
Crustacen cardiactive peptide	11.3	9.8	4.5
CCHamide	2.6	2.9	3.4
CNMamide	1.2	1.2	2.7
Corazonin	0.1	0.0	0.0
Diuretic hormone 44	7.7	10.7	12.4
Ecdysis triggering hormone	0.3	0.0	0.0
Eclosion hormone	0.0	0.0	0.0
Elevenin-1	0.4	1.2	1.0
Elevenin-2	4.3	11.0	5.1
FLP	0.2	0.1	0.2
GPA2	10.5	14.0	17.5
GPB5	1.4.	1.1	1
kinin (Leucokinin)	8.7	7.2	6.0
Ion transport peptide (ITP)	7.5	20.1	12.0
Insulin-like peptide	0.9	0.7	2.4
ITG-like	60.4	47.3	129.1
LNPF	2.5	5.1	2.6
**MIP**	24.8	1.2	1.4
Myosuppressin	5.0	3.0	6.0
Natalisin	2.4	2.3	2.1
Neuroparsin	5.1	0.7	11.5
Neuropeptide like precursor 1	7.7	7.1	7.9
NVP-Like	11.0	13.4	14.2
Orcokinin (3 isoforms included)	15.3	56.4	101.0
**Pyrokinin**	0.0	0.1	0.2
PDF	9.6	5.8	8.1
Proctolin	0.4	0.0	0.3
IDLSRF-peptide	26.9	47.6	136.2
RYamida	3.9	8.9	8.0
SIFa	0.4	1.3	0.2
**sNPF**	0.2	2.5	1.9
Sulfakinin	0.0	0.2	0.0
Tachykinin	3.8	8.1	4.8
NEUROPEPTIDE PROCESSING ENZYMES			
**Peptidylglycine alfa-hydroxylating mono-oxygenase (PHM)**	159.5	213.0	210.7
Amontillado (Prohormone convertase 2)	5.9	10.1	15.6
Signal peptidase (SP)	76.9	71.1	89.6
**Silver**	21.1	16.6	34.4
Prolyl endopeptidase	30.5	22.7	59.0
Carboxypeptidase M (CPM) (3 isoforms included)	335.6	264.8	383.4
Furin like protease 1	113.7	95.7	92.4
Furin like protease 2A	3.9	3.8	18.9
Furin like protease 2B	12.6	4.0	6.9

	ANTENNAL FPKM VALUES		
**ANNOTATION**	**LARVAE**	**FEMALE**	**MALE**
**PAL1**	35.9	49.8	54.1
**PAL2**	32.5	75.6	62.4
**NEUROPEPTIDE AND NEUROHORMONE RECEPTORS FAMILY A**			
**AKH Corazonin related peptide receptor**	0.5	0.5	1.3
Adipokinetic hormone receptor (two isoforms included)	3.9	0.3	0.6
Allatotropin receptor	0.4	0.7	0.9
AstA receptor	0.2	0.0	0.3
AstC receptor	0.7	0.7	0.7
Cardioacceleratory peptide receptor (isoforms B and C)	0.3	0.6	1.3
CCHamide receptor 1	0.0	0.0	0.1
CCHamide receptor 2	0.0	0.0	0.0
CNMamide receptor	0.7	0.7	2.4
**Crustacean cardioactive peptide receptor 1**	12.1	9.8	14.1
**Crustacean cardioactive peptide receptor 2**	0.0	0.0	0.2
Corazonin receptor (3 isoforms included)	8.0	3.7	8.0
Ecdysis triggering hormone receptor	0.5	0.5	1.4
FaLPa/Proc receptor	0.7	1.2	1.0
FMRFamide receptor	0.7	0.9	3.0
Ion Transport peptide receptor	11.3	5.2	5.9
GPA2/GPB5 receptor	87.9	54.1	32.5
Kinin receptor 1	1.3	1.5	2.2
**Kinin receptor 2**	0.7	7.4	14.1
LNPF receptor 1 (only isoform 1 included in the GFF)*	13.5	31.8	39.8
LNPF receptor 2	0.0	0.1	0.4
Lutropin-choriogonadotropic hormone receptor-Bursicon receptor	0.3	0.3	1.3
Myoinhibitory peptide receptor (2 isoforms included)	0.8	0.6	1.3
Myosuppressin receptor	0.0	0.0	0.0
Natalisin receptor	1.7	2.9	5.4
Orphan receptor 3	0.4	0.2	0.7
Orphan receptor 4	0.2	0.2	0.1
Orphan receptor 5	0.6	1.9	5.2
Pyrokinin 2 receptor (3 isoforms included)	5.2	1.9	9.9
Pyrokinin 1 receptor	0.3	0.4	0.1
CRF receptor	19.5	4.8	6.9
**RYamide receptor**	3.2	3.3	3.9
SIFamide receptor	0.5	0.7	2.4
**SNPF receptor**	5.3	1.6	1.5
**Sulfakinin receptor 1**	0.1	0.3	0.2
Sulfakinin receptor 2	0.2	0.2	0.9
Tachykinin 86C receptor	2.1	7.2	5.8
**Tachykinin 99D receptor**	0.2	1.3	1.4

	ANTENNAL FPKM VALUES		
**ANNOTATION**	**LARVAE**	**FEMALE**	**MALE**
**NEUROPEPTIDE AND NEUROHORMONE RECEPTORS FAMILY B**			
**Calcitonin-like diuretic hormone receptor 1 (isoforms B and C)**	41.8	81.1	116.7
Calcitonin-like diuretic hormone receptor 2	0.2	0.3	0.6
Calcitonin-like diuretic hormone receptor 3	7.5	79.3	131.6
**Corticotropin-releasing factor-related like diuretic hormone receptor 1**	6.5	6.1	10.9
Corticotropin-releasing factor-related like diuretic hormone receptor (isoforms B and C)	4.1	14.8	29.2
Parathyroid hormone like receptor	12.2	14.7	13.5
PDF receptor	1.2	1.7	6.1
**TYROSINE KINASE AND GUANYLYL CYCLASE RECEPTORS**			
Eclosion hormone receptor	0.3	0.3	0.3
Insulin receptor	0.9	0.6	0.9
NPLP receptor	1.3	0.7	1.3
Potential neuroparsin receptor	6.2	1.6	8.2
**NUCLEAR RECEPTOR GENES**			
Dissatisfaction	0.0	0.0	0.0
Ecdysone receptor	4.4	2.2	7.1
Ecdysone-induced protein 75B (isoform A and B included)	111.2	44.7	101.6
Ecdysone-induced protein 78C	0.1	0.8	0.5
Estrogen-related receptor	2.8	2.2	5.9
Ftz transcription factor 1	1.5	1.0	8.0
Hepatocyte nuclear factor 4 A	29.0	25.2	43.2
Hepatocyte nuclear factor 4 B	1.4	0.1	0
**Hormone receptor-like in 38**	1.8	1	4
Hormone receptor-like in 39	11.6	4.8	14.4
Hormone receptor-like in 4	1.1	0.9	4.9
**Hormone receptor-like in 3**	2.1	7.9	26.4
Hormone receptor-like in 51	0.2	0.1	0.2
Hormone receptor-like in 78	6.4	2.3	11.6
Hormone receptor-like in 96	24.8	11.6	24.0
Knirps related 1	26.0	6.3	7.9
Knirps- related 2	0.3	0.2	0.2
NR2E6	3.5	17.1	16.7
Seven up	0.2	0.2	0.5
Tailless	0.9	0.2	0.1
Ultraspiracle	35.8	11.2	29.5
**TAKEOUT GENES**			
to1	2703.7	3575.1	2862.7
to2	34671.6	3919.8	2425.5
to3	134.7	4271	3082.8
to4	5200	20859.5	14346.7
to5	1.0	1.7	1.6
to6	358.8	373.2	369.1

	ANTENNAL FPKM VALUES		
**ANNOTATION**	**LARVAE**	**FEMALE**	**MALE**
to7	204.2	380.5	453.8
to8	734.8	5004.6	4869.7
to9	23.4	15.2	16.7
**to10**	3.7	40.1	42.2
to11	161.4	5.8	6.8
to12	3.8	20.4	25.1
to13	37.5	61.1	76.7
to14	469.8	738.0	519.4
to15	390.0	1059.7	946.2

Note:

Genes in bold are divided in two or more fragments on the *R. prolixus* genome assembly, so FPKM values were calculated by means of the sum of the counts from the different fragments divided for length of the complete coding sequence.

* Long Neuropeptide F receptor 1 isoform B was not included in the GFF file due to the sequence of the genome scaffold, which not included the position 387 (N for isoform 1 and S for isoform 2).
