# Supplementary material for: Transcriptomics supports local sensory regulation in the antenna of the kissing-bug Rhodnius prolixus
Source: BMC Genomics. 2020 Jan 30;21:101. doi: 10.1186/s12864-020-6514-3 (PMC6993403; doi:10.1186/s12864-020-6514-3)
Supplement: Supplementary file 12 — Additional file 12: Table S7. Differentially expressed modulatory genes among stages studied. Comparison of normalized CPM among stages was conducted using edgeR package. CPM: count per million, L: larval; F: female, M: male; LogFC: Log fold change; FDR adjusted p-value: False Discovery Rate; n.s.: not significant. [file 12864_2020_6514_MOESM12_ESM.pdf]

Table S7 - Differentially expressed modulatory genes among stages studied. Comparison of normalized CPM among stages was conducted using edgeR package. CPM: count *per* million, L: larval; F: female, M: male; LogFC: Log fold change; FDR adjusted p value: False Discovery Rate; n.s.: not significant.

**Annotation**	**VectorBase Code**	**CPM in L library**	**CPM in F library**	**CPM in M library**	**LogFC L *vs.* F**	**LogFC L *vs.* M**	**LogFC F *vs.* M**	**All comparisons**		**L *vs.* F pairwise comparison**		**L *vs.* M pairwise comparison**		**F *vs.* M pairwise comparison**	
								**p value**	**FDR adjusted P value**	**p value**	**FDR adjusted p value**	**p value**	**FDR adjusted p value**	**p value**	**FDR adjusted p value**
Allatostatin-A	-	13.52	0.77	0.34	4.121	5.301	1.179	7.16E-08	1.05E-05	9.57E-05	0.009	2.62E-06	3.56E-04	0.247	0.993 (n.s.)
Myoinhibitory peptide	-	16.25	0.90	0.77	4.171	4.392	0.221	7.13E-07	8.43E-05	7.80E-05	0.008	3.86E-05	0.003	0.820	0.993 (n.s.)
Adipokinetic hormone receptor	-	9.65	0.77	1.25	3.634	2.948	-0.686	1.48E-04	0.008	4.48E-04	0.032	0.003	0.090 (n.s.)	0.478	0.993 (n.s.)
Calcitonin-like diuretic hormone receptor 3	RPRC004735	12.21	119.18	145.18	-3.287	-3.571	-0.285	0.001	0.050	9.93E-04	0.055 (n.s.)	4.12E-04	0.020	0.775	0.993 (n.s.)
Kinin receptor 2	-	0.73	9.27	12.96	-3.662	-4.146	-0.483	3.81E-04	0.017	3.99E-04	0.029	8.56E-05	0.006	0.599	0.993 (n.s.)
Rproto11	RPRC005773	123.61	5.12	4.45	4.592	4.796	0.203	3.83E-08	5.93E-06	1.55E-05	0.002	7.83E-06	8.68E-04	0.825	0.993 (n.s.)
Rproto3	RPRC008440	107.42	3930.32	2090.78	-5.193	-4.283	0.911	1.19E-05	9.53E-04	1.92E-06	4.42E-04	4.11E-05	0.004	0.322	0.993 (n.s.)
HNF4B	RPRC001064	1.33	0.07	0.00	4.103	8.879	4.776	3.68E-07	4.65E-05	6.61E-04	0.041	2.72E-06	3.65E-04	0.054	0.993 (n.s.)
Allatostatin-CC	RPRC000300	454.29	58.15	23.87	2.966	4.250	1.285	1.66E-05	0.001	0.92	1 (n.s.)	8.77E-05	0.006	0.166	0.993 (n.s.)
Hormone receptor-like in 3	RPRC000824 RPRC003681	0.23	1.60	4.58	-2.787	-4.305	-1.518	3.56E-04	0.017	0.008	0.219 (n.s)	4.61E-05	0.004	0.111	0.993 (n.s.)
Octopamine beta receptor 3	-	0.52	1.75	5.63	-1.742	-3.426	-1.684	0.003	0.075	0.078	0.785 (n.s.)	9.10E-04	0.036	0.775	0.993 (n.s.)
Rproto2	RPRC010096	16437.45	2145.13	978.26	2.938	4.071	1.133	2.98E-05	0.002	0.003	0.112 (n.s.)	8.21E-05	0.006	0.022	0.993 (n.s.)
