# Supplementary material for: Transcriptomics supports local sensory regulation in the antenna of the kissing-bug Rhodnius prolixus
Source: BMC Genomics. 2020 Jan 30;21:101. doi: 10.1186/s12864-020-6514-3 (PMC6993403; doi:10.1186/s12864-020-6514-3)
Supplement: Supplementary file 15 — Additional file 15: Data file S4. Fasta sequences from different insects used in the phylogenetic analyses of CT/DH – CRF/DH and nuclear receptor genes. [file 12864_2020_6514_MOESM15_ESM.pdf]

CT and CRF DH receptor sequences
==================================
>ClecCT/DHR1-CLEC000667
MTDDVSNETYIDLNADILNVRRTQCLEFINESKPLEGLHCEPVFDTWTCWPATLAGSVATNPCPHFIVGFDPSRKAHKVCTE
NGTWFRHPESGQIWSNYTTCVNLDDLSLRQQVNNIYQAGYFISLLALLLSLFILSYFKSLRCPRNTLHMNLFTSFAFNNFLWL
LWYRLVIPNTDVIVKNRVWCQCLHVVLHYFLACSYAWMLAEGVYLHTLLVSAFTSEQKLVKILTACSWLVPFFFTALYTTLRL
ASGDTQQCWIDESDSKMVLFVLVATSMLLNFLFLCNIVRVVVTKLRAGPNQSARPSTALLQALRATLLLLPLLGLNYLLTPFR
PPDKHPWETYYEVVSALTASFQGLCVATLFCFCNGEVIAQMKRKWQYSVFRTRANSYTATTVSKSVYLQFVRSTAAPAAEE
EIV
>HhalCT/DHR1-XP_024216452.1
DSDFLQCAIYINESKSKNLEGSYCEATWDGWSCWQETPAGTTAYAHCPKFITGFDPNLLAHKICTENGTWFKHPDSGMV
WSNYTTCVNIEDLTLRQTVNNIYQTGYSISLVALLLSLFILSYFKALRCPRNTLHKNLFTAFAANNFLWLLWYRLIVPFPEVVIE
NGVWCQCLHVILHYFLLSCYAWMLAEGVYLHTLLVSAFTSEQKLVRILTVFSWTAPLFFIFLYSVLRLVFDDTDQCWINDSDY
SSVLVVLVVASMGLNLGFLCNIVRVVVGKLRAGPSQSSRPSQALLQALRATLLLLPLLGLNYLLTPFRPPNDHPLESYYEILSAF
TASFQGLCVATLFCFLNGEVMAQIKRKWQYATFRTRANSYTATTVSFVRSTAAPTAEEENV
>ApisCT/DHR1-XP_016661057.1
IVGTAYKICNKNATWFKHPISGAVWSNYTTCINHEDYNWTQQINTIYQTGYLVSFIALLLSIAILTYFKSLRCARNTLHTHMFT
SFAINNLLWLLWYRLVVEHPSVVLHNGWWCQILHVILHYFLLTNYAWMLAEGFYLHTLLVFAFTSEDTLVRWSWTLAWST
PLVVISLYTLLRTIYDHTSECWINESPFTEVLVVPVCMSMALNLVFLCNIVRVLWVKLQAGPSHLSNSTPSRTLLQAFRATLLL
LPLLGLHYLLTPFRPPKQHPWEPFYEVVSATTSSFQGLCVATLFCFLNGEVVAQIKRRWQFMFFRTRANSYTATTVSVRQFV
RSTAGPGDNEDKV
>TcasCT/DHR1-TC002694
MEQMRQHCLVFYKCEKDGEWNFNEQFNKSWVNYTTCINIEDFEFRQQIILIYCVGYGVSLVALLVSLALLTYFKSLRCARITV
HMNLFSSFAMNNFLWLLWYSLVVNDQDVLHENKLWCRVLHVVLFTFLISNYSWMLCEGIYLHTVLVSAFISERRLLRCMLA
LGWGIPLLTTSIYAPVRSVLGENVDELGRCWTQDGRFNKILMVPVVITVFLNVIFLVNIVRVLLIKLRKGPANGGSGSGASRT
SLQALRATMLLVPLLGLNFLLTPFRPEANHPWEYVYEVVSALTASLQGIQPFLMFFWYRDQNHSW
>DmelCT/DHR1-CG32843
MSDQIGNPNATFSGSGSGSGTNVASIAESVAESGPDFDALRAACETRLNASGQLAGSGGPGAEAGTHCAGTFDGWLCW
PDTAVGTSAYELCPDFITGFDPARYAHKECGLDGEWFKHPLTNKTWSNYTTCVNLEDLNWRHTVNLISEVGYGTSLLAILLS
LAILGYFKSLKCARITLHMNLFASFAANNSLWLVWYLLVMPNSELLHQSPMRCVALHITLHYFLLSNYSWMLCEGFYLHTVL
VAAFISEKRLVKWLIAFGWGSPAIVIFVYSMARGLGGTPEDNRHCWMNQTNYQNILMVPVCISMFLNLLFLCNIVRVVLLK
LNAPASIQGSCGPSRTVLQAFRATLLLVPLLGLQYILTPFRPAPKHPWENTYEIISAFTASFQGLCVAILFCFCNGEVIAQMKR
KWRMMCFSNRPRTNSYTATQVSFVRCGPPLPGEEKV
>AmelCT/DHR-LOC412591
MILSMRPSPTWLKMDVQTEDIVLLAGEQGRIRITITIVQISRLISEVANLISNMNASLKRCGHLASNFTKTLYCLMPANATAG
STLPDEEIHRIIMEREQECLQLEAMNSTPPPGPYCRLTFDGWSCWPNTPAGATAYVPCPNFITGFDASLRAHKYCELNGTW
FRHPESGQVWSNYTTCVNLKDLSWQQGINGLYEAGYAISLIALLLSLGILTYFRSLRCARITLHMNLFASFAVNNALWLVWY
RCIVANTDLLLNNGMTCRLLHIVLHYFLLTNYAWMLCEGFYLHTLLVSAFTSEQKLVKWLMILGWPVPAIIVTIYACLRATSN
DLTDTEQCWINEGNYMNVLVYPVCVSTLLNVLFLFNIVRVLLMKLRAGPSIGTQPSRSMRQAFRATLLLVPLLGLHYLVIPFR
PPKNHPWEHFYEVLSAITASFQGLCVAILFCFCNGEVIAQFKRKWEGSAFLRNRANSCTATTVSVRQIHKT
>AgamCT/DHR1-AGAP009770
AKHRYKPTRGTLQVTNPRTIPFTALNVEPHIRFPTKLSSSRVKTTSSPQPRNSMALSTTTDASVDEIAQKRLECLETLNETVDT
TISGFAHKVCSENGEWFRHPETNRSWSNYTTCINLDDFEAGCITLHLVLHYFLLTNYSWMLCEGFYLHTVLVSAFVSEKKLLK
WLLALGWGSPAFFIVLYGFLRGYASPPNDTIECWMNDSSFNKVFVGPVCISMLLNLVFLFNIMRVLLLKLKAPAGPQGAGP
SRTILQAFRATLLLVPLLGLQYILTPFRPGNGHPYERTYETMLACTSSFQGLFVAVLFCFLNGEVIAQVKRKWRTVFLRTRANS
YTATQVSVSTLTVLRV
>AaegCT/DHR1-AAEL010043
MTSSTTTDATDSEFDQRKLQCLEMLNATTEFTTTRSGPFCRGTWDGWLCWPDTAAGESALLPCPDFMDGFDPTRFAHKD
CDEDGEWFRHPLTNRTWSNYTTCVNLDKLEWMEQVRTIYETGYSISLIALILSLGILSYFRSLKCARITLHMNLFASFASNNTL
WLLWYRMVLADPEVLKHNGASCITLHLVLHYFLITNYAWMLCEGFYLHTVLVSAFVSEKKLVNWLVVLGWTTPGIVIMAY
GFLRGYAGTPEDTIECWMNESVYDNVFKAPVCISMLLNLLFLCNIIRVVLLKLKAPAGPQGTGPSRTILQAFRATLLLVPLLGL
QYILTPFRPDPGHSYERTYEIISAFTASFQGLFVAVLFCFFNGEVIAQVKRKWRTVFLRTRTNSYTATQVSPVRLQFIRSGPPIP
GEEKV
>Dmelhector-CG4395

MATTSSDSESQNVDVASQAQTQDNLRIFLKHLYAECVFRYQNVTYDTDDPSFSLGPATDYDSDLPENFSPVPRYLENAAM
NEGVIDMRNVDEELAEKEELMATVVSATMATNQKENRLFCPLNFDGYLCWPRTPAGTVLSQYCPDFVEGFNRKFLAHKT
CLENGSWYRHPVSNQTWSNYTNCVDYEDLEFRQFINELYVKGYALSLLALLISIIIFLGFKSLRCTRIRIHVHLFASLACTCVAW
ILWYRLVVERSETIAENPLWCIGLHLVVHYFMLVNYFWMFCEGLHLHLVLVVVFVKDTIVMRWFIVISWFSPIPIAIVYGLAR
HFSSPDNKHCWITDSLYLWIFSVPITLSLLASFIFLINVLRVIVRKLHPQSAQPAPLAIRKAVRATIILVPLFGLQHFLLPYRPDAG
TQLDHFYQMLSVVLVSLQGFVVSFLFCFANHDVTFAIRTLLNKLLPSLVTPPPAGSNTGQMATTTPSRELGV
>ClecCT/DHR2-CLEC025204
MRAYCPAAFDGWSCWNTTPSGETALAPCPYFVTGFDIKRFAFRKCLDNGSWFRHPDTGQPWSNYTTCIDMDDLEVREIV
NTIYVVGYYVSFAALVLSLLIFLTFRSLRCTRIAIHIQLFSSFAANNLMWIIWYKLVVGNTSVVQRNQPFCQILHIVLQYLMVA
NYLWMFCEGLHLHLALVVVFVKDNSAMRWFYCIGWVFPGILTAIYASVRYWYTEETRQCWMNESHTQWILTAPVCFSM
LASLGFLINVVRVLLTKLHCNSANPAPIGLRKAVRAALILVPLFGIHHILIPFRPEPNAPGERAYQIFSALLVSLQVIISYRYKNTG
KVLMFKCLSISDRTATIKPNFN
>HhalCT/DHR2-XP_024217979.1
MKLKRECEIRKRHQLEMFPTKVADRTGLRPYCAATFDGWSCWNTTPSGEIALAPCPNFVTGFDTKRFAFRKCMDNGSWF
RHPDTGQPWSNYTTCVDMDDLEFRKIVNIIYVVGYSVSFAAIVISLIIFLTFRSLRCTRIAIHVQLFSSFAANDLMWIVWYKM
VIGNPIVVQENRFMCQALHVLLQYLMVANYLWMFCEGLHLHLALVVVFVKDDNAMRWFYFIGWFLPAILAGIYALVRSSY
PDETSQCWLSESHTQWILTVPVLLSMLASLGFLINVVRVLLTKLHCNSANPAPIGLRKAVRAALILVPLFGIHHILIPFRPEPNG
PGERVYQVFSALLVSLQGFCVSLLFCFANVDVHAAFKAMARRIRRRATDNGNLTATQTREVV
>ApisCT/DHR2-XP_016661373.1
MYCRSLHVALQFFMVANYMWMFCEGLHLHMALVVVFVNDVNAMRWFYAIGWGVPVALTVLYVSWRSNSEDTAQCW
MHESHCQWVLTVPVFASILTSLMFLMNVMRVLLTKLHRNSTNPAPIGVRKAVRAALILVPLFGIHYILIPYRPKHKTTVETVY
QIFSVVLVSTQVSDARLPSTVRACISIATVKNFAKIIYYYCTNVWIPLRCTSMDIGRDPRKVHLIHRSRSATSIS
>AaegCT/DHR2-AAEL006490
MIETIEEPQRNQTGLFANDNQSNWFPMAMEERPTSGIAASADGIQYDRLKYADPEALMRHLYYACMRNVTNSGSFPAE
QAVTVLRSISASNARHDQQTQLFCPRRFDGWTCWESQPAGTIAQNFCPNFVLGFDASRLAYRICHANGSWFTHPESGRE
WSNYTNCIDVDDMKFRRLVNDLYIGGYTISLVTLIVSLCVFFSFRTLKCTRIRIHINLFTSLALSCAFWILWYKFVVEDPDVTNR
NGNWCIALHILLHYLMLVNYFWMFCEGLQLHLVLVIVFIKDAIAMRWFFTIGWILPVAFVSIYASVRNKYTLDTEHCWMNE
SHAMWLLTIPVCFSLVASLVFLINVVRVLLTKLNSTSPNPAPLGLKKATRATLILIPLFGLQHILLPFRPDKGCELERYYQVVSAV
LISLQGACVSCLFCFANHDVIFAIKCQLSRFFPTLVHHPFRESYNGGQPATQSRDMVV
>TcasCT/DHR2-TC013321
MDVTNGTNCGGKYTRPGYCPEIFDEMLCWPETLGGTTVNQSCPKKMGYDSRRFAYKDCLENGSWFKHPKSGKIWTNYT
TCVDHEDLAFRTHINHLFVIGYSISLAALVISLAIFFTFRTLKCTRIRIHIQLFISFALNNLMWIIWYKEVVPNPFVTIRNELWCQ
ALHLVVHYLMLANYMWMFCEGLHLHLALVVVFVRDAETMKWFFALGWGAPFIIVLIYSVVRIFILKDNYMCWMADSYYS
SWILTAPVCISLLVSLIFLINVLRVILTIMHPNSANPAPMGLRRAARAALILIPLFGLQHILIPFRPDMYDPYEHLYQYVTVVVVT
LQGLCVSCLFCFANQDVHQAIRGFMHRKVYRTTRWSNYHYTGAADSAGVYVVNGSSHCNNVGLLSLKRKSTTTVKL
>AgamCT/DHR2-AGAP001175
MTIREDAAELVRVDLAKANGSAYFGQTGMIDAGGRVGDGQDGQIQFDRLEFESPEDLMRHLYYTCMRSNTSEPPWTTD
GSTVFCPRRFDGWSCWEPTLAGTVAENWCPKFVLGFDPRRLAYRTCHENGSWFVHPHSGREWSNYTNCIDTEDMQLRR
LVNDIYIGGYTVSFLTLIISLCIFHSFRTLKCTRIRIHIHLFTSLALSCLFWIVWYKFVVEDPDVLNANTPLTSICVTVPGNSCAYKQ
SSLQGWCVGLHILLHYLMLVNYFWMFCEGLHLHLVLVIVSIRAHPGDIPRGRLECHRATISQGWCRLLPFSVRFNSIRLIFPLF
HRRHRHHIRTDAFFSVFFRVLLTAGKKVFIKDIVAMRWFVTIGWILPMALASLVFLINVVRVLLTKLSSTSPHPAPLGLRKATR
ATLILIPLFGLQHILLPFRPEKGCSLERYYQIGAALLISLQGLCVSCLFCFANHDVIFAVQCYLSRFFPDLVTHPFLESNGGQPAT
QSRDVVV
>ApisCT/DHR3-XP_016661370
MADYQYGLSNIQDNHILHQVIAERRKMCESVMDDPPMSDSDAWCPGEFDGWTCINRTKAGEVAKFPCPYFILGFDTKRF
GQKTCLPDGSWFKHPDSNKTWSNYTTCVDLEDLKLRNQVNMIYKWGYTVSLAALAVSIFIFFYFRSLTCTRIQIHKNLFISLA
VNNCLWLVWYEAVVDNLPVLMTNGLGCKLLHVLVQYFLVATYFWMFCEGLYLHTLLVVTFLTESKVMPFLHTIGWGIPAL
LVSTYAALRTATPGETLHCWIHESLYSWILSGPVCLSMLANLVFLINIVRLLLVKLHARQITMSSPKHVAPRERAQSFSLRSFK
RNNCSVDSYDKAPASSSRTGKAVRATLILIPLLGLQYIVTPFRPEPGTSWEPVYQVTSAVVASCQGLCVALLFCFCNGEVLSE
MKKRWKHCWINKNGSWNPCMRGTSVSSPQHHPRLLVTEDQQTQRTGSIQVLPRKDSAECLQNVQL
>HhalCT/DHR3-XP_014287171
MIYTREEIDNITAQIKKECFAVAENYTEGLFCPREFDGWTCLNATPAGTVLHFPCPYFQLGFDPKRTAHRPCLENGTWFRHP
ETNKPWSNYTTCVDLEDFEMRNQVNFIYKAGYMISLAALLLSLFIFFYFRSLTCTRIQIHKNFFISLAINNLLWLIWYEAVLDN
HTVIYENGVGCQILHVILQYFLVTTYFWGFCEGLYLYTLLVVTFLTESKVMVCLYLIGWGVPALIVSAYASLRISTNKDTDYCW

IQESIYRWTLIIPVGLSMIANLIFLITIVRVVLTKLHAAQKTTPSNSFKDKNASIRSKRSTVLSDTAFSERTKKAVRATLILIPLIGLQ
YVVMIVRPEQKTTWEYTYELTEAIVASSQGLCVALLFCFCNGEVTAAVRKKWRQCRLSKKRPWNSCSGVTSVSFSRSSFVQ
EMAGVVPPPHIDGLATGNCQL
>ClecCT/DHR3-CLEC013307
MIITQFMFTDGFGCPKEFDGWTCINATSPGAVIHVPCPYFIFGFDPKRLGHRTCLEDGTWFRHPDSNKTWTNYTTCVDTD
DLKMRTQVNLIYKAGYTFSLAALLLSIIIFFYFKSLTCTRIQIHKNLFISLAVNNLLWLVWYEAVVDNIPVLLSNGIGCQILHVLV
QYFMVATYLWMFCEGLYLHTLLVVTFVTESKVMPVLHLIGWGVPALLVTVYASLRVSTKDDTLHCWIHESLYSWSLSGPVC
LSMIANLVFLINIVRLLLTKLRLPRRSTISSQGLPSGRTKKAVRATLILIPLLGLQYIVTPFRPEQGTSWEYPYQVTSALVASCQGL
CVALLFCFFNGEVFLFNLNFLKEENSNFALVLGCSGHEEKVATVQAE
>ClecCRF/DHR1-CLEC025139
MCITKKNFQEIRCFKLMEEERDQKDVCERSWDGLLCWPPSRPGAAYLPCFEELHGIKYDTSQNATRWCHPNGTWANYSN
YTKCSDLGKPAIEFEDGVEVTTMIYSIGYGLSLVALCLAVYIFIYYKELRCLRNTIHTNLMCTYILADFMWILNYSLQISVQTDFV
PCVLLVILLHYFHLTNFFWMFVEGFYLYMLVVKTFTQENIKLRVYLAIGWGNFCLKMNINVLYTMFQIVMDPFQKGCHWM
MPNMTDWIYQTPAIIVLVINLMFLVMIMLVLITKLRSATNVETQQYRKAAKALLVLIPLLGITYILFIVGPSKGPYYANLYEYVR
SILISTQGLMVALFYCFLNTEVQNTVRHHLIRWKEARDIGVRRYTYYCKTRSPNSDIESVR
>ClecCRF/DHR2-CLEC000652
MPTYDEPAENCPWSWDGALCWPSFPPGKNASLPCFEELKGIKYDTSQNASRYCFPNGTWSTYTNYNACQATVPENMVP
EMPWVVGASTIYCIGYSISLVALCVAVWIFIYFKDLRCLRNTIHTNLMCTYILADFLWILNFALQILVPSDFICVVFVLLLKYFILT
NYFWMFVEGLYLYMLVVATFTRENIKLRVYLGIGWGKCFFIGIISTASEELDRNSCSWLTPHWSDWIDQSAALLVLGVNLIF
LFMIMLVLITKLRSSNNVETQQYRKASKALLVLIPLLGVTYIFFLVGPKTDIYEYIRAILLSTQGFSVALFYCFLNTEVQNTFRHHF
LRWKESRDIGHRRYTYSKDWSPNTRTESIR
>HhalCRF/DHR2a-XP_024216523.1
MASNLEVLKNLTTESQFELQSQECATLWSRKEPSGWCRAKFDGALCWGPTAPSHLSTQPCKEVINDVYYDTSKNATKFCD
SNGDWNLTNYDNCQVRDFMVPDAEMTEMSTVIYSVGYTLSLITLSLALFIFIYCKEMRCLRNTIHTNLMFTYVLADFMWIL
SLTVQVSIHTDSISCLVLFTLLHYFLLTNFFWMFVEGLYLYMLVVETFTRENIGLRAYLAIGWGSPIPVIIVWIIARTTVQDPNA
VTIMGVPETMNQCTMMYTSMTDWIYIIPVLVVLVVNLLFLCMIMWVLITKLRSANNAETEQYRKGSKALLVLIPLLGITYILL
IAGPNASVYHNIRALLLSTQGLTVGLLYCFLNTEVQNTLRHRWQRWREERSLPHRTYTKDSSPNTRTESIRLYSKHEVVPYRK
RESTGSETTTMTLVHGNSKISNGPRSSFLQPPSEPV
>HhalCRF/DHR2b-XP_024216523.1
MEDQELTSNFSYHIEREECLQRWSYHYQEGWCPAVFDGALCWGPTGPAVLASQPCREEIHGVLYDTSKNATKYCHESGI
WDNRTDYDQCQERADILATLTADDIEMTTVIYALGYALSLIALSVALFIFIYFKEMRCLRNTIHTNLMLTYVLADFMWILSLTV
SMHTDSVSCLILFTLLHYFILTNFFWMFVEGLYLYMLVVETFTRENIGLRAYLAIGWGLPVAVIIVWVIARFNASDMPEVPPG
TKQCTWMNQSWSDWIYQVPAILVLAINLLFLVRIMWVLITKLRSANNAETEQYRKGSKALLVLIPLLGITYILFIAGPQSAVYS
NIRALLLSTQGLSVGLLYCFLNTEVQNTLRHRWLRWKEERSLATRAYTKDMSPNTRTESIRLYSRHEITPYRKRESTGSESTT
MTLVHSSRLSNGPRSSFLQPPSEPV
>ApisCRF/DHR2-XP_001944842.2
MNITSTNNTCLPEKVLNPGWCPSHWDLLLCWRTSKPGAVVYQACFDELNGMRYDTSQNASRRCKLNGVWENSSDYMN
CRPLDTNNPLYPDPSIVYTSYFYYGGYTISLVALVAAVSIFVYFKDLRCLRNTIHTNLMCTYILSDFTWILTSTLQEWLSASNNA
CVLFTFSLHYFVLTNFFWMFVEGLYLYILVVETFTRENIKLRVYMFIGWGFPLVIMIVWGVSKIITPIEVEERSDSCSWMTPHP
VHDWIYQGPAIIVLIVNLVFLSKIMWVLITKLRSANSAETQQYRKASKALLVLIPLLGVTYILTMVGPTESGTYANYYSYGRATL
LSMQGFMVAIFYCFVNSEVKNTFKHHFIRWNDARNLRTGGSRRFTYSKDWSPNTRSDSVRLARPSIIADTVMNKGKRAST
VSSSTTMTFIVSNGSGGVLLSNACQNRMLNAPPENLV
>ApisCRF/DHR1-XP_008183744.1
MDYFNETWSPNEEAALKCRSWYEWDDRWINGCNASSDSLICWPPTPAGVIVYQPCFQELRGILYDTTKNASRICYENNT
WGLTDFNDCTILGEPKAVTYDEEGTVDTIIYLYIAGHCLSLIMTSLAIFVFCRFKELKCLRNKIHSNLMASYLLAGIMWILNYTN
LTDTGTFKCALLVLPLYYFTMTNYFWAFIEGMYLFILVVDTFFTDRVRLRTYMAIGWGIPLIIIPTWCVTRLLVPTKNDLDLYN
QITYERYCPLMASYVDDWIYQSPIVIVLLINSIFLVKIMRVLITKIRSTKSAETHNYKKATKALLVLIPLLGITFCLDMINPSSTGLL
VNIYKFSKVVIISTQGFTVSLLYCFFNNEVQNTLKYHITRWQTKRKFLASRKKYGRSWLSVKQNNIICDHQLDPKELMPWIPA
NNVRSSSCVSNGTTTSALSNMRLPPDTTIEIPPEQQPLEDSKYVGAKP
>AmelCRF/DHR-LOC413829
MLRIQRELRCIRNNIHTNLMFTYILADFMWILNNVMQVSMQPDVPTCVAFLSLFHYFQLTNYFWMFVEGLYLYLLVVKTFT
GDNIKLRLCLIIGWGIPVLFITMWCIAKSLDQNATSQDIALGKHCPWMVSHAYDWFYQAPAILVLCVNVVFLFMIMWVLIT
KLWSATNVETQQYRKASKALLVLIPLLGVTYVLVLTGPTEGQVANAFSYARAVLLSSQGLFVALFYCFLNTEVRNTVKSHIER
WTTARDLDTERRLYCPPSNPYRKRDSSVSETTTTTVVGLNSTTTFLDKSKKAISEDMNE

>TcasCRF/DHR1-TC007104
MDQGESEVVYARHEDIVRILNRLNETQAELACELKKTLSPPSNGCAVDFDTVLCWPQTAPNSLAVLPCFDQLNGIKYDTRE
NATRLCFANGTWDQYSNYTSCKELSPLEVPEVELTTTIYFIGYTVSLVALLFAVYIFWKFKDLRCLRNTIHMNLMCSYILADFM
WIFVYSLQVPLQTNKAFCIFLIILLHYFHLTNFFWMFVEGLYLYILVVKTFTGENIKPRIYAVIGWGGPILFVLVWGIAKSFTLPL
EDQQAGEMFRSCPWTPHPFDWIYQGPAIAVLIINVIFLCIIMWVLITKLRSANNVETQQYRKAAKALLVLIPLLGVTYILVIVG
PTEGISRRIYDSIRAILLSTQGFTVALFYCFLNAEVKNTVRHHYNSWHTRRTLGSRRTRYSSSKDWSSQARDSMRYGSKRAKL
STLKY
>TcasCRF/DHR2-TC012799
MSWSEPLPQEPEPVDADLWPSSENVLNETEDIKIRLNITLQHCTSLYTNRTTALAETHPDGFCPVTTDGLLCWPPTPINETTY
VKCFAELMNIRYDDTQNATRVCLANGTWTKADYSKCTEIILIPDVETQATIYFVGYVLSLITLSIALGIFTYFKELRCLRNRIHM
NLMWSYMLMYIMWILTLTVLGSKGGTGASIACIFVITLLHYFHISTFFWMFVEGLYLYILVVETLTRENYKLRVYVCIGWGLP
MIFILVWVIVKSFIPAAGDPATCTWFNSHDVDWIFQGPTMLVLLLNLAFLLAIMWVLITKLRSANTVETQQYHKAAKALLVL
MPLLGITYVITIYAPTPDKKSEIIFECVRAVLLSTQPLNEHPPICQSYSVAITGLHCRSILLLLKHGGAEHRPPPLRNVENTTISGPI
QTTFGESQQGLVPQVTHGEYTVREKIFNDSLGTITFGSI
>AgamCRF/DHR1-AGAP005464
MANETMPTTPTAAAIAADDGSGGSPFGEGNVTGISVENPSLVEGLLALAMNASGAERCRLQQQAEELLQDVACPSFFDM
VSCWPRTPPGTLAVLPCFAELKGVQYDSSQNATRFCNVDGTWDNYTDYDRCEHLEQPPPLPSFEPEIELPTLIYFVGYSISLA
ALVLAVAVLVYFKDLRCLRNTIHVNLFLTYIMSSSLWILILSLQITVKLEVAGCIFLVTLFHYFSTTNFFWMLVEGLYLYMLVVQ
TFSGDTLRFRKYAIIGWGGPLIFVGAWAIAKPFFGSVSNLEHPSKLEIECSWMRESHIDWIIQGPSCAVLVINLIFLLRIMWVLI
TKLRSANTVETRQYRKASKALLVLIPLLGITYLIVIYGPVEGVGSHIFAITRAILLSTQGFVVSLLYCFLNSEVRQTLRHHFYRWR
DERNILSGKVNNHHRRPTFSKDNSPRSRTESTRLVLVKECASSVPAKHVLYCRLQVVVAFIHVCLFVFVFWCVCVSLFVFCLY
TLFAPCCHSSLIRSHSTWHTGSLQRFSLLFLNYSKVC
>AgamCRF/DHR2-AGAP005465
MQSCLLSATLQPKPQVKPRTAVRYQTNRDQTIYVPQKFCPTRLWTVQMTGGIISNSIKPSGQRTTPTTLIRLQMDKIQPHA
SGKRLVSVADYDELSSFPPELLGAFENSTEFECLLRAHQDAQSETPSGGCPVDFDRILCWPKTDPGTWAVLPCFEEFKGVHY
DVTQNATRYCHPNGRWDNYSHYAACHHVNEPPPDIVEISSIIYYTGYILSLVALSLAVIVFVYFKDLRCLRNTIHANLFITYILSA
LLWIIILTLQLSSGSSTGMTSCVIFVTLLHYFTLTNFFWMLVEGLYLYMLVVETFSGDNLRFNMYAAIGWGKCSRASLEIECS
WMRESVVDWIFQGPVCAVLIINLVFLIRIMWVLITKLRSANTVETRQYRKASKALLVLIPLLGITYLVVLAAPAEGVVSDIFAIA
RALLLSTQGLSVSLFYCFLNSEVRLALRHRLERWRDERNIRLGQVRQRSRRMEKGPSKPESEPESIHHATESLKGFILPFSSAG
TVPLTYNKRESCASSATTTTLLGPHSYPSAMRGSNGALHMQSIVPRAISPLMQVRRWGEGRKSVTNYYPLRGAGN
>AaegCRF/DHR1-AAEL008292
MNDSSSASVESLLALAGAGGDFAGSGSGDGFPPMILNETLRDLCHQQNDTLFLELSCPPLFDSISCWPRTPPATTAVLPCFS
EFKGVQYDATQNATRYCNIDGTWNSFANYDACKHLELPSSDPTLESFIELPIVIYFVGYTISLLALFCAVTVLVYFKELRCLRNTI
HVNLFVTYIMSSSLWIIILSQQLAAKQGLVDCIFLVTLFHYFSTTNFFWMLVEGLYLYMLVVQTFSGDYLRFWKYSIIGWGGP
LIFVGAWAIAKSFYPYDLMPEHPNKLEIECSWMRESHIDWIIQGPSCAVLVINLIFLLRIMWVLITKLRSANTVETRQYRKASK
ALLVLIPLLGITYLVVIYGPHEGVGSRIFAVTRAVLLSTQGLVVSLLYCFLNYEVRGTLRLHYYRWRDERNIRLGIISKHRRPTISG
TESIRTLEYSVVIMKSLAPPSSRHHHHHNHHNNLGGSN
>AaegCRF/DHR2-AAEL019757
MTEPPPDIVEVTSIIYYSGYIVSLVALSLAVIVFVYFKDLRCLRNTIHANLFITYILSALMWIIILTLQLSGSQSGIASCVILVTLLHYF
TLTNFFWMLVEGLYLYMLVVETFSGDNLRFNMYAAIGWGGPGVFVIMWVIAKGITISGLDQATTLEIDCSWMRESTVDW
IFQGPVCAVLIINLVFLIRIMWVLITKLRSANTVETRQYRKASKALLVLIPLLGITYLVVLAAPSEGVVSDIFAVARALLLSTQGFS
VSLFYCFLNSEVRLALRHRLERWRDERNIRIGQVRQRSRRFTNSGLSKECSPRSRTESFRPLTYSKRESCASSATTTTLLGPPVG
AHGAYPSTMRGSNGALHLHAMAPRAISPLMQGLDENSV
>DmelCRF/DHR1-CG8422
MSDHNHIDSVNASGSDPLLDLHNLDGIGESVELQCLVQEHIEASTYGNDSGHCLTQFDSILCWPRTARGTLAVLQCMDEL
QGIHYDSSKNATRFCHANGTWEKYTNYDACAHLPAPESVPEFEVIVELPTIIYYIGYTLSLVSLSLALIVFAYFKELRCLRNTIHA
NLFFTYIMSALFWILLLSVQISIRSGVGSCIALITLFHFFTLTNFFWMLVEGLYLYMLVVKTFSGDNLRFNIYASIGWGGPALFV
VTWAVAKSLTVTYSTPEKYEINCPWMQETHVDWIYQGPVCAVLIINLTFLLRIMWVLITKLRSANTVETRQYRKAAKALLVLI
PLFGITYLVVLAGPSESGLMGHMFAVLRAVLLSTQGFSVSLFYCFLNSEVRNALRHHISTWRDTRTIQLNQNRRYTTKSFSKG
GGSPRAESMRPLTSYYGRGKRESCVSSATTTTLVGQHAPLSLHRGSNNALHTMPTLAANAMSSGSTLSVMPRAISPLMRQ
GLEENSV
>DmelCRF/DHR2-CG12370
MADDDLRALVDSLDDASQEDLAKVIANFSVDMLQRASALIGAQQGSSGGQLQNRTLQCQQQQQREEEQASLEALASGG
KRILQCPSSFDSVLCWPRTNAGSLAVLPCFEEFKGVHYDTTDNATRFCFPNGTWDHYSDYDRCHQNSGSIPVVPDFSPNVE

LPAIIYAGGYFLSFATLVVALIIFLSFKDLRCLRNTIHANLFLTYITSALLWILTLFLQVITTESSQAGCITLVIMFQYFYLTNFFWM
FVEGLYLYTLVVQTFSSDNISFIIYALIGWGCPAVCILVWSIAKAFAPHLENEHFNGLEIDCAWMRESHIDWIFKVPASLALLV
NLVFLIRIMWVLITKLRSAHTLETRQYYKASKALLVLIPLFGITYLLVLTGPEQGISRNLFEAIRAFLISTQGFFVALFYCFLNSEVR
QTLRHGFTRWRESRNIHRNSSIKNRRHRASKDYSLRSRTESLRLTSTSPIPTGHYE
>DmelGlutR
MKQKNNNGTILVVVMVLSWSRVVDLKSPSNTHTQDSVSVSLPGDIILGGLFPVHEKGEGAPCGPKVYNRGVQRLEAMLY
AIDRVNNDPNILPGITIGVHILDTCSRDTYALNQSLQFVRASLNNLDTSGYECADGSSPQLRKNASSGPVFGVIGGSYSSVSL
QVANLLRLFHIPQVSPASTAKTLSDKTRFDLFARTVPPDTFQSVALVDILKNFNWSYVSTIHSEGSYGEYGIEALHKEATERNV
CIAVAEKVPSAADDKVFDSIISKLQKKPNARGVVLFTRAEDARRILQAAKRANLSQPFHWIASDGWGKQQKLLEGLEDIAEG
AITVELQSEIIADFDRYMMQLTPETNQRNPWFAEYWEDTFNCVLTSLSVKPDTSNSANSTDNKIGVKAKTECDDSYRLSEK
VGYEQESKTQFVVDAVYAFAYALHNLHNDRCNTQSDQTTETRKHLQSESVWYRKISTDTKSQACPDMANYDGKEFYNNY
LLNVSFIDLAGSEVKFDRQGDGLARYDILNYQRQENSSGYQYKVIGKWFNGLQLNSETVVWNKETEQPTSACSLPCEVGMI
KKQQGDTCCWICDSCESFEYVYDEFTCKDCGPGLWPYADKLSCYALDIQYMKWNSLFALIPMAIAIFGIALTSIVIVLFAKNH
DTPLVRASGRELSYTLLFGILVCYCNTFALIAKPTIGSCVLQRFGIGVGFSIIYSALLTKTNRISRIFHSASKSAQRLKYISPQSQVVI
TTSLIAIQVLITMIWMVVEPPGTRFYYPDRREVILKCKIQDMSFLFSQLYNMILITICTIYAIKTRKIPENFNESKFIGFTMYTTCII
WLAFVPIYFGTGNSYEVQTTTLCISISLSASVALVCLYSPKVYILVFHPDKNVRKLTMNSTVYRRSAAAVAQGAPTSSGYSRTH
APGTSALTGGAVGTNASSSTLPTQNSPHLDEASAQTNVAHKTNGEFLPEVGERVEPICHIVNK

==============================
Nuclear receptor sequences
>DmelHR51
MATGRSLLFRVPWYVCLCVCAESAEPGVYWRLRLRLGLPTLAGPHTNTLTLTARTSSCRSIKKERIKASQQANAPPELPLKVS
VDVNIIIAAHSQRRRIGLVRFHQRESEDRPLAVASPRLQINMEPTAMNPKKLHSPQRHCYTPPPAPMHGQAPPPTSTGVAP
PTQPPPPHPAAPNVPNGRLLSWNHSAAAAAAAAAAQAAANSMNHSSAAEGSSMTRIKGQNLGLICVVCGDTSSGKHYG
ILACNGCSGFFKRSVRRKLIYRCQAGTGRCVVDKAHRNQCQACRLKKCLQMGMNKDDDSIDVTNDNEEPHAVSRSDSSFI
MPQFMSPNLYTHQHETVYETSARLLFMAVKWAKNLPSFARLSFRDQVILLEESWSELFLLNAIQWCIPLDPTGCALFSVAE
HCNNLENNANGDTCITKEELAADVRTLHEIFCKYKAVLVDPAEFACLKAIVLFRPETRGLKDPAQIENLQDQAHHTKTQFTA
QIARFGRLLLMLPLLRMISSHKIESIYFQRTIGNTPMEKVLCDMYKN
>Dmeltll
MQSSEGSPDMMDQKYNSVRLSPAASSRILYHVPCKVCRDHSSGKHYGIYACDGCAGFFKRSIRRSRQYVCKSQKQGLCVV
DKTHRNQCRACRLRKCFEVGMNKDAVQHERGPRNSTLRRHMAMYKDAMMGAGEMPQIPAEILMNTAALTGFPGVP
MPMPGLPQRAGHHPAHMAAFQPPPSAAAVLDLSVPRVPHHPVHQGHHGFFSPTAAYMNALATRALPPTPPLMAAEHI
KETAAEHLFKNVNWIKSVRAFTELPMPDQLLLLEESWKEFFILAMAQYLMPMNFAQLLFVYESENANREIMGMVTREVHA
FQEVLNQLCHLNIDSTEYECLRAISLFRKSPPSASSTEDLANSSILTGSGSPNSSASAESRGLLESGKVAAMHNDARSALHNYI
QRTHPSQPMRFQTLLGVVQLMHKVSSFTIEELFFRKTIGDITIVRLISDMYSQRKI
>Dmeldsf
MGTAGDRLLDIPCKVCGDRSSGKHYGIYSCDGCSGFFKRSIHRNRIYTCKATGDLKGRCPVDKTHRNQCRACRLAKCFQSA
MNKDAVQHERGPRKPKLHPQLHHHHHHAAAAAAAAHHAAAAHHHHHHHHHHAHAAAAHHAAVAAAAASGLHHHHH
AMPVSLVTNVSASFNYTQHISTHPPAPAAPPSGFHLTASGAQQGPAPPAGHLHHGGAGHQHATAFHHPGHGHALPAPH
GGVISNPGGNSSAISGSGPGSTLPFPSHLLHHNLIAEAASKLPGITATAVAAVVSSTSTPYASAAQASSPSSNNHNYSSPSPSN
SIQSISSIGSRSGGGEEGLSLGSESPRVNVETETPSPSNSPPLSAGSISPAPTLTTSSGSPQHRQMSRHSLSEATTPPSHASLMI
CASNNNNNNNNNNNNNNGEHKQSSYTSGSPTPTTPTPPPPRSGVGSTCNTASSSSGFLELLLSPDKCQELIQYQVQHNTL
LFPQQLLDSRLLSWEMLQETTARLLFMAVRWVKCLMPFQTLSKNDQHLLLQESWKELFLLNLAQWTIPLDLTPILESPLIRE
RVLQDEATQTEMKTIQEILCRFRQITPDGSEVGCMKAIALFAPETAGLCDVQPVEMLQDQAQCILSDHVRLRYPRQATRFG
RLLLLLPSLRTIRAATIEALFFKETIGNVPIARLLRDMYTMEPAQVDK
>DmelHR78
MDGVKVETFIKSEENRAMPLIGGGSASGGTPLPGGGVGMGAGASATLSVELCLVCGDRASGRHYGAISCEGCKGFFKRSIR
KQLGYQCRGAMNCEVTKHHRNRCQFCRLQKCLASGMRSDSVQHERKPIVDRKEGIIAAAGGSSTSGGGNGSSTYLSGKSG
YQQGRGKGHSVKAESAATPPVHSAPATAFNLNENIFPMGLNFAELTQTLMFATQQQQQQQQHQQSGSYSPDIPKADP
EDDEDDSMDNSSTLCLQLLANSASNNNSQHLNFNAGEAPTALPTTSTMGLIQSSLDMRVIHKGLQILQPIQNQLERNGNL
SVKPECDSEAEDSGTEDAVDAELEHMELDFECGGNRSGGSDFAINEAVFEQDLLTDVQCAFHVQPPTLVHSYLNIHYVCET
GSRIIFLTIHTLRKVPVFEQLEAHTQVKLLRGVWPALMAIALAQCQGQLSVPTIIGQFIQSTRQLADIDKIEPLKISKMANLTRT
LHDFVQELQSLDVTDMEFGLLRLILLFNPTLLQQRKERSLRGYVRRVQLYALSSLRRQGGIGGGEERFNVLVARLLPLSSLDAE
AMEELFFANLVGQMQMDALIPFILMTSNTSGL

>Dmmelusp
MDNCDQDASFRLSHIKEEVKPDISQLNDSNNSSFSPKAESPVPFMQAMSMVHVLPGSNSASSNNNSAGDAQMAQAPNS
AGGSAAAAVQQQYPPNHPLSGSKHLCSICGDRASGKHYGVYSCEGCKGFFKRTVRKDLTYACRENRNCIIDKRQRNRCQY
CRYQKCLTCGMKREAVQEERQRGARNAAGRLSASGGGSSGPGSVGGSSSQGGGGGGGVSGGMGSGNGSDDFMTNSV
SRDFSIERIIEAEQRAETQCGDRALTFLRVGPYSTVQPDYKGAVSALCQVVNKQLFQMVEYARMMPHFAQVPLDDQVILLK
AAWIELLIANVAWCSIVSLDDGGAGGGGGGLGHDGSFERRSPGLQPQQLFLNQSFSYHRNSAIKAGVSAIFDRILSELSVK
MKRLNLDRRELSCLKAIILYNPDIRGIKSRAEIEMCREKVYACLDEHCRLEHPGDDGRFAQLLLRLPALRSISLKCQDHLFLFRIT
SDRPLEELFLEQLEAPPPPGLAMKLE
>DmelKni
MVFNLMNQTCKVCGEPAAGFHFGAFTCEGCKSFFGRSYNNISTISECKNEGKCIIDKKNRTTCKACRLRKCYNVGMSKGGS
RYGRRSNWFKIHCLLQEHEQAAAAAGKAPPLAGGVSVGGAPSASSPVGSPHTPGFGDMAAHLHHHHQQQQQQQVPR
HPHMPLLGYPSYLSDPSAALPFFSMMGGVPHQSPFQLPPHLLFPGYHASAAAAAASAADAAYRQEMYKHRQSVDSVESQ
NRFSPASQPPVVQPTSSARQSPIDVCLEEDVHSVHSHQSSASLLHPIAIRATPTTPTSSSPLSFAAKMQSLSPVSVCSIGGETT
SVVPVHPPTVSAQEGPMDLSMKTSRSSVHSFNDSGSEDQEVEVAPRRKFYQLEAECLTTSSSSSSHSAAHSPNTTTAHAEV
KRQKLGGAEATHFGGFAVAHNAASAMRGIFVCV
>DmelKnrl
MMNQDNPYAMNQTCKVCGEPAAGFHFGAFTCEGCKSFFGRSYNNLSSISDCKNNGECIINKKNRTACKACRLKKCLMVG
MSKSGSRYGRRSNWFKIHCLLQEQQQQAVAAMAAHHNSQQAGGGSSGGSGGGQGMPNGVKGMSGVPPPAAAAAA
LGMLGHPGGYPGLYAVANAGGSSRSKEELMMLGLDGSVEYGSHKHPVVASPSVSSPDSHNSDSSVEVSSVRGNPLLHLG
GKSNSGGSSSGADGSHSGGGGGGGGGVTPGRPPQMRKDLSPFLPLPFPGLASMPVMPPPAFLPPSHLLFPGYHPALYSH
HQGLLKPTPEQQQAAVAAAAVQHLFNSSGAGQRFAPGTSPFANHQQHHKEEDQPAPARSPSTHANNNHLLTNGGAAD
ELTKRFYLDAVLKSQQQSPPPTTKLPPHSKQDYSISALVTPNSESGRERVKSRQNEEDDEARADGIIDGAEHDDEEEDLVVS
MTPPHSPAQQEERTPAGEDPRPSPGQDNPIDLSMKTTGSSLSSKSSSPEIEPETEISSDVEKNDTDDDDEDLKVTPEEEISVR
ETADPEIEEDHSSTTETAKTSIENTHNNNNSISNNNNNNNNNNNNSILSDSEASETIKRKLDELIEASSENGKRLRLEAPVKVAT
SNALDLTTKV
>Dmelea
MNQLCKVCGEPAAGFHFGAFTCEGCKSFFGRTYNNIAAIAGCKHNGDCVINKKNRTACKACRLRKCLLVGMSKSGSRYGR
RSNWFKIHCLLQEQQTTSGLGGGSSVGSGSGGGVSSASLEQLARLQQASNQARQTYQDKTNPCIKSATATTSPRIEGAAV
GTGIGGGASPSFLQAAKLHHQRQLKLDSRLSNTPSDSGASSAGDPNEDGVTSVLGGQIATPSSTNATSLPKLDLRHPNFPAT
SEPDADMQRQRHQELLEIFRSHSEPLYSSFAPFSHLPPVLLAAGVPQLPIFKDQFKAELLFPTTSSPELEEPIDLSFRSRADPAS
PMAHNSNSPSLSEPAAASHCLGESTNFVRKSTPLDLTLVRSQTLTG
>DmelE75B
MEAVQAAAAATSSGGSSGSVPGSGSGSASKLIKTEPIDFEMLHLEENERQQDIEREPSSSNSNSNSNSLTPQRYTHVQVQT
VPPRQPTGLTTPGGTQKVILTPRVEYVQQRATSSTGGGMKHVYSQQQGTAASRSAPPETTALLTTTSGTPQIIITRTLPSNQ
HLSRRHSASPSALHHYQQQQPQRQQSPPPLHHQQQQQQQHVRVIRDGRLYDEATVVVAARRHSVSPPPLHHHSRSAPV
SPVIARRGGAAAYMDQQYQQRQTPPLAPPPPPPPPPPPPPPPQQQQQQYISTGVPPPTAAARKFVVSTSTRHVNVIASN
HFQQQQQQHQAQQHQQQHQQHQQHQQHVIASVSSSSSSSAIGSGGSSSSHIFRTPVVSSSSSSNMHHQQQQQQQQS
SLGNSVMRPPPPPPPPPKVKHASSSSSGNSSSSNTNNSSSSSNGEEPSSSIPDLEFDGTTVLCRVCGDKASGFHYGVHSCEGC
KGFFRRSIQQKIQYRPCTKNQQCSILRINRNRCQYCRLKKCIAVGMSRDAVRFGRVPKREKARILAAMQQSTQNRGQQRAL
ATELDDQPRLLAAVLRAHLETCEFTKEKVSAMRQRARDCPSYSMPTLLACPLNPAPELQSEQEFSQRFAHVIRGVIDFAGMI
PGFQLLTQDDKFTLLKAGLFDALFVRLICMFDSSINSIICLNGQVMRRDAIQNGANARFLVDSTFNFAERMNSMNLTDAEIG
LFCAIVLITPDRPGLRNLELIEKMYSRLKGCLQYIVAQNRPDQPEFLAKLLETMPDLRTLSTLHTEKLVVFRTEHKELLRQQM
WSMEDGNNSDGQQNKSPSGSWADAMDVEAAKSPLGSVSSTESADLDYGSPSSSQPQGVSLPSPPQQQPSALASSAPLL
AATLSGGCPLRNRANSGSSGDSGAAEMDIVGSHAHLTQNGLTITPIVRHQQQQQQQQQIGILNNAHSRNLNGGHAMCQ
QQQQHPQLHHHLTAGAARYRKLDSPTDSGIESGNEKNECKAVSSGGSSSCSSPRSSVDDALDCSDAAANHNQVVQHPQL
SVVSVSPVRSPQPSTSSHLKRQIVEDMPVLKRVLQAPPLYDTNSLMDEAYKPHKKFRALRHREFETAEADASSSTSGSNSLS
AGSPRQSPVPNSVATPPPSAASAAAGNPAQSQLHMHLTRSSPKASMASSHSVLAKSLMAEPRMTPEQMKRSDIIQNYLK
RENSTAASSTTNGVGNRSPSSSSTPPPSAVQNQQRWGSSSVITTTCQQRQQSVSPHSNGSSSSSSSSSSSSSSSSSTSSNCSS
SSASSCQYFQSPHSTSNGTSAPASSSSGSNSATPLLELQVDIADSAQPLNLSKKSPTPPPSKLHALVAAANAVQRYPTLSADV
TVTASNGGPPSAAASPAPSSSPPASVGSPNPGLSAAVHKVMLEA
>DmelE78C
MDVYQIELEEQAQIRSKLLVETCVKHSSSEQQQLQVKQEDLIKDFTRDEEEQPSEEEAEEEDNEEDEEEEGEEEEEDEDEEAL
LPVVNFNANSDFNLHFFDTPEDSSTQGAYSEANSLESEQEEEKQTQQHQQQKQHHRDLEDCLSAIEADPLQLLHCDDFYR
TSALAESVAASLSPQQQQQRQHTHQQQQQQQQQQHPGQQQHQLNCTLSNGGGALYTISSVHQFGPASNHNTSSSSP

SSSAAHSSPDSGCSSASSSGSSRSCGSSSASSSSSAVSSTISSGRSSNNSVVNPAATSSSVAHLNKEQQQQPLPTTQLQQQQ
QHQQQLQHPQQQQSFGLADSSSSNGSSNNNNGVSSKSFVPCKVCGDKASGYHYGVTSCEGCKGFFRRSIQKQIEYRCLRD
GKCLVIRLNRNRCQYCRFKKCLSAGMSRDSVRYGRVPKRSRELNGAAASSAAAGAPASLNVDDSTSSTLHPSHLQQQQQQ
HLLQQQQQQQHQPQLQQHHQLQQQPHVSGVRVKTPSTPQTPQMCSIASSPSELGGCNSANNNNNNNNNNSSSGNASG
GSGVSVGVVVVGGHQQLVGGSMVGMAGMGTDAHQVGMCHDGLAGTANELTVYDVIMCVSQAHRLNCSYTEELTREL
MRRPVTVPQNGIASTVAESLEFQKIWLWQQFSARVTPGVQRIVEFAKRVPGFCDFTQDDQLILIKLGFFEVWLTHVARLIN
EATLTLDDGAYLTRQQLEILYDSDFVNALLNFANTLNAYGLSDTEIGLFSAMVLLASDRAGLSEPKVIGRARELVAEALRVQIL
RSRAGSPQALQLMPALEAKIPELRSLGAKHFSHLDWLRMNWTKLRLPPLFAEIFDIPKADDEL
>DmelHR3
MASLLGSAPPAQILANQPIIVKIEPTQSFHIVDEGDTRVLSLPLSDADKLGASWIDLKDIAGLQAGGGATLLDVCFEQANEDG
TIIATVQPDLENELEAELKAEGEPEDETEPEPPAPKRLATTRPAQSRPQQQQQQQQQQVKFLSDPPALARSSSFSSLSSFSSIS
NISSVCKNMASNTSQEGSLKRTKERTPPPMPPLTTHKPAATTTATSATSAAAATSAATASATATSARENSREHSSSSSGSNG
AMTAQIEIIPCKVCGDKSSGVHYGVITCEGCKGFFRRSQSSVVNYQCPRNKQCVVDRVNRNRCQYCRLQKCLKLGMSRDA
VKFGRMSKKQREKVEDEVRFHRAQMRAQSDAAPDSSVYDTQTPSSSDQLHHNNYNSGGYSNNEVGYGSPYGYSASVTP
QQTMQYDISADYVDSTTYEPRSTIIDPEFISHADGDINDVLIKTLAEAHANTNTKLEAVHDMFRKQPDVSRILYYKNLGQEEL
WLDCAEKLTQMIQNIIEFAKLIPGFMRLSQDDQILLLKTGSFELAIVRMSRLLDLSQNAVLYGDVMLPQEAFYTSDSEEMRL
VSRIFQTAKSIAELKLTETELALYQSLVLLWPERNGVRGNTEIQRLFNLSMNAIRQELETNHAPLKGDVTVLDTLLNNIPNFRD
ISILHMESLSKFKLQHPNVVFPALYKELFSIDSQQDLT
>DmelEcR
MLTTSGQQQSKQKLSTLPSHILLQQQLAASAGPSSSVSLSPSSSAALTLHVASANGGARETTSAAAVKDKLRPTPTAIKIEPM
PDVISVGTVAGGSSVATVVAPAATTTSNKPNSTAAPSTSAAAANGHLVLVPNKRPRLDVTEDWMSTPSPGSVPSSAPPLSP
SPGSQNHSYNMSNGYASPMSAGSYDPYSPTGKTGRDDLSPSSSLNGYSANESCDAKKSKKGPAPRVQEELCLVCGDRASG
YHYNALTCEGCKGFFRRSVTKSAVYCCKFGRACEMDMYMRRKCQECRLKKCLAVGMRPECVVPENQCAMKRREKKAQK
EKDKMTTSPSSQHGGNGSLASGGGQDFVKKEILDLMTCEPPQHATIPLLPDEILAKCQARNIPSLTYNQLAVIYKLIWYQDG
YEQPSEEDLRRIMSQPDENESQTDVSFRHITEITILTVQLIVEFAKGLPAFTKIPQEDQITLLKACSSEVMMLRMARRYDHSSD
SIFFANNRSYTRDSYKMAGMADNIEDLLHFCRQMFSMKVDNVEYALLTAIVIFSDRPGLEKAQLVEAIQSYYIDTLRIYILNR
HCGDSMSLVFYAKLLSILTELRTLGNQNAEMCFSLKLKNRKLPKFLEEIWDVHAIPPSVQSHLQITQEENERLERAERMRASV
GGAITAGIDCDSASTSAAAAAAQHQPQPQPQPQPSSLTQNDSQHQTQPQLQPQLPPQLQGQLQPQLQPQLQTQLQPQI
QPQPQLLPVSAPVPASVTAPGSLSAVSTSSEYMGGSAAIGPITPATTSSITAAVTASSTTSAVPMGNGVGVGVGVGGNVS
MYANAQTAMALMGVALHSHQEQLIGGVAVKSEHSTTA
>DmelHR96
MSPPKNCAVCGDKALGYNFNAVTCESCKAFFRRNALAKKQFTCPFNQNCDITVVTRRFCQKCRLRKCLDIGMKSENIMSEE
DKLIKRRKIETNRAKRRLMENGTDACDADGGEERDHKAPADSSSSNLDHYSGSQDSQSCGSADSGANGCSGRQASSPGT
QVNPLQMTAEKIVDQIVSDPDRASQAINRLMRTQKEAISVMEKVISSQKDALRLVSHLIDYPGDALKIISKFMNSPFNALTVF
TKFMSSPTDGVEIISKIVDSPADVVEFMQNLMHSPEDAIDIMNKFMNTPAEALRILNRILSGGGANAAQQTADRKPLLDKE
PAVKPAAPAERADTVIQSMLGNSPPISPHDAAVDLQYHSPGVGEQPSTSSSHPLPYIANSPDFDLKTFMQTNYNDEPSLDS
DFSINSIESVLSEVIRIEYQAFNSIQQAASRVKEEMSYGTQSTYGGCNSAANNSQPHLQQPICAPSTQQLDRELNEAEQMKL
RELRLASEALYDPVDEDLSALMMGDDRIKPDDTRHNPKLLQLINLTAVAIKRLIKMAKKITAFRDMCQEDQVALLKGGCTE
MMIMRSVMIYDDDRAAWKVPHTKENMGNIRTDLLKFAEGNIYEEHQKFITTFDEKWRMDENIILIMCAIVLFTSARSRVIH
KDVIRLEQNSYYYLLRRYLESVYSGCEARNAFIKLIQKISDVERLNKFIINVYLNVNPSQVEPLLREIFDLKNH
>DmelHNF4
MMKHPQDLSVTDDQQLMKVNKVEKMEQELHDPESESHIMHADALASAYPAASQPHSPIGLALSPNGGGLGLSNSSNQS
SENFALCNGNGNAGSAGGGSASSGSNNNNSMFSPNNNLSGSGSGTNSSQQQLQQQQQQQSPTVCAICGDRATGKHYG
ASSCDGCKGFFRRSVRKNHQYTCRFARNCVVDKDKRNQCRYCRLRKCFKAGMKKEAVQNERDRISCRRTSNDDPDPGNG
LSVISLVKAENESRQSKAGAAMEPNINEDLSNKQFASINDVCESMKQQLLTLVEWAKQIPAFNELQLDDQVALLRAHAGEH
LLLGLSRRSMHLKDVLLLSNNCVITRHCPDPLVSPNLDISRIGARIIDELVTVMKDVGIDDTEFACIKALVFFDPNAKGLNEPH
RIKSLRHQILNNLEDYISDRQYESRGRFGEILLILPVLQSITWQMIEQIQFAKIFGVAHIDSLLQEMLLGGELADNPLPLSPPNQ
SNDYQSPTHTGNMEGGNQVNSSLDSLATSGGPGSHSLDLEVQHIQALIEANSADDSFRAYAASTAAAAAAAVSSSSSAPA
SVAPASISPPLNSPKSQHQHQQHATHQQQQESSYLDMPVKHYNGSRSGPLPTQHSPQRMHPYQRAVASPVEVSSGGGG
LGLRNPADITLNEYNRSEGSSAEELLRRTPLKIRAPEMLTAPAGYGTEPCRMTLKQEPETGY
>DmelHR83
MSNFSACAVCGDQSSGKHYGVSCCDGCSCFFKRSVRRGSSYACIALVGNCVVDKARRNWCPSCRFQRCLAVGMNAAAV
QEERGPRNQQVALYRTGRRQAPPSQAAPSPTPHSQALHFQILAQILVTCLRQAKANEQFALLDRCQQDAIFQVVWSEIFVL

RASHWSLDISAMIDGCGDEQLKRLICEAHQLRADVLELNFMESLILCRKELAINAEYAVILGSHSKAALISLARYTLQQSNYLR
FGQLLLGLRQLCLRRFDCALSCMFRSVVRDILKTL
>Dmelsvp
MCASPSTAPGFFNPRPQSGAELSAFDIGLSRSMGLGVPPHSAWHEPPASLGGHLHAASAGPGTTTGSVATGGGGTTPSSV
ASQQSAVIKQDLSCPSLNQAGSGHHPGIKEDLSSSLPSANGGSAGGHHSGSGSGSGSGVNPGHGSDMLPLIKGHGQDML
TSIKGQPTGCGSTTPSSQANSSHSQSSNSGSQIDSKQNIECVVCGDKSSGKHYGQFTCEGCKSFFKRSVRRNLTYSCRGSRN
CPIDQHHRNQCQYCRLKKCLKMGMRREAVQRGRVPPTQPGLAGMHGQYQIANGDPMGIAGFNGHSYLSSYISLLLRAEP
YPTSRYGQCMQPNNIMGIDNICELAARLLFSAVEWAKNIPFFPELQVTDQVALLRLVWSELFVLNASQCSMPLHVAPLLAA
AGLHASPMAADRVVAFMDHIRIFQEQVEKLKALHVDSAEYSCLKAIVLFTTGKLLDILYKDVPALLTKVSALLGKGSTASNDD
VLAVVRDHLDELNRQEQESQAQQQAPLHLAAFMNCVAGVEAAVQQAEQAQVPTSSASASVSAPLVPSAGSAFSSCQAK
SAGSEMDLLASLYAQAQATPPSSGGGDASGHNNSSGLGASLPTQSQSGSSSRNLTASPLSTSLATAPAPASASAPAPVPTSS
VAQVPVPAPVPVTSSASSSSLGGGAYQTPSAAAAAAAMFHYQTPPRAAFGSAFDMFHHSTPFGVGVGHAHALAHSSGS
GSASFGSPSYRYSPYSLAGSRWQL
>DmelERR
MSDGVSILHIKQEVDTPSASCFSPSSKSTATQSGTNGLKSSPSVSPERQLCSSTTSLSCDLHNVSLSNDGDSLKGSGTSGGNG
GGGGGGTSGGNATNASAGAGSGSVRDELRRLCLVCGDVASGFHYGVASCEACKAFFKRTIQGNIEYTCPANNECEINKRR
RKACQACRFQKCLLMGMLKEGVRLDRVRGGRQKYRRNPVSNSYQTMQLLYQSNTTSLCDVKILEVLNSYEPDALSVQTPP
PQVHTTSITNDEASSSSGSIKLESSVVTPNGTCIFQNNNNNDPNEILSVLSDIYDKELVSVIGWAKQIPGFIDLPLNDQMKLLQ
VSWAEILTLQLTFRSLPFNGKLCFATDVWMDEHLAKECGYTEFYYHCVQIAQRMERISPRREEYYLLKALLLANCDILLDDQS
SLRAFRDTILNSLNDVVYLLRHSSAVSHQQQLLLLLPSLRQADDILRRFWRGIARDEVITMKKLFLEMLEPLAR
>DmelHR38
MMRDRLASLIVVKQEGGSNTSISHHQATAIKCEASLYTESSLFQEINNNSCYRQNLNAPTHQQSHTSHLQHAQQHQTHQ
QHPLLPPPLPTLPLIYPCRNLFPDGCDINHLACCSSSNSNSNCNSDSNSTSSSPGNSHFFANGNTCAAALTPAPPATEPRKIKP
LGAGKLKVGKTDSNSDSNSNCDSRAAAAASTSATSATSATTLAATAAATAAAAEAGGAASAAAAAKISQVRLTNQATTSM
LLLQPNSSFSSLSPFDNFSTQTASTTTTTSASAAGHHQHHNHLLHQQHHNQQQQQQQQQQQQQQQQQQQEHLQQQ
HQQQLVSPQQHLLKSETLLSHEEDQLISNLTDSSVVSHSELFSDLFFPSDSNNSLLSPTTSGYPDNPAEDLTSSIENLTKLTCLR
DKRLSSIPEQQLSSEQEQQLCLLSLRSSSDPAIALHAQQQQQQQQQQQQQQQQHQQQQQHLQLQLISPIGGPLSCGSSL
PSFQETYSLKYNSSSGSSPQQASSSSTAAPTPTDQVLTLKMDEDCFPPLSGGWSASPPAPSQLQQLHTLQSQAQMSHPNS
SNNSSNNAGNSHNNSGGYNYHGHFNAINASANLSPSSSASSLYEYNGVSAADNFYGQQQQQQQQSYQQHNYNSHNGE
RYSLPTFPTISELAAATAAVEAAAAATVSSPSVGGPPPVRRASLPVQRTVSPAGSTAQSPKLAKITLNQRHSHAHAHALQLN
SAPNSAASSPASADLQAGRLLQAPSQLCAVCGDTAACQHYGVRTCEGCKGFFKRTVQKGSKYVCLADKNCPVDKRRRNRC
QFCRFQKCLVVGMVKEVVRTDSLKGRRGRLPSKPKSPQESPPSPPISLITALVRSHVDTTPDPSCLDYSHYEEQSMSEADKV
QQFYQLLTSSVDVIKQFAEKIPGYFDLLPEDQELLFQSASLELFVLRLAYRARIDDTKLIFCNGTVLHRTQCLRSFGEWLNDIM
EFSRSLHNLEIDISAFACLCALTLITERHGLREPKKVEQLQMKIIGSLRDHVTYNAEAQKKQHYFSRLLGKLPELRSLSVQGLQR
IFYLKLEDLVPAPALIENMFVTTLPF
>DmelFtz
MDTFNVPMLAESSNTNYATEATSNHHHLQHQHQQQHSHQQQQQQQLLMPHHHKDQMLAAGSSPMLPFYSHLQLQQ
KDATATIGPAAAAAAVEAATTSANADNFSSLQTIDASQLDGGISLSGLCDRFFVASPNPHSNSNMTLMGTATAATTTTTNN
NNNNNTNNNNNNNVEAKTVRPSNGNSVIIESVTMPSFANILFPTHRSANECIDPALLQKNPQNPNGNNSSIIVPPVEYHQL
KPLEVNSSTSVSTSNFLSSTTAQLLDFEVQVGKDDGHISTTTTTGPGSGSASGSGSGSGSGSIARTIGTATPTTTTSMSNT
ANPTRSSLHSIEELAASSCAPRAASPNSNHTSSASTTPQQQQQQQHHMQSGNHSGSNLSSDDESMSEDEFGLEIDDNGG
YQDTTSSHSQQSGGGGGGGGGNLLNGSSGGSSAGGGYMLLPQAASSSGNNGNPNAGHMSSGSVGNGSGGAGNGGA
GGNSGPGNPMGGTSATPGHGGEVIDFKHLFEELCPVCGDKVSGYHYGLLTCESCKGFFKRTVQNKKVYTCVAERSCHIDKT
QRKRCPYCRFQKCLEVGMKLEAVRADRMRGGRNKFGPMYKRDRARKLQVMRQRQLALQALRNSMGPDIKPTPISPGYQ
QAYPNMNIKQEIQIPQVSSLTQSPDSSPSPIAIALGQVNASTGGVIATPMNAGTGGSGGGGLNGPSSVGNGNSSNGSSNG
NNNSSTGNGTSGGGGGNNAGGGGGGTNSNDGLHRNGGNGNSSCHEAGIGSLQNTADSKLCFDSGTHPSSTADALIEPL
RVSPMIREFVQSIDDREWQTQLFALLQKQTYNQVEVDLFELMCKVLDQNLFSQVDWARNTVFFKDLKVDDQMKLLQHS
WSDMLVLDHLHHRIHNGLPDETQLNNGQVFNLMSLGLLGVPQLGDYFNELQNKLQDLKFDMGDYVCMKFLILLNPSVR
GIVNRKTVSEGHDNVQAALLDYTLTCYPSVNDKFRGLVNILPEIHAMAVRGEDHLYTKHCAGSAPTQTLLMEMLHAKRKG
>DmelHR39
MPNMSSIKAEQQSGPLGGSSGYQVPVNMCTTTVANTTTTLGSSAGGATGSRHNVSVTNIKCELDELPSPNGNMVPVIAN
YVHGSLRIPLSGHSNHRESDSEEELASIENLKVRRRTAADKNGPRPMSWEGELSDTEVNGGEELMEMEPTIKSEVVPAVAP
PQPVCALQPIKTELENIAGEMQIQEKCYPQSNTQHHAATKLKVAPTQSDPINLKFEPPLGDNSPLLAARSKSSSGGHLPLPT
NPSPDSAIHSVYTHSSPSQSPLTSRHAPYTPSLSRNNSDASHSSCYSYSSEFSPTHSPIQARHAPPAGTLYGNHHGIYRQMKV

EASSTVPSSGQEAQNLSMDSASSNLDTVGLGSSHPASPAGISRQQLINSPCPICGDKISGFHYGIFSCESCKGFFKRTVQNRK
NYVCVRGGPCQVSISTRKKCPACRFEKCLQKGMKLEAIREDRTRGGRSTYQCSYTLPNSMLSPLLSPDQAAAAAAAAAVAS
QQQPHQRLHQLNGFGGVPIPCSTSLPASPSLAGTSVKSEEMAETGKQSLRTGSVPPLLQEIMDVEHLWQYTDAELARINQ
PLSAFASGSSSSSSSSGTSSGAHAQLTNPLLASAGLSSNGENANPDLIAHLCNVADHRLYKIVKWCKSLPLFKNISIDDQICLLI
NSWCELLLFSCCFRSIDTPGEIKMSQGRKITLSQAKSNGLQVSLCEL
>DmelHR4
MTPLQRDQLNHQQFVHHYLQSQPVNNKIDSFDIKHIRKTQNPTTNPKFRTEDRFKTSSKSKITNPESGNTQLHFHLSETPDL
SASTSLLPSAFVASSAALVNVTCQAEEGQSSAGSHYTVHRGNDAGISFAALDFRFIRRAAQMNAQQPLILEPGSKASSVVAA
AAELEAATTTAALATSSATEASAADSYPAVQQNGGHEEQRLNGNHGCRREQPDDRTDQPEQLDQQDEPEPVKDVANKL
TIIKTPLNKTLLKKCNSNGSLQMPHANNGSSININNRNNTNNTNNNNSNNNNNAASNDVCDSGLQSGNSNEQEPFANN
DRHHHIYHHHHHHYHHHHGGDKSEASGDVASSSSADPNSQVKDEPTAQDSQDNQAAGAAEAGGATAVAATCSPSKCN
KCNSHNNNSNCNNNNNTISNNNNNTSSNTNTNSNSNNNANNNNNNNNNNHYNNYNNYYKKKSRMPPICRIMTLSRG
PYSELDKMSLFQDLKLKRRKIDSRCSSDGESIADTSTSSPDLLAPMSPKLCDSGSAGASLGASLPLPLALPLPMALPLPMSLPL
PLTAASSAVTVSLAAVVAAVAETGGAGAGGAGTAVTASGAGPCVSTSSTTAAAATSSTSSLSSSSSSSSTSSSTSSASPTAG
ASSTATCPASSSSSSGNGSGGKSGSIKQEHTEIHSSSSAISAAAASTVMSPPPAEATRSSPATPEGGGPAGDGSGATGGGNT
SGGSTAGVAINEHQNNGNGSGGSSRASPDSLEEKPSTTTTTGRPTLTPTNGVLSSASAGTGISTGSSAKLSEAGMSVIRSVK
EERLLNVSSKMLVFHQQREQETKAVAAAAAAAAAGHVTVLVTPSRIKSEPPPPASPSSTSSTQRERERERDRERDRERERER
DRDREREREQSISSSQQHLSRVSASPPTQLSHGSLGPNIVQTHHLHQQLTQPLTLRKSSPPTEHLLSQSMQHLTQQQAIHLH
HLLGQQQQQQQASHPQQQQQQQHSPHSLVRVKKEPNVGQRHLSPHHQQQSPLLQHHQQQQQQQQQQQQHLHQQ
QQQQQHHQQQPQALALMHPASLALRNSNRDAAILFRVKSEVHQQVAAGLPHLMQSAGGAAAAAAAAVAAQRMVCFS
NARINGVKPEVIGGPLGNLRPVGVGGGNGSGSVQCPSPHPSSSSSSSQLSPQTPSQTPPRGTPTVIMGESCGVRTMVWGY
EPPPPSAGQSHGQHPQQQQQSPHHQPQQQQQQQQQQSQQQQQQQQQQSLGQQQHCLSSPSAGSLTPSSSSGGGS
VSGGGVGGPLTPSSVAPQNNEEAAQLLLSLGQTRIQDMRSRPHPFRTPHALNMERLWAGDYSQLPPGQLQALNLSAQQ
QQWGSSNSTGLGGVGGGMGGRNLEAPHEPTDEDEQPLVCMICEDKATGLHYGIITCEGCKGFFKRTVQNRRVYTCVAD
GTCEITKAQRNRCQYCRFKKCIEQGMVLQAVREDRMPGGRNSGAVYNLYKVKYKKHKKTNQKQQQQAAQQQQQQAA
AQQQHQQQQQHQQHQQHQQQQLHSPLHHHHHQGHQSHHAQQQHHPQLSPHHLLSPQQQQLAAAVAAAAQHQQ
QQQQQQQQQQQAKLMGGVVDMKPMFLGPALKPELLQAPPMHSPAQQQQQQQQQQQQQQQASPHLSLSSPHQQQ
QQQQGQHQNHHQQQGGGGGGAGGGAQLPPHLVNGTILKTALTNPSEIVHLRHRLDSAVSSSKDRQISYEHALGMIQTLI
DCDAMEDIATLPHFSEFLEDKSEISEKLCNIGDSIVHKLVSWTKKLPFYLEIPVEIHTKLLTDKWHEILILTTAAYQALHGKRRGE
GGGSRHGSPASTPLSTPTGTPLSTPIPSPAQPLHKDDPEFVSEVNSHLSTLQTCLTTLMGQPIAMEQLKLDVGHMVDKMT
QITIMFRRIKLKMEEYVCLKVYILLNKAEVELESIQERYVQVLRSYLQNSSPQNPQARLSELLSHIPEIQAAASLLLESKMFYVPF
VLNSASIR
>Ph516230-ERR
MDLERRRGEYNMSGDGAPILAISHIKKEVDGSGFLACSPTNSSTSTNIYSPMKTELPNQMDYGNLQEMNCYDVTHSRSPES
PDQQFCSSTTQFVGEASVNNAEGEIREDDLPRRLCLVCGDVASGFHYGVASCEACKAFFKRTIQGNIEYTCPAANDCEINKR
RRKACQACRFQKCLRMGMLKEGVRLDRVRGGRQKYRRNTDTPYQIHSMLPLKPYLPSLEDNKILEALSLNEPDISVMAQDI
SNADPAVRTLNILSDLYDQELVKIITWAKHIPGFLDLTLNDQMRLLQSTWAEILTLTVTYRSLPGTGELKFAADFSFDEKMAR
ECGAMDIYQHCMHIVERQEKLNITKEEYYIIKALVLANSDVRIDDLPPLKKLRDNILSALADCVAVLRPNTTSLHMNHLLLCLP
ALRQADYINRRFWTAVHREGKVYMNKLFIEMLESYIR
>Ph596250-dsf
MRINRDRLLDIPCKVCGDRSSGKHYGIYSCDGCSGFFKRSIHRNRVYTCKAQGDLKGRCPIDKTHRNQCRACRLNKCFIAA
MNKDAVQHERGPRKPKHNITSKENNHHHHHHHHHHPVLMHHSNPPLSNSAEQPSAKQPPPPPPPPGIVFPQPINPHMHY
LQSPIKTSQELIPASENFVPNITPIPSGPPSAAAMFLAHQPPPPGLLQILMTAEKCQELLWNTKLSTTERVGVPQSSPLALQPL
SPSWEVLQETTARLLFTAVRWVRSLGPFQTLSRHDQLLLLQESWKELFLLYLAQWSIPWDLTTLLNSSKARDRLPQDEITAN
EIKTIQELIGRFRQLSPDLSECGCMKAVILFTPETAGLCDVQPVEMLQDQAQCILGDYIRNRYPRQPTRFGRLLLILPNLRSIRQ
LTVEQLFFKETIGEIPIQRLLGDMYHME*
>Ph494010-PNR
MDHKLEALCKVCGDKASGKHYGVISCDGCRGFFKRSIRRGLAYHCKESNSCIVDVTRRNQCQACRFKKCLSVNMKRDAVQ
HERSPRTSFPIHCTGLSSTGRRNNNNNNSNTYSYSGSFLTAATSGPFLDLPTYHFVYPQPSFPIHFDSLVSKQNVWDLTISSQ
QGHPENVYESAAKLLFLTVKWARSIPSFLQLTFHDQSILLENTWNELFILSAAQWTLPVDEEYLVRVTSLPNNKAKEKFEREV
KNFKKIITKFNNLNVDYTEYACLKALTLFKAETSELKDRLQVEMLQDQTHIMLHDYCTSQDSHKARFGKLLMLLPSVNGLSK
DFIEELLFRKTIGIYK*
>Ph477600-HNF4

MVTESELQMQGTQPGLQGSLQNFNAPTGITQNCAICGDRATGKHYGAASCDGCKGFFRRSVRKNHQYTCRFSRNCVVD
KDKRNQCRYCRLRKCFKAGMKKEAVQNERDRISCRRPSYDETNQNNGLSVTSLLNAEMLSRQQNGSPVNDYDLSNKQIA
HINDICESMRQQLLILVEWAKYIPAFTELHLDDQVALLRAHAGEHLLLGLARRSLHLKDILLLGNNCIITRYCSENARSPDVDIS
RVGIRVMDELVKPLTEVQIDDTEFACLKAIVFFDPNAKGLSDTTRIKHLRYQIQINLEDYISDRQYDTRGRFGEILLILPALQSIT
WQMIEQIQFAKLFGVARIDNLLQEMLLGGKHLSN*
>Ph475840-Ftz
MRGGRNKFGPMYKRDRARKLQMMRQRQIAVQTIRGHPLTDGQVAFSYGPPSHFENLHNIKQEIQIPQVSSLTSSPDSSPS
PITVSFGQTTSANMIATTQSGQPPVLQIVGTGGNPTSNNLVPSSDHKLWGTANSTTPSPNSISPKAFPFDGVSLPHGSSGG
PGNSSSSSCNKISPMIRDFISSVDDREWQNSLFGLLQNQTYNQCEVDLFELMCKVLDQNLFSQVDWARNSIFFKDLKVDD
QMKLLQHSWSDMLVLDHLHQRMHNNLPDETTLPNGQKFDLLCLGLLGVPTLADHFTDLTNKLQDLKFDVSDYICVKFLLL
LNPDVRGISNRKHVQEGHEQVLQALLDYTLTIYPHIQDKFTKLQQVLPEIHQMASRGEDHLYHKHCSGGAPTQTLLMEML
HAKRK*
>Phknrl1
MESESSSPGIGSALGRWWGQSPQSSSPSTDVMNQLCRVCGEPAAGFHFGAFTCEGCKSFFGRTYNNLGSITECKNNGECV
INKKNRTACKACRLRKCLLVGMSKSGSRYGRRSNWFKIHCLLQEQQQAGAHPLKDKSSAIALWGESYKNLTTPPHFDTDSK
VSPTEESILLNNNNVTKDRERSPNPVLPSYSAEAAAALWAARHSLFPMQLPHHPMPSIPMPFMPPFANFSPPTHSQSQRN
ILLPFMQTLNSSSSSSSPPVRPNHHPPMMMSSSSSRSSEGSICSQSPSPNRNNQRNNDDVSPSTIRFTEKTETTVEKRSSLSS
SSSPPSDKSPTNNNRLSAVNSIKVKNPPLLLDNPIEITNERVDDVASSKKIQTEEHSLALLRSLGPVQDQPMDLSVRVIEIKSRR
KSSKVNHRIAKNEYYHHHHRDDDKNNESSGEEGDAVNDVKNDVLDSIENNDENFNKRKNPLDLTKSTKLEI*
>Phknrl2
MNQQCKVCGEPAAGFHFGAFTCEGCKSFFGRSYNNLNSITECKNNGECIINKKNRTACKSCRLRKCLLVGMSKSGSRYGRR
SNWFKIHCLLQEQQQAAVAQMKGKNPSTTNPLENPLPLLSPLAFPHPNLGSFLRPNNSSSPSDFYLPVPFHSMFMQPPPP
MSSPFLLPPSLLPFPVPPTKHNDYSQRLSPPDHVQNVHKRNCLTPEYDEENTRTDSPPSRQSPTNNNNNNDYSSKCININD
DNSDDNSFGKNKESDKEMMMTVRSKRFYLDAILGLKTSSEKKIESVNEKKINDDVINNDDDNDDEIEKLTIKRYDDEQDNPI
DLSVKSSKFKKDFFTKENNRRKKHVEEEEEEEENVVEEKKKIFNNNNRDDVKPIPLDLTV
>PhEcR
MFRGGASAMKPIGNCEEDLVLTQVKSEPRVHSPCEKDMTPSASSNQSNSLFSTISSSSKRPRTDDWLSPPSPGPPMGSAPL
TPSPGPHNQYTVISNGYSSPMSSGSYDPYSPNGKLGREDLSPPGSLNGYSVDSSDSKKKKGPTPRQQEELCLVCGDRASGY
HYNALTCEGCKGFFRRSITKNAVYQCKYGDSCEIDMYMRRKCQECRLKKCLSVGMRPECVVPEIQCEVKRREKKAQREKGK
PSSTTNGSPELIMADTPAIKTEPNTTQSHNEKSSANGVKPISPEQEELIHRLVYFQNEYEQPSDEDLKRISNAPSEGEDQSDL
NFRHITEITILTVQLIVEFAKRLPGFDKLLREDQIALLKACSSEVMMLRMARRYDVGSDSILFANNQPYTRDSYSLAGMGETV
DDLLRFCRQMYGMKVDNAEYALLTAIVIFSERPSLIEGWKVEKIQEIYLEALKVYVDNRRKPRSGTIFAKLLSVLTELRTLGNLN
SEMCFSLKLKNKKLPPFLAEIWDVIP*
>Ph460980-HR96
MNSRASSRELNDAERAKLNELIVANKALLAPLEDDLSGDDFGFNVNAMAGCGPSVLVDVLNLTAVAIRRLIKMSKKINAFK
NMCQDDQIALLKGGCTEMMILRSAMTYDPDRDSWKIPQSKEKLMNIKVDILKEAGGRVFEEHQRFLQTFDQKWRNDENI
MLILSAIALFTPDRPRVVHFDVIKLEQNSYYYLLRRYLESVYQGCEARLTFLKLIHKISELHKLNEDHVRVYLDANPREIEPLLIEIF
DLKPH*
>Ph411130-HR38
MPRGGFGNAMHVIWVVAPFGSSTFAELLSAPYSEDVGGETLDPFPEVVFHSQPLSPAPLPSFQEIYSPRFPTRQTKSPELNN
IGIKMDEDYVVYHHHHPSPAMPTTPIQSPYDFHSQHFSPYYQQQPHHPNQGYESSGGPQDSYSLPHFPTSELHINTTLNLR
QRRASLPLQRSESTNSSDSPKLRMGMMTGPPPSASSSASSSPGCGVPETVAPSLLPRPAPQSPSQLCAVCGDTAACQHYG
VRTCEGCKGFFKRTVQKGSKYVCLADKSCPVDKRRRNRCQFCRFQKCLAVGMVKEVVRTDSLKGRRGRLPSKPKSPQESPP
SPPVSLITALVRAHVDTTPDLANLDFSQYREPIPGEPPMSEAEKIQQFYSLLTTSVDVIRHFADKIPGFTELTKEDQDLLFQSAS
LELFVLRLAYRTKADDTKLTFCNGVVLDKHQCQRSFGDWLHAILDFCQSLHAMDIDISAFACLCALTLITERHGLKDPHKVE
QLQMKIIGSLRDHVTYNAEAQRKQHYFSRLLGKLPELRSLSVQGLQRIFYLKLEDLVPAPPLIENMFVASFGKKKTKITRNEDE
TREEPTESTAEIYLFTILGR*
>Ph331460-tll
MMMTMFTGRILYDIPCKVCQDHSSGKHYGIFACDGCAGFFKRSIRRSRQYLCKAKSEGSCTVDKTHRNQCRACRLKKCVE
AGMNKDAVQHERGPRNSTLRRQMAFYFKEPCDTTSDIGCGPSVSPILQHSQTHVLDLALPKIPTSVSPRSESNGSPLMQPT
MAAAFLFGQTLIPKLTPNFTLTNAIPLGSVEALCESAARLLFMNVTWAKNVPAFTRLNYKDQLLLLEESWRELFVLSASQFM
LPLELVDLLTAHTTVTANSEKALTIAQEVKQFQETLIKFKQLHVDIHEYACLRAIVLFKTSFDPSNNTVTSSTAPSSSGEGKTLH
DLAEVAAVQDHTQLTLNKYISAAHPTQPFRFGKLLLLLPSLRSVSNNTIEELFFRRTIGNIPIERIICDMYKASD*
>Ph318510-HR39

MDATATSTSWTQSASTTRLASQNSPTKGVVSVVVDSRNESPNVFAVKEEHIVGHRGTSITCGGVTVTSINVPQNEEQDFSS
QNVKNTLHFGANERAEIGIKCEPLSSNFNDDDSDVHLRIKKKRSNKNDEKSQDILKIEEPERPMSWEGELSDTEMSIVTDGR
RMRDDIKDELMSDVQMSPQSIPKSEIGDAIIMESGRLKQEPETCNETMSGYSSSSRELPLLVDKLLGGYSTPSQSPILQQRQ
QHHSLTKQHSLLGALPPNPSPDSAIHSAYSYSSPAASPAASRHLPSSGFSSPFTPSLSRNNSDASQYGGSQHSSCYSYNSENF
SSPTHSPIQSRHLLGYSKVDGSPIHSVTGGQLDYQQQHLDNERDEEKDPCLEEKMSCLASDFSQHSALATPPGISRQQLINS
PCPICSDKISGFHYGIFSCESCKGFFKRTVQNRKNYVCLRGSQCPVTIATRKKCPACRFEKCLKMGMKLEAIREDRTRGGRST
YQCSYSVPSSLLQNSDSIHKLSNSLTAGETLVKLETPHLGSPNSNLDSRLLPVPQLLQEIMDVEHLWHCNEREMLGSPMNN
HRHSPSSTPSSPSPSQSQTNSESNPDFLSNLCNIADHRLYKIVKWCKSLPLFKNISIDDQISLLINSWCELLLFSCCFRSMSSPG
EIRVSLGKSISLSKAKELGLGPCIERMLNFTQHLRRLRLDKYEYVAMKVIVLLTSDVNDLREPEKVRASQEKALQALQHYTLAH
YPDMPSKFGELLLRIPELQRTCQVGKEMLSIKNREGEEGPSFNLLMELLRGDH*
>Ph301950-HR78
MTAKYRSNDLEADIKMEQPPLNCAIKMEQPHLNYGIKMEQALDLNISSGLKVEQALDLNMSSNLKIEQAMDLNMSSDKV
RSLCLAVELCVVCGDRASGRHYGAISCEGCKGFFKRSIRKQLGYQCRGTKNCEVTKHHRNRCQYCRLQKCLEKGMRSDSVQ
HERKPISEKNSRDLSSLIASNNNSQPFLPTDINSKIYLRKDLENAPSAFAAAFGLADLGLLTATTLGKSSCKFIEIHLLIKYNMFV
HLGNEIVKRESPHMSDEEDSAESDVNEESGDALSWAREKTLKESLDLLSKLLNENYNANESATEEDRKPLDCDESLLQDYHS
TFNLQTPSPVPVHLNVHYVCESACRLLFLSIHWARSISAFQNLSHDLQVALVRRCWPELFALGLSQCSQILPLRAMLAPIIAYL
RTAVTQDTMAAQRLIDITDHIFKLHEYVTRMVSLQIDNHEYAYLKALVLFSWHLPGLPLALKRQIDRLQEKALQELKSYVAK
NNSEEDNRSSKLLLRLLPLRFLQPQIMEELFFAGLIGNVQIDSVIPYILRMEADEYRVSLLNEQASQGFET*
>Ph234430-Eip78C
MSRDSVRYGRVPKRSRERSTEEQSRVSTSDAEQSDSDTKQLAVYDVILTVSQAHHANCGYTEEHTRNLVRKPITIPPLNGSS
STDGPEVASSTAESLEQERIWLWQQFASHVTPSVQRVVEFAKRVPGFCDLSQDDQLILIKVGFFEIWLGHVSRQTCDSSMT
FTDGTYITKQQMDLIYDPEFVSSLFHFAASFNALALNDTELGLFSAVVLLSADRPGVADIKVIEQHQDRLIEALKVQVGRNHA
SDPQLFSSLLMKLPELRNLGAKHSAHLDWFRINWQLLRLPPLFAEIFDIPKCEEDLQ*
>Ph233230-Eip75b
MAFIPAKVARNETMQENRFPNNEFQNLSLDEKKGASRTTSSSSSSGDEGDRSSSSSSSDDEKCPHEFCDNDYDYDVDDAV
RFGRVPKREKARILAAMQQSTNSRSQEKAVAAELEDEQRLLGTVIRAHLDTCDFTKDKIEPMIARAREQPSYTACPPTLACP
LNPNPQPLSGQQELLQDFSKRFSPAIRGVVEFAKRIPGFSLLPQDDQVTLLKAGVFEVLLVRLACMFDSQSNSMICLNGQVL
KKESIHNTSNARFLMDSMFDFAERINSLGLSDAEIGLFCSVVVIAADRPGLRNTDLIERMHNKLRNSLQAVVTQNHPGHTDI
CNELMKKIPDLRTLNTLHSEKLLAFKMTEQQQLQQQQQLQQQEMWTEEEVHSKSPAGSSWSSSSDVTMDEAVKSPLGS
VSSTESVCSGEVAAITDHQGGPASTAPLLAATLAGGICPHRHRANSGSTSGDDDIMGSHLLQNGLTITAVNNPSKSYNQSH
RFHRKLDSPSDSGIESGTEKIDKVTNSVNSAPTSLCSSPRSSLEDKDEDKQHTHIVEDMPVLKRVLQAPPLYDTNSLMDEAYK
PHKKFRALRNKDSAEAEPMIVVSPVPSVNSSEHHSSTQLHRHLTATTSAPPSLTGSSLSSTHSTLAKSLMEGPRMTAEQVKR
QEIIHNYIMRGDPPAEFNQSSSSSSCPAPRPNNNNLLNCGGNWSQPQTGYHYLPTQPQPSRWHSNGASVITTSRPFSLTP
NSTSSPPPVLQKALEQPNAEFSRIYLQPQSPHLASSSTSPSPIARNVSPSSCSPASRSPSVAGGPQMTLPSPKMMELQVDIA
DSNQQPLNLSKKSPSPSPRPLSAPSTAVQKALTLEA*
>Ph195250-HR4
MTLTRNPCELDTMSLFQDLKLKRRKVDSRCSSDGESVAETSTSSPDMAGPPSPCPKLTSPSPEPETPRNRVRLSPEQPRIRSS
PEQRIRLSPEHPRVTSEQLKLRLSLEQSRIRLSPDQQPRVRLSPDQQPRLRLSPDQQPRVRLSPEQQQRLSPLIQKGSPVIQRS
MSGGEQNTSGGVLNMISPGAFWVNSAASGRINGVRPELIGGNVNMNFPVNSQNSDMKPPRPVSTPGTPTMTRQTPTVI
MGEAGGVRTMIWSQPNPSSPADHQNQNMAASTSAINWSTSPQSSAVIVVKNQRRNFYLISVNPNPLNMERLWAGDLT
QLPVSQQNQALNLALPPGDWPRDGRPQGITIIGEPKHDPEDDEQPMICMICEDRATGLHYGIITCEGCKGFFKRTVQNRR
VYTCVADGNCEITKAQRNRCQYCRFKKCIEQGMVLQAVREDRMPGGRNSGAVYNLYKVLMRERDNKFLNVIFNNNEKG
NQQKSSISSEQTLPSHIVNGTILKTALTNPSEVVHLRQRLDNTVSSSRDRTFPLDTILNMIQALVDCDEFQDIATLRNLEDLLD
HKSDLSEKLCQIGDSIVYKLVQWTKRLPFYLELPVEVHTRLLTHKWHELLVLTTSAYQAIHGVHKLSSTSTAGQEADFTQEVS
NNLVTLQTCLTSMMGRPITMDQLRQDVGLMVEKITHVTLMFRRVKLRMEEYVCLKVITMLNQGRGGSNELEAIQDRYM
MCLKSFVEHSFPQQPARFHELLLRLPEIQSAASLLLESKMFYVPFLLNSAIQR*
>Ph164330-usp
MMKKDKPMLSVAAIIQGVQVQHWSRGKTNVMMLLSVELGGPQSPLDMKPDTATLLGGNFSPNGGPNSPNSFTMGHS
SLIGNNSNNKMASYPPNHPLSGSKHLCSICGDRASGKHYGVYSCEGCKGFFKRTVRKDLTYACREERNCIIDKRQRNRCQFC
RYNKCLAMGMKREAVQEERQRTKEREQGEVESSGAMQADMPVERILEAEKRVECKVENQNEYENAVANICQATNTQLY
QLVEWAKHIPHFSSLPIEDQVLLLRAGWNELLIAAFSHRSVEVRDGIVLGAGITVHRNSAHQAGVGTIFDRVLTELVAKMRD
MNMDRTELGCLRSIILFNPEVRGLKSGQEVELLREKVYAALEEYTRVTRPEEPGRFAKLLLRLPALRSIGLKCLEHLFFFRLIGDI
PIDTFLMDMLGSSSDS*
>Ph053030-HR3

MFDMWNSVSPKLETSQPGTTSNSQLPQTSTNAAGSIKAQIEIIPCKVCGDKSSGVHYGVITCEGCKGFFRRSQSSVVNYQC
PRNKNCVVDRVNRNRCQYCRLQKCLRLGMSRDAVKFGRMSKKQREKVEDEVRFHRAQQRQATESTPDSSVFDQQTPSS
SDQLHYNGYSYNSSDVGSYTNYNYTPHHIQYDISADYVDSTTAYDPRPGLDSLTADSNLMTSVVTTGGNCHSGNNTVENQ
SNQRINSLNNQTTGNDRTERNGSNERTGLEGAQRQGLDRLVTVKQENVDNIIGGGGFVDSTTLLQPMSPSSQASQNLSPR
SQVSQTMTSPSVKLKEEEDINQGCPSKMYPMQISELLSKTIADAHSRTCVYTLEHIHEMFHKPQDLSKLIYYKNMAHEELWL
ECAQKLTTVIQQIIEFAKMIPGFMKLSQDDQIVLLKAGSFELAVLRMSRYFDLSQNCVLYADTLLPQDAFFTTDTSEMKLVTS
VFEFAKSVAELKLTETELALYSAVVLLSGDRPGLKGTTEINRLRHAVARALRFEMDRNHILPLKGDVTVYDHIMAKIPALRELS
LYHMDALAKFKRSTPLLEFPALHKELFSVDS*
>Ph039420-HR51
MGSSPECCSAGSSPAHPLPLCLGSCTPHHQTVVQAQRSPPSPFLLGHLPVNKGKILGLSCVVCGDTSSGKHYGILACNGCSG
FFKRSVRRKLIYRCQAGTGRCVVDKAHRNQCQACRLKKCLQMGMNKDAVQNERQPRNTATIRPEALVEMDQERALREA
AVAVGVFGVHIFLLKGSQSTKDKSINRDKSNHISISGRSVPPVSLAVGFSPVRYPQSLTSPSEVNSSKQEADEEDSIDVTNEES
LPPTPTSRTLPCTISTPTTHLPPPPLYTTTQETIYETSARLLFMAVKWAKNLPSFASLPFRDQVILLEECWSELFLLNAIQWCLP
VESSPLFSVNEHAATVPNGKSSQTAADIRVLNDMLLRYKAVGVDPAEFACLKAIVLFKSETRGLKDPLQVENLQDQAQVML
GQHARGQHPTQPARFGRLLLMIPLLKHVPTQRVEHIFFQRTIGNTPMEKVLCDMYKN*
>Phumsvp
MALVAPPPTAWREPGAMLGPGLGPIGPGPGPGPVTPTQAEDITALVPVVRELQQSATPGLQSNSTQSGGSNNGVDKNQ
NIECVVCGDKSSGKHYGQFTCEGCKSFFKRSVRRNLTYSCRGNRNCPIDQHHRNQCQYCRLKKCLKMGMRREAVQRGRV
PPSQPPGLPGLPDHRFIDRSIGHLLTRGQTLTSKIMDACGLSDVAHIESLQEKSQCALEEYCRTQYPNQPTRFGKLLLRLPSLR
TVSSQVIEQLFFVRLVGKTPIETLIRDMLLSGSSFSWPYMSSM
>Phumea
MWTYFFFFFNFQTMKICDAISFFGRTYNNLGSITECKNNGECVINKKNRTACKACRLRKCLLVGMSKSGSRYGRRSNWFKI
HCLLQEQSQNGTNRLNTNIFGSPPGFFPGSFLSNSLLSPDKFFQTAAAFHGYSSRSKKEEVSNHSESGESSAGDVDDESRSSS
ALNNYMRPPSSHKTPSPFSDKDYTQRNSAFSKHLSPFTPAVSLNLPTSPIGCTVTNSPFSPPVTGGGMFFPPSQPSPNRLLFP
SVVTKPGVVTPINPLLGSWPPPSMQNGGNLLLCSPALAGVAVEQEHPIDLSVKTSTISYNSPPTPVSNKDSDNYKENSDVD
DDDDDDLTIDVELCKESVLIKNNPLDLTAKRVEVPQSG
>ClecKnrl1
MNQLCRVCGEPAAGFHFGAFTCEGCKSFFGRTYNNLSSISECKNNGECVINKKNRTSCKACRLRKCLLVGMSKSGSRYGRR
SNWFKIHCLLQEQGGSGGAPTPPKWDEDNNNVKKEPPVWRLPIPGGPIFGFPPPIPPFFLPRGLLLPPPPPPTPPQPPAPAS
SLDILRSLGPVQDSPMDLRLRKDEESTQSDRKTPLDLTCVKT
>ClecHR96
MDNEERRSHEKFCSVCGDQALGYNFNAVTCESCKAFFRRNATKTRELQCPFKQCCDITAVTRRFCQKCRLAKCFRVGMKR
ELIMTEADKERKKRKILANKARMVTAKAKDSIGNSKSVQVSMVSVEIGTQTEPLTCNCRQRYKADVPNSLLMAALPPPDKT
QITYQLIKSIPTATLFPMSCQDNELLEDLIVANKALEAPLDQELNNLPGDEFKVCAFILYSCGSKSLLDVINLTALAIRRLIKMCK
RIGGFRALCQEDQLTLLKQGCTQMMLLRSVVTFDPDRNSWRIPHSVDQMSQINVEVLKEARGNLYDAHEAFLRTFDTRAS
HDLNIMCILTAIVLFDPNRTNLVNKQLIAQQQPDILLLRQISCYFQSRYFSLLQCYLQSIYSSEESNEIYQHLICKMTELHQIIEEH
VRVYLNVNPSQVEPILIEIFDLKTH*
>ClecEcR
MWVRGYAMREEGCDQVTSSCSPGVDDLELWDLGLGPGPRGIQLSEHRGDISGREDLSPPNSLNGYSADSCDGSKKKKGT
AVRQQEELCLVCGDRASGYHYNALTCEGCKGFFRRSITKNNVYQCKYGNNCEIDMYMRRKCQECRLKKCLSVGMRPECV
VPEYQCAVKRKEKKAQKDKDKPVSTTNGSPEAIKVEPEPHRVSYTSSLFQSMIKESQTQSTEGELAKVAVNGIKQVSAEQEE
LIHRLVYFQNEYEHPSDEDVRRINTPNDEEEQSDLKFRHITQITILTVQLIVEFAKRLPGFDKLLREDQIALLKACSSEVMMLR
MARRYDAQSDSILFANNQPYTRDSYNMAGMGDVVEGLLRFCRQMYNMKVDNAEYALLTAIVIFSERPSLTEGWKVEKIQ
EIYLEALKSYVDNRARPRSPTIFAKLLSVLTELRTLGNQNSEMCFSLKLQNRKLPPFLAEIWDVNP
>ClecHR39
MEGGVSVKWGEPTTRTGVVTSGQVTVTTINIHHSPHDKEENYYQPQCNGGSSKCSVVVSGEQMNVDEDSSAADAEVCN
FTSEPVNLRNKNRDESRGSIERPMSWEGELSDSEVTTIKPEPEENPIENSESGSNPKSSKIMAQIEDEKPITLKNEKITESAKTN
QFNAQSELPLLVDKLLSSGGVLKGPGPTPSPDSAVYSCYSPTASPVTSRHILSSSSGISSPFTPSLSRNNSDASQYGGSQHSSC
YSHSYSSVSVSPTQFSPTHSPIQGRHMHRPGFPSPVLNPRGVHEYSMQDEGGTLDDKYSSMTADLLQHSTGLASPGISRQ
QLINSPCPICGDKISGFHYGIFSCESCKGFFKRTVQNRKNYVCLRGSSCPVSIATRKKCPACRFDKCLNMGMKLEAIREDRTR
GGRSTYQCSYTVPAGLVEQKCAEPPPQATPQTNLVPPLLQEIMEVEHLWQYNEADPKLVGRIPRPQSGNPTDLMANLCNI
ADHRLYKIVKWCKSLPLFKNISIDDQTSLLINAWCELLLFSCCFRSMSSPGEIRVSLGKCISLTEAKNLGLGPPIERMLNFTEHLR
RLRVDRYEYVAMKVIVLLSSDTSDLREPEKVRASQEKALQALQHYTLAHYPDIPSKFGELLLRIPDLQRTCQVGKEMLSIKSKE
GEGPSFNLLMELLRGDH

>ClecHNF4
MFVRDVEDGEMGEGSLSVGGNTPVPNFPQSCAICGDRATGKHYGASSCDGCKGFFRRSVRKNHLYNCRFSRTCVVDKDK
RNQCRYCRLRKCFKAGMKKEAVQNERDRISCRRNSYDDQTIGNSLSVNSLLNAEILSRQTGAALEMSHDYDLSNKQVATIN
DVCESMKQQLLFLVEWAKFIPAFLELQLDDQVALLRAHAGEHLLLGLARRSLHLKNVLLLGNNCIIPRHTADAGGTADLDIS
RVGTRVMDELVRPLTEVQIDDTEFACLKAIVFFDPNAKGLTESGKVKFLRYQIQINLEDYISDRQYDSRGRFGELLLTLPALQSI
TSQMIEQIQFAKLFGVAKIDSLLQEMLLGGAPDVPAANGQTNSTPMNIVNYPSSGGSPESQNGLSPVHSPPQGSNTVLRDI
STSTQDYQYSFKQEPGLDV*
>Clectll
MDSRTKNHVNDRDDNAGRILYDIPCKVCQDHSSGKHYGIFACDGCAGFFKRSIRRNRQYVCKVKGEGGCLVDKTHRNQC
RACRLTKCLEAGMNRDAVQHERGPRNSTLRRQMCLLVKEEAGPSTPSMDLSAPNASPPHFSPFYTPQIGEVLSNPEAICES
AARLLFMNVRWAKNVPAFTGLNMKDQTTLLEESWRELFLLGWAQLLPLTDLAILVANSPRLLQEAATFQDTLAKLRSLGLD
HHEFACLRAVILFKTALEGEGKSLVDVASIAALQDQTQVTLNKYVSTAYPDQPYRFGKLLLLLPSLRLVSANTIEEMFFRRTIGP
IPIERIICDMYRSAGAI*
>Clecusp
LSLENNLALVGPQSPLDMKPDTASLLVGGFSPTGGTSGSTSPTTFNIGHSSVLGNGNKSTVVYPPNHPLSGSKHLCSICGDR
ASGKHYGVYSCEGCKGFFKRTVRKDLSYACREDKQCLVDKKQRNRCQYCRYQKCLSMGMKREAVQEERQRTKDRDQNE
VESTSSFHTDMPVERILEAERRVDCKIETPPIDFENQTNEICQATDKQLFQLVDWAKHIPHFTSLPLEDQILLLNAGWNELLIA
GFSHRSVTIRDGLVLGSGVTINRNNAHQAGMGTIYDRVLTELIAKMREMKMDKTELGCLRTIILYNPEVRGLKSVQDVEML
REKVYAALEEYSRMSHPDEPGRFAKLLLRLPSLRSIGLKCQEHLFFYRAIGDVSVDTFLLQMLESPERL*
>ClecHR38
MKATRPNIITSKQNKTARIGGSSSSDSPKLRQGTTPCPSASVSSSASSSPTERPNAISPSQLCAVCGDTAACQHYGVRTCEGC
KGFFKRTVQKGSKYVCLAEKSCPVDKRRRNRCQFCRFQKCLSVGMVKEVVRTDSLKGRRGRLPSKPKSPQESPPSPPVSLIT
ALVRAHLDTSPDISNLDYSQYREPNEEDVQMSETEKTQQFYNLLTSSVDVIRHFAEKIPGFSELCREDQDLLFQSASLELFVLR
LAYRSRVVDMKMTFCNGVVLSRNQCQRSLGDWLHPILEFCQSLHAMEIDISSFACLCALTLITVSMSLNLY*
>ClecPNR
MLSMQEEGKEAYCKVCGDKASGKHYGVASCDGCRGFFKRSIRRNLDYVCKEEGRCVVDVTRRNQCQACRFSKCLRVNMK
KDAVQHERSPRSNSATPSLSFVYQQFLPYPRAYLHPASIPGLQYMGCYMRSECNENEVSSSQESTESRLEPSVSLLSEDIYSTS
SRLLSLTVQCVRAIPSFQQLSPQDRDSLLEDSWKDLFILTLAQWVTYLAPIQFLSGFFVLFMTFIISLLVLLEKLIRDTGIDDASR
LDELRKDLKVVSYAIARLTQLRADHTEFACLKALVLFNPESVRKEEVEVLQEQTHVMLAEYSGVRSAKLLLLLLNTSRTSLKNIL
LLFFNRHAPVRLEGLLASK*
>ClecHR4
GLSPECGGRKGGEVRVKEELVFDGGGPAPATSGSGYWPPQQQQQQQPQQHQAQQQNPRVNGVRHELIGNEMRPPQ
APTPRPAPTVLMGEVGGVRTMVWSQPAPAEQPTTSSWPPPNQEETAAQLLLTLGQETSPNTTGTRALNMERLWAGDLS
QLPGAQQITALNLSWAKQQPTKPDDHEDDEQPMICMICEDKATGLHYGIITCEGCKGFFKRTVQNRRVYTCVADGVCEIT
KAQRNRCQYCRFKKCIEQGMVLQAVREDRMPGGRNSGAVYNLYKVKYKKHKKSVRNGQIKSVNEKGKPAISPEHSIPPHL
VNGTILKTALTNPSEFMCTMVVHLRQRLDNAVSSSRDWALPIDATLSMIQTLIDCDEFQDIATLRNLDDLLEHKSDLSDKLM
QIGDSIVYKLVQWTKRLPFYLELPVEVHTRLLTHKWHELLVLTTSAYQAIHGAHKLASTGSDGTEAHFTQEVSNNLCTLQTCL
TSMMGRPITMDQLRQDVGLMVEKITHVTLMFRRIKLTMQEYVCLKVIIMLNPARGGSNELEAIQERYMTCLRTYVEHNSP
NNPNRFHDLLVRLPEVQSAASLLLESKMFYVPFLLNSTIQRX
>ClecKnrl3
MPKENEKKKERKRERERKQFTFFNYVHKSKSVDRQARNYTTAKLDEPRMNQQCKVCNEPAAGFHFGAFTCEGCKSFFGRT
YNNLSSISECKNNGECVINKKNRTSCKACRLRKCLLVGMSKSGSRYGRRSNWFKIHCLLQEQGGLPAVKKEDEEEEAQKERR
FTPPPPFYPQPRRHFLFPQEPWQPWPPTPPEAPQDQPIDLSLKPRTDSEVLDLTKRQALAGWSS*
>ClecKnrl2
MNQQCKVCGEPAAGFHFGAFTCEGSILATRIEFFRLAYKRKGVVCLKFTFPQFLGLLIPRARVFPVRPCATPQLAKSVLDLDV
VIQLPSFLFQSFFGRSYNNLSSISECKNNGECVINKKNRTSCKACRLRKCLLVGMSKSGSRYGRRSNWFKIHCLLQEQKQKND
DGRLGAQIASINRNKEELMLLGLEDYKTYSPSTSSPGSDTSEEKCRREPPYFGFPIIPQMFLPQPFHPAFLPSPYLYQNRTWD
ESPIDLSTKSAEDSTDSDEDINVDDLPEEPSIIRAPLDLTTKVV
>ClecHR3
MVKHPSGTYNRRNDSTPLECPGCDPSLLEYAFPNSQWSASRGTVKGRTTPVATPRSHPAAAVPSLEQSVGGVIGVISWVR
RRQKLVKMAKCRRNAQAQIEIIPCKVCGDKSSGVHYGVITCEGCKGFFRRSQSSVVNYQCPRSKACLVDRVNRNRCQYCRL
QKCLRLGMSRDAVKFGRMSKKQREKVEDEVRYHRAQLRAQVEQTPDSSVFDQAQTPSSTDQLHNYTGYSAYGSDVGSYS
YNYTGQVTATMQYDISADFVDSTTTAYDPRPSIDQMSESSMMSANNVSTGGGVGKVVQGQQRLAIKLESEPISDSIVNTF
VDSTTSRQSEDVSPKESESNSNSYTSSLIDPAQISELLSKTIADAHARTCLYSIEHIHNMFRKPQDLSKLIFYKNMAHEELWLEC

AQKLTTVIQQIIEFAKMVPGFMKLSQDDQIVLLKAGSFELAILRMSRYFDLSSGWVLYGDTMLPQDAFYTTDTAEMKLVTL
AFEVSSGVAELKLTETELALYSACVLLSADRPGLKGLAEIGRLGQAVLRALRIELERNHTTPIKGDVTVCDALLAKIPTLRELSML
HMEALGKFKRSTPHLEFPALHKELFSVDS*
>Clecdsf
MVLIDPFVQGDRLLDIPCKVCGDRSSGKHYGIYSCDGCSGFFKRSIHRNRVYTCKAQGDLKGRCPIDKTHRNQCRACRLTKC
FQSAMNKDGMGGTIAVQHERGPRKPKQSKDGESPVRQVSGNNGVCLESSQHSPLKLTSPAYQQAPMQQADSPERRPQ
PLFVSHQPPGLLQLLMSAEKCQEIVWNGKLDVINGGNSGPGSPSATAIPLSLSPSWEVLQGSICSQETTARLLFMAVRWVR
CLAPFQTLSKRDQFLLAVNLTSMCIYLQLLLLQESWKELFLLHLAQWSIPWDLSPVLGGPKARERLPLDDTLVPNELNAIQEIL
ARFRQLSPDGSECGCMKAVILFTPETPGLVDVQPVEMLQDQAQCILNDYVRGRYARQPTRFGRLLLMIPGLKVIRQSTIERL
FFRETIGDIPIHRLLGDMYVMEKSYS*
>ClecE75B
MLFDTRVPQYHGQYFAPGLPPQLPEAQPQQADHQLNIEFDGTTVLCRVCGDKASGFHYGVHSCEGCKMGCLCDQGFFR
RSIQQKIQYRPCTKNQQCAILRINRNRCQYCRLKKCIAVGMSRDEQFEPTRTHCFTNVSLDLFPKRVDNNTKDFQQPPFNK
KRPVMMTEELPILKGILNGVVNYHNAPVRFGRVPKREKARILAAMQQSTNSKCHEKALAAELEDDQRLLRTVIRAHLDTCD
FTRDKVEPMILRAREQPSFTASPPTLACPLNPNPQPLTGQQELLQDFSKRFSPAIRGVVEFAKRIPGFGILSQDDQVTLLKAG
VFEVLLVRLACMFDTQNNSMICLNGQVLKRESIHSGSNARFLMDSMFDFAERLNSLRLTDAEIGLFSSIVVIAPDRPGLRNTE
LIERMQNKLKAGLQLMMSQNHPNQPCLAQELMKKIPDLRTLNTLHSEKLLAFKMTEQQHLAEQQQMWGTVVSEDESKS
PSGSTWSSSSDVAMEEVKSPLGSVSSTESMCSGEVEYHSSHAASAPLLAATLAGGCPMRHRVGVEDNKDIKPIIIHRTFRKL
DSPSDSGIESGTEKVDKLTSSAPTSLCSSPRSSVEDKEDHQIEDMPVLKRVLQAPPLYDTNSLMDEAYKPHKKFRALRKECGD
EPDNTTSTSSLSSTHSTLAKSLMEGPRMTAEQMKRTDIIHNYIMRGEGCGWQQQQSVITTSSAARGGAYVIVNNSSPGPSI
YVPAGSPCPSSTSLVELQVGTQPLNLSKKTPPPSPRPKVLSLEA
>Clecsvp
MEMIDDKCVLREHDKKLLKYSVGGVLCGGGASSGAPPYHHVPPEFPPRAIVPWRDPSLVTLTQEDTLVSQAIRGDTPGTNS
TQSGGSQADKNQNIECIVCGDKSSGKHYGQFTCEEEKTLPDEFYLIKLRTIIKMLLCFGFAAVQRGRVPPSQPPSLPGQFALT
NGDAMSAAAAAAAAAAGFNGHSYLSSYISLLLRAEPYPTSRYGQCMQPNNIMGIDNICELAARLLFSAVEWARNIPFFPDL
QVTDQVALLRLVWSELFVLNASQCSMPLHVAPLLAAAGLHASPMAADRVVAFMDHIRIFQEQVEKLKALHVDSAEYSCLK
AIDMKKLDISSERVPVSILLESVLVKVSLTVSAHGPVAKRGLDACGLSDVAHIESLQEKSQCALEEYCRTQYPNQPTRFGKLLL
RLPSLRTVSSQVIEQLFFVRLVGKTPIETLIRDMLLSGNNFNWPYTMX
>ClecFtz
MGDDDMTVSLPTSSAQETNSEIEQINTHDTETEELQVSFDNNSTGTGVGMELPAGGASSFQQQPQMLQQAVSDMPDT
KEGIEELCPVCGDKVSGYHYGLLTCESCKGFFKRTVQNKKVYTCVAERSCHIDKTQRKRCPFCRFQKCLEVGMKLEAVRADR
MRGGRNKFGPMYKRDRARKLQMMRQRQLAAQTLRNSSGYSPGGLGDAVTLSYQPGSNFASLHIKQEIQIPQVSSLTSSPE
SSPSPIAVALGQAANGNISNNNMCSVMPGSQTQPALQIVNDQRISHQSVQAFEHAKLWSGNTSPPPSKTFQYEGSSSQS
QSKVSPMIRDFVQAIDDHEWQNSLYNLLQNQTYNQCEVDLFELMCKVLDQNLFSQVDWARNSVFFKDLKVDDQMKLLQ
HSWSDMLVLDHMHQRMHNSLPDETTLPNGQKFDLLSLGLLGVPSLSEPFTEITTKLQDLKFDLSDYICIKFLLLLNPEVRGL
MNRKHVQEGHEQVQQSLHDYCLTTYPQVQDKFNKLLQILPEIHRLATTGEEHLYVKHCNGSAPTQTLLMEMLHAKRK
>ClecHR51
MTTRWRVEDYSSRWNCGPQTVVRATRSPPPPQLGGGKLKSLGLSCVVCGDTSSGKHYGILACNGCSGFFKRSVRRKLIYRP
SSSMSLGLSMQYKNMISANASPGQEEINAVQPRSQYSQPSPPKSSRDENEDEDTIDVTNEEPPATGTYSMSASAPPLPPEY
VFYPTMPETVYEVAARLLLMGVKWAKNLPSFASLPFRDQVILLEECWAELFLLNAIHWCLPLETCKLFSVPEHVMAAPSGKE
GLVAHEVRALNDALQKFKAIRVDAGEFACLKAIVLFKSETRGLKDPMQVENFQDQAQVMLCQHSRTNHPDQGQRFGRLL
LMLPLLKVVPTNRIEDIFFQPTIGNTPMEKVLCDMYKG*
>ClecHR78
MEKHAESSGIMNANSDKIHSLCLGVEICVVCGDRASGRHYGAISCEGCKGFFKRSIRKRLGYQCRGNQQCEVTKHHRNRC
QYCRLQKCLTMGMRTVQHERKPISVKKEFPNHSSPFYKSLTQPSNIGQSNYGVYNSLPYNLYSDGYIKQESSNIIGNYDYSNY
DESSDSDLIESIGPGQDSKSMINSAMEIATKLGMNGNFGNSDDEEMENLQFDGRLLDDNAFIFQIQSPSPMPAYTDVHYIY
ESASRLLFLSVHWVRNVPAFQILNMDTQVSLIKGCWSELFTLGLSQCAQVLALPTIILSIINHLQSSVAQQKISSSKVKAVTEHI
FSLQDFVGSMVALNVDDHEYAYLKLIALFSPDNPSLHIRRQLSELQEKAFQELREQIGDVNSNRFARLLLRLPPLRALNRHIM
EQIFFPGLGDQCDIDNIIPFILKMESIDFVNEQNN
>ClecE78C
MSRDSVRYGRVPKRSRERSGGEERVSTSDSSSGATPPDPETTSTPAYDLIVLEQQRIWLWQQFATHITPSVQRVVEFAKRV
PGFCELSQDDQLILIKVGFFELWLSHAARLTTDTTVTFSDGTFVTRQQMELMYDVSPTQSEFVTSMFEFTSSFNSLLLGDTEL
GLFSSIVLLSPDRPGVTDVKAVEHHQDRRSTNGTDSSSVLAKLPELRALGAKHALMLEWFRLNWDKLRLPPLFAEIFDIPKSE
EDL

>Ph460980-HR96
MNSRASSRELNDAERAKLNELIVANKALLAPLEDDLSGDDFGFNVNAMAGCGPSVLVDVLNLTAVAIRRLIKMSKKINAFK
NMCQDDQIALLKGGCTEMMILRSAMTYDPDRDSWKIPQSKEKLMNIKVDILKEAGGRVFEEHQRFLQTFDQKWRNDENI
MLILSAIALFTPDRPRVVHFDVIKLEQNSYYYLLRRYLESVYQGCEARLTFLKLIHKISELHKLNEDHVRVYLDANPREIEPLLIEIF
DLKPH*
